# Learning to learn from data: Using deep adversarial learning to construct optimal statistical procedures

**DOI:** 10.1126/sciadv.aaw2140

**Published:** 2020-02-26

**Authors:** Alex Luedtke, Marco Carone, Noah Simon, Oleg Sofrygin

**Affiliations:** 1 Department of Statistics, University of Washington, Box 354322, Seattle, WA 98195, USA.; 2 Vaccine and Infectious Disease Division, Fred Hutchinson Cancer Research Center, 1100 Fairview Ave. N., Mail Stop E5-110, Seattle, WA 98109, USA.; 3 Department of Biostatistics, University of Washington, Box 357232, Seattle, WA 98195, USA.; 4 Division of Research, Kaiser Permanente, 2000 Broadway, Oakland, CA 94612, USA.

## Abstract

Traditionally, statistical procedures have been derived via analytic calculations whose validity often relies on sample size growing to infinity. We use tools from deep learning to develop a new approach, adversarial Monte Carlo meta-learning, for constructing optimal statistical procedures. Statistical problems are framed as two-player games in which Nature adversarially selects a distribution that makes it difficult for a statistician to answer the scientific question using data drawn from this distribution. The players’ strategies are parameterized via neural networks, and optimal play is learned by modifying the network weights over many repetitions of the game. Given sufficient computing time, the statistician’s strategy is (nearly) optimal at the finite observed sample size, rather than in the hypothetical scenario where sample size grows to infinity. In numerical experiments and data examples, this approach performs favorably compared to standard practice in point estimation, individual-level predictions, and interval estimation.

## Teaser

Optimal statistical procedures are constructed using deep adversarial learning to optimize play in a two-player game.

## INTRODUCTION

### Motivation and background

In most scientific disciplines, hypotheses are evaluated by applying statistical tools to experimental or observational data. Hence, the science of statistics plays a fundamental role in the process of scientific discovery, and innovations in statistical methodology have the potential to enable advances in the broader sciences.

Two distinct paradigms dominate the statistical landscape: the frequentist and Bayesian approaches. In the frequentist paradigm, probability statements describe the behavior of statistical procedures over independent repetitions of an experiment. It is common for unknown quantities to be estimated using maximum likelihood estimators (MLEs), whose implementation involves solving an optimization problem. In Bayesian statistics, the investigator specifies prior beliefs in terms of a probability distribution on the mechanism that generated the data, and data are used to update these beliefs. It is then common to use Bayes procedures, which summarize these updated beliefs about the unknown quantity. Except in special cases, evaluating a Bayes procedure requires sampling from an intractable probability distribution, thereby necessitating the use of Markov chain Monte Carlo methods or other approximation techniques (*1*).

For any given problem, different statistical procedures may be available, each with their own set of operating characteristics and performance that depends on what the true data-generating mechanism is. In the frequentist framework, performance can be adjudicated in a number of ways. It is often of interest to select procedures with best worst-case performance—this optimality criterion is referred to as minimaxity. The worst case refers to sampling from the data-generating mechanism under which the performance of the procedure over repeated experiments is least desirable. Minimax procedures are typically equivalent to Bayes procedures derived from a least favorable prior, that is, a prior under which learning about the quantity of interest is most difficult (*2*). This duality is often leveraged to construct minimax procedures. In most problems, analytically deriving a least favorable prior is intractable. Because of this barrier and the scarcity of alternative approaches to analytically deriving a minimax estimator, they are rarely used in practice. We note here that, although MLEs and commonly used Bayes procedures enjoy several prior-agnostic asymptotic optimality properties (*3*), these optimal properties do not hold in finite samples. Notably, these approaches are not generally minimax.

### Existing work in learning minimax procedures

Several authors have proposed to derive minimax statistical procedures by numerically specifying a least favorable prior. Nelson described an algorithm to iteratively construct unfavorable priors as a mixture between the current prior and a numerically derived less favorable prior, such as a prior degenerate on a distribution where the current Bayes procedure underperforms (*4*). A similar algorithm was proposed independently by Kempthorne (*5*) for the special case that the statistical model is one-dimensional. This latter algorithm iteratively updates discrete priors with finite support and reduces computational burden by allowing the support to shrink at each iteration. Both of these algorithms have been proven to converge to minimax procedures as the number of iterations tends to infinity. However, neither performance guarantees for any given iteration nor rates of convergence have been established. Other works have also proposed algorithms for identifying least favorable priors by optimizing over discrete priors with growing support (*6*, *7*). In another line of research, the authors have developed automated methods when the least favorable prior is sought over a restricted class, such as priors that only place mass on a prespecified finite set of distributions, both for general statistical decision problems (*8*) and for constructing confidence intervals (CIs) of minimal size (*9*, *10*). Given a rich enough set of support points for the prior, these three works note that the derived statistical procedures are nearly minimax. All of the above approaches require the ability to readily derive the Bayes procedure for any given prior. This computation is required in a critical step of these algorithms, which must be repeated over a large number of iterations. As is noted in the conclusion of (*5*), these evaluations become extremely computationally expensive as the size of the parameter space increases, even in one-dimensional parametric models. Consequently, it does not appear possible to use these existing approaches in many problems of substantive interest.

### Contributions and organization of this article

In this work, we present a new approach for numerically deriving minimax statistical procedures. Our approach is inspired by the recent success of AlphaGo Zero in mastering the game of Go. This algorithm was trained by playing millions of games against itself and self-improving after each game. Following this self-play, AlphaGo Zero became the world’s leading Go algorithm. AlphaGo Zero was trained using principles from deep reinforcement learning, where the player’s choice of move was parameterized using a deep neural network (*11*).

In our proposal, we leverage Wald’s formulation of minimax procedures as optimal play by a statistician in a game against Nature’s adversarial choice of data-generating mechanism (*2*). In this sense, a minimax procedure arises naturally as an optimal strategy in an asymmetric two-player game. We adopt an adversarial learning strategy, wherein datasets are iteratively simulated and the statistician’s play is improved over repetitions of the game. We refer to this strategy as adversarial Monte Carlo meta-learning (AMC). In our numerical experiments, we parameterize the statistician’s strategy using a neural network that takes as input the dataset and outputs a decision—e.g., an estimate or a CI. Our parameterization of statistical procedures is reminiscent of the recent approach of parameterizing optimization procedures using long short-term memory (LSTM) networks (*12*). Although neural networks are commonly used in statistics to build prediction functions in regression or classification settings, to the best of our knowledge, our work is the first to use neural networks to parameterize the statistical procedure itself. In contrast to existing approaches for numerically deriving minimax procedures, our approach does not require the explicit computation of a Bayes procedure.

Our approach bears some similarity to recent developments on meta-learning in supervised learning problems (*13*, *14*). In most of these approaches, existing datasets are used to tune or select among existing supervised learning procedures. A recent work proposes to incorporate an adversarial strategy when selecting the existing datasets used to tune the existing supervised learning procedure (*15*). There has also been some work in using existing datasets to construct classification procedures via grammar-based genetic programming (*16*). In contrast to all of these existing meta-learning approaches, in our proposal, a data-generating mechanism is adversarially selected from the entire statistical model. Furthermore, in contrast to all of these approaches except that of (*14*, *16*), our learning procedure is constructed from scratch rather than by tuning user-specified procedures.

The remainder of this paper is organized as follows. We start by introducing notation and formalizing the discussion of minimaxity given above. We then present our results. We first describe how the performance of a statistical procedure can be evaluated. Next, we describe how to iteratively learn a minimax procedure based on performance evaluations. We then present numerical experiments testing the feasibility of our approach and subsequently demonstrate the performance of our learned procedures in applications to real datasets. We subsequently provide theoretical guarantees for a special case of our approach and then provide some concluding remarks. Materials and Methods contains pseudocode for our proposed algorithms, details for the numerical experiments and data applications, and proofs of our technical results.

### Statistical procedures and the minimax principle

We now introduce key concepts and notation. Throughout, we suppose that an observed dataset *X* is randomly drawn from an unknown distribution *P*. We will illustrate our general framework in the context of three classes of statistical problems: point estimation, prediction, and confidence region construction. Point estimation involves obtaining an estimate of a summary S(P) of *P*. Prediction involves learning a function that predicts an outcome based on a set of individual-level covariate information. Confidence region construction involves finding a data-dependent set that contains S(P) with some prescribed probability over repetitions of the experiment. In each of these problems, a decision procedure *T* takes as input a dataset *X* and outputs a point estimate, prediction function, or confidence region. The procedure may make use of any knowledge that the statistician has about restrictions on the statistical model P of distributions to which *P* belongs.

To quantify the performance of a procedure *T*, we may consider a risk R(*T*, *P*), which represents a numerical measure for which smaller values indicate better performance. For example, in point estimation, a commonly used risk is the mean squared error (MSE), defined as the average squared deviation of *T*(*X*) from the summary S(*P*) over draws of *X* from *P*. For a given procedure, we define the maximal risk as the largest risk for the procedure *T* over all distributions in the statistical model. This often corresponds to the risk at a least favorable distribution that maximizes the risk R(*T*, *P*) over distributions *P* in the model. A procedure that minimizes the maximal risk is called a minimax procedure and has minimax risk

$$\underset{T\in T}{\text{min}}\underset{P\in P}{\text{max}}R(T,P)$$ (1)

Here, T is the class of possible procedures. For example, T may be taken to be a neural network class. We focus on these classes in our numerical experiments.

As indicated in Motivation and background, there is often a duality between minimax procedures and Bayes procedures under a least favorable prior. For each prior Π over distributions in the statistical model and each procedure *T*, the Bayes risk *E*_Π_[R(*T*, *P*)] is defined as the expected risk of *T* averaged over draws of *P* ~ Π. For a given prior, a procedure is Bayes if its Bayes risk is minimal over T. A prior is called least favorable if it maximizes min*_T_E*_Π_[R(*T*, *P*)] as a function of Π, that is, if it is a prior for which the Bayes risk of the Bayes procedure is largest. Under conditions, a Bayes procedure under a least favorable prior is minimax optimal (*2*).

## RESULTS

### Interrogating the performance of a given statistical procedure

The ability to assess the performance of a given procedure is critical, both to adjudicate an existing procedure and, as we will show, to construct an optimal procedure. It is common practice to evaluate the performance of newly introduced statistical procedures—a recent survey of top-tier statistical journals showed that most of their published manuscripts include simulation studies (*17*). Despite guidelines recommending the use of experimental design principles in these studies, most authors selected several distributions in an ad hoc fashion and evaluated procedure performance only at these distributions. A disadvantage of this common practice is that strong performance at the selected distributions may not imply strong performance at other distributions. Therefore, it would be preferable to systematically assess the procedure’s performance across distributions in the model. We refer to performing this assessment as interrogating the procedure. We note that interrogating a procedure *T* is equivalent to the existing notion of evaluating and summarizing the risk surface R(*T*, *P*) as a function of *P*—we introduce this terminology because it will play a critical role in our proposed procedure, and therefore, having this more compact expression will prove useful.

For procedures with theoretical performance guarantees, performing well in simulations is often treated as supporting, rather than primary, evidence of the procedure’s utility. However, for procedures without these guarantees, numerical results play a key role in establishing satisfactory performance at all distributions in the model. A natural performance criterion for evaluating this objective is the maximal risk. As indicated before, the maximal risk of a procedure *T* can be determined by finding a least favorable distribution. In practice, it suffices to identify an unfavorable distribution that nearly maximizes the risk. If the model is parametric, in the sense that each distribution in the model is smoothly determined by a multidimensional real number, then an unfavorable distribution can be found via standard optimization approaches. In general, performing this optimization requires evaluating a procedure on many simulated datasets drawn from different distributions in the model. A key challenge is that the maximization problem is generally nonconcave, and hence, finding the exact solution can be very challenging. This optimization is generally non-deterministic polynomial-time (NP) hard. In our numerical experiments, we use a variety of strategies to mitigate this challenge when interrogating our final procedures. In low-dimensional models, we use a fine grid search, whereas in higher-dimensional models, we use several global optimization strategies, each with multiple initializations.

We note that here we have focused on the use of simulation studies in settings where the investigator believes that the distribution belongs to the specified statistical model P. In these cases, simulations are often used to evaluate the performance of asymptotically motivated methods in finite samples. Another common use of simulation studies is to evaluate the performance of a procedure when the true distribution does not belong to P but instead belongs to a richer model $P_{1}$. In principle, an interrogation strategy could also be used in these settings, where the investigator would then seek to systematically assess the performance of the procedure over P but instead belongs to a richer model $P_{1}$.

### Constructing an optimal statistical procedure

We now present three numerical strategies for constructing minimax statistical procedures. The three strategies differ in the manner in which they approach the optimization problem in Eq. 1.

The first strategy involves iteratively improving on the maximal risk of a statistical procedure *T*. Using the maximal risk returned by an interrogation algorithm as objective function on the class T of allowed procedures, the procedure can be updated by taking a step away from *T* in a direction of descent. We can do so by first identifying an unfavorable distribution *P_T_* via the interrogation strategy and then updating *T* by taking a step in the direction opposite to the gradient of the risk of *T* at *P_T_*. Last, to pursue the more ambitious goal of constructing a procedure with lowest maximal risk, any candidate procedure could be iteratively updated to improve its performance. We will refer to approaches that iteratively update a procedure against unfavorable distributions for the current procedure as nested minimax algorithms. To feasibly implement these algorithms, a computationally efficient interrogation strategy would be required.

Nested minimax algorithms converge to the minimax optimal procedure under some conditions. For example, later in this work, we establish that, provided the class T of allowable procedures is unrestricted and the risk at each distribution is convex as a function of the procedure, iteratively updating an initial procedure via stochastic subgradient descent (*18*) will yield a procedure with nearly optimal risk. In particular, we provide a finite-iteration guarantee that shows that, after *K* iterations, the procedure’s maximal risk will be at most order *K*^−1/2^ log *K* larger than the minimax risk. Our results also provide certain guarantees for cases in which the interrogation strategy is only able to approximate the maximal risk up to some error. In addition, we establish convergence to a local minimum for nonconvex risks, which suggests that selecting many initial estimators and updating with subgradient descent until convergence should allow the statistician to identify a collection of local minimizers and select the procedure that performs best in interrogations. This is discussed in detail in the Supplementary Materials.

The second strategy for numerically constructing a minimax procedure leverages the fact that a minimax procedure corresponds to a Bayes procedure under a least favorable prior. As noted earlier, this has been previously proposed by several authors. The scheme suggested is to begin with an initial prior Π_0_ and then iteratively augment it to Π_*k* + 1_ by mixing the current prior Π*_k_* with the prior Π at which the Bayes procedure under Π*_k_* has the highest Bayes risk. In practice, this has been operationalized by taking Π to be degenerate at a least favorable distribution identified by interrogating the Bayes procedure under Π*_k_*. We refer to approaches pursuing this strategy as nested maximin algorithms. A challenge with existing methods following this path is that, for each candidate least favorable prior and each simulated dataset, the posterior risk minimizer must be computed. In most described implementations of these methods, the learned unfavorable prior is discrete with support growing over iterations. When this support does not contain many points, the posterior risk minimizer can often be computed explicitly. In many problems though, the support of an unfavorable prior must consist of many points, and consequently, some form of approximation or a computationally expensive Markov chain Monte Carlo scheme will generally be needed. In the Supplementary Materials, we highlight how prohibitive the computational cost of such an approach may be by showing that the number of support points in the least favorable prior grows exponentially in the dimension of the parameter space in a simple example.

The third strategy for numerically constructing a minimax procedure involves hybridizing the nested minimax and maximin algorithms. In these alternating algorithms, the current procedure and prior are iteratively updated by alternately (i) taking a gradient step to improve the procedure by reducing its Bayes risk against the current prior and (ii) taking a gradient step to improve the prior by increasing the Bayes risk of the current procedure. This denested algorithm is reminiscent of the approach pursued when optimizing generative adversarial networks (GANs) (*19*), which are often used to generate photorealistic images based on a set of available images. The optimization problem in GANs is framed as an asymmetric two-player game, similarly as in our alternating algorithm setup. However, in GANs, a generator is trained to produce synthetic samples that a discriminator cannot distinguish from the observed samples, whereas in our problem a statistician is trained to select a procedure that obtains low Bayes risk against Nature’s choice of prior. Thus, we emphasize that, despite apparent similarities, our proposed alternating algorithms are solving a different problem than do GANs and can be seen neither a special case nor a generalization of GANs.

To avoid computational difficulties arising from deriving Bayes procedures, our alternating algorithm does not explicitly compute posterior risk minimizers. Instead, the procedure is actively learned and updated over time and need not correspond to the minimizer of a Bayes risk. Another benefit of this approach is that it enables the learned procedure to take advantage of existing statistical procedures. For example, when estimating the coefficients in a linear regression, our procedure can include as input both the data and the ordinary least squares (OLS) estimator. In one of our numerical experiments, we provide preliminary evidence that including an existing procedure as input can expedite the convergence of our learning scheme. Because the Bayesian posterior is invariant under augmentations of the data with summaries thereof, it appears to be difficult for existing nested maximin algorithms to benefit from the extensive array of existing statistical procedures.

**Fig. 1:**
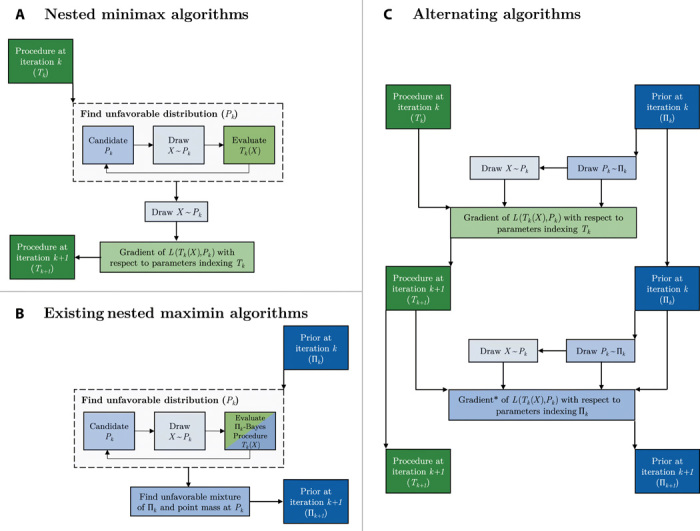
Schematic overview of algorithms for constructing optimal statistical procedures. Overview of an iteration of (**A**) nested minimax algorithms, (**B**) nested maximin algorithms, and (**C**) alternating algorithms, all in the special case where the R(*T*, *P*) = *E_P_*[*L*(*T*(*X*), *P*)] for some loss function *L*. Green boxes involve evaluating or updating the statistical procedure, and blue boxes involve evaluating, updating, or identifying the least favorable distribution or prior. Shading is used to emphasize the similarities between the different steps of the three learning schemes. More than one draw of *X* ~ *P_k_* can be taken in each step. In this case, the resulting loss functions *L*(*T_k_*(*X*), *P*) are averaged. Similarly, more than one draw of *P_k_* ~ Π*_k_* may be taken for the alternating algorithm. *This gradient takes into account the fact that *P_k_* depends on Π*_k_*, and *X* depends on Π*_k_* through *P_k_*. Details on how this dependence can be taken into account are given following the presentation of the pseudocode in Materials and Methods.

A schematic depiction of the three discussed strategies for learning a minimax procedure is shown in Fig. 1. We note that parts of this figure are oversimplifications—for example, the alternating algorithms implemented in our numerical experiments generally make several gradient updates to the procedure for each update to the prior. In addition to the schematic depiction given in Fig. 1, pseudocode for our proposed algorithms is given in Materials and Methods. This pseudocode provides more details on how these algorithms can be implemented in practice. Because all of these strategies aim to learn an optimal statistical procedure using datasets that were adversarially generated by Nature, that is, all of these strategies aim to solve the optimization problem in Eq. 1, we refer to the general framework encompassing these strategies as AMC.

Motivated by the recent strong performance of neural network architectures in a wide variety of challenging tasks (*11*, *20*), we have found it effective to parameterize the class T of statistical procedures as an artificial neural network. Our preliminary numerical experiments use simple multilayer perceptron networks (*21*), and for more challenging architectures, we benefitted from using a variant of LSTM networks (*22*). We discuss the rationale for using this architecture in Supplementary Appendix C. In our numerical experiments, we use Adam (*23*) to update the network weights.

Given how computationally prohibitive nested maximin strategies are, in this work, we focus on the proposed nested minimax and alternating algorithms. When possible, we will use the alternating algorithm—in contrast to nested minimax strategies, this algorithm avoids the need to identify an unfavorable distribution for the current procedure *T_k_* at each step *k*. In certain cases, we found that alternating algorithms lead to undesirable equilibrium points—we note that a user can easily recognize statistical procedures that correspond to these undesirable equilibria by using essentially any interrogation strategy. In our experiments, we used nested minimax algorithms in settings where alternating algorithms exhibited this poor behavior. When implementing the alternating algorithm, we must specify a form for the prior distribution. In each gradient step, our approach relies on simulating a batch of distributions from the current prior. Consequently, our procedure is easiest to implement when the prior is easy to sample from. Although it is not difficult to simulate from the mixture of a finite number of distributions used in earlier prior specifications (*4*, *5*), the number of support points needed in this mixture is large in many problems. In our implementations, at each step *k*, we parameterize the prior in the alternating approach as a generator network *G_k_* (*19*) that takes as input a source of randomness *U* and outputs a distribution *G_k_*(*U*) in our statistical model. Because the size of the neural network is fixed across iterations, the computational complexity of evaluating *G_k_*(*U*) does not increase in *k*. This comes at the cost of learning a Γ-minimax procedure (*24*), that is, a Bayes procedure under a least favorable prior within some restricted class Γ, rather than a traditional minimax procedure as defined earlier. However, neural networks are good approximations of rich function classes (*25*). Consequently, in practice, we expect the parameterization of the class of priors via a generator network to be unrestrictive.

Thus far, we have not discussed the role of the observed data in our learning scheme. In practice, a dataset *X* is observed, where these data are drawn from some distribution *P*. Our learning scheme does not make use of *X* until after a procedure *T* with low maximal risk has been trained—it is only at the end of this scheme that the learned procedure *T* is applied to the observed data *X*. An advantage of this kind of offline training of *T* is that, once *T* is learned, this procedure can be saved for use in future applications, thereby removing the need to learn it again. It follows that, although the upfront computational cost of learning a procedure *T* can be steep, this cost only needs to be paid once. Once trained, our learned procedures only require as much computation as is required to evaluate a neural network of the given size. In settings in which a procedure with low maximal risk can be expressed as a small neural network, evaluating our learned procedures can actually be faster than existing methods, which often require solving a dataset-dependent optimization scheme—this is the case, for example, in a clustering setting that we explore in our numerical experiments.

### Overview of numerical experiments

We report numerical illustrations of our approach for point estimation, prediction, and confidence region construction. Additional details are provided in Materials and Methods. We also report on key challenges we encountered while conducting our experiments.

In most of these experiments, we used alternating algorithms to learn (nearly) minimax statistical procedures. We chose to primarily rely on alternating algorithms because we found them to be more computationally efficient than their nested minimax and nested maximin counterparts. Nonetheless, in one of our examples, namely, an example in which we predict a binary outcome based on covariates, the alternating algorithms that we implemented struggled to learn a useful statistical procedure—we discuss this issue after the presentation of the experiments. For this reason, we used a nested minimax algorithm in that setting.

### Numerical experiments: Point estimation

Three sets of point estimation experiments were conducted. In our first set of experiments, we observe *n* independent variates drawn from a common Gaussian distribution, and our goal is to estimate its mean μ or standard deviation (SD) σ. The risk of a candidate estimator is quantified by its MSE. To construct an approximate minimax estimator of either μ or σ, we used alternating algorithms. We implemented these algorithms by parameterizing (i) generator networks as multilayer perceptrons that take Gaussian noise as input and return draws of (μ, σ) and (ii) procedures as multilayer perceptrons that take the entire dataset as input and return an estimate. Although in this problem the minimax estimators at most rely on the first and second empirical moments by sufficiency (*26*), our parameterization ignores this information to better imitate the lack of available statistical knowledge in more challenging problems. Of course, we could easily incorporate this knowledge in our framework.

When *n* = 1 and it is known that σ = 1 and μ ∈ [ −*m*, *m*] for some *m* > 0, our alternating algorithm produced an estimator of μ whose maximal risk is within 5% of the minimax risk across a variety of values of *m* (see table S1 for details). We also studied estimation of μ and σ separately when *n* = 50 and it is known that μ ∈ [ −5,5] and σ ∈ [1,4]. The maximal risk of the learned estimators of μ and σ was found to be lower than the maximal risk of the MLEs by 2.1% (0.313 versus 0.320) and 23.5% (0.086 versus 0.112), respectively. Movie S1 shows the evolution of the risk of the learned estimator of μ across the parameter space as the weights of the neural network are updated. Figure S1 shows the corresponding evolution in maximal risk.

**Fig. 2:**
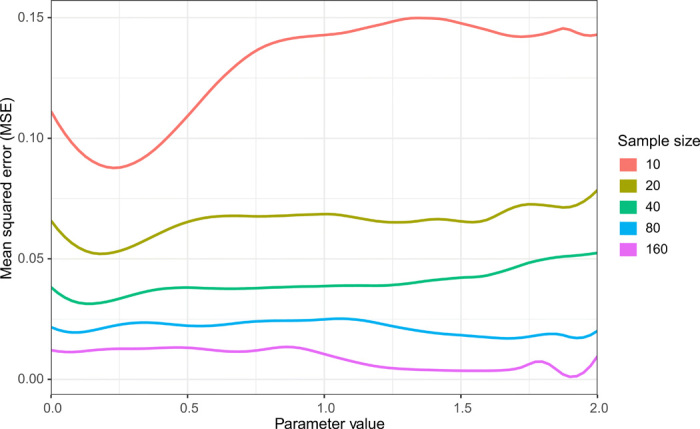
Risk of learned estimators in example where the MLE is inconsistent. Risks are displayed at different parameter values and sample sizes. Unlike for the MLE, for which the maximal risk increases with sample size, the maximal risk of our estimators decreases with sample size.

Our second set of point estimation experiments highlights that our automated approach can yield a useful procedure even when a standard analytical approach fails. We considered a setting where a sample of *n* independent variates is drawn from a two-component equal-probability mixture between a standard Gaussian distribution and the distribution of γ + *Z* exp ( −γ^−2^), with *Z* also a standard Gaussian variate. The goal is to estimate γ using the available sample. As before, we used the MSE risk. This problem is interesting because the MLE has been shown to be inconsistent (*27*). We verified numerically that the maximal risk of the MLE did not tend to zero—even worse, in the sample sizes that we considered (*n* ranging from 10 to 160), it increased from around 1.5 to over 3.5. In contrast, as shown in Fig. 2, the maximal risk of our learned estimators decreases with sample size.

In our final set of point estimation experiments, we evaluated the performance of our method for estimating the coefficients in two poorly conditioned linear regression problems. For a fixed *n* × 2 design matrix *w*, an outcome *Y* = *w*β + ϵ is observed, where β = (β_1_, β_2_) ∈ ℝ^2^ falls in a closed 𝓁_2_ ball centered at the origin with radius 10, and ε is a vector of *n* independent standard normal random variables. The objective is to estimate β_1_. We considered two design matrices for *w*. The first design matrix has 32 rows corresponding to engine displacement and number of cylinders across 32 automobiles (*28*), where the two columns of *w* have a correlation of approximately 0.9. The second dataset has eight rows of synthetic data (*29*), where the two columns of *w* have a correlation of approximately 0.994. We standardized the columns in both of these design matrices to have mean zero and variance one. The condition numbers of the *w*^⊤^*w* matrices in these two settings are approximately 20 and 350, respectively. Here, we recall that a regression problem is considered to have moderate or strong multicollinearities if this matrix has condition number of at least 30 or 100, respectively (*30*). We compared our method to OLS, where we solve the OLS problem subject to the constraint imposed by the model on the 𝓁_2_ norm of β. We also compared our procedure to a ridge regression estimator with tuning parameter selected by cross-validation (*31*). Because we found that OLS consistently outperforms ridge regression in these settings, we do not present ridge regression results here. We evaluated performance for estimating β_1_ in terms of maximal MSE for β in our parameter space and also in terms of Bayes MSE when a uniform prior is imposed on β.

Our learned procedure outperformed OLS in the setting in which the condition number of *w*^⊤^*w* was 350. Specifically, in this setting, the maximal MSE of our procedure was 23% lower than that of OLS (8.9 versus 11.6), and the Bayes MSE was 19% lower (6.8 versus 8.4). Our learned procedure was slightly outperformed by OLS in the setting in which the condition number of *w*^⊤^*w* was 20. Specifically, in this setting, the maximal MSE of our procedure was 8% higher than that of OLS (0.184 versus 0.171) and the Bayes MSE was less than 1% higher (0.168 versus 0.167). These results suggest that there may be more room for improvement over OLS in more poorly conditioned regression problems.

### Numerical experiments: Prediction

In our second set of experiments, we examined the use of AMC in generating individual-level predictions. We considered two classes of prediction problems. In the first, the goal is to predict the value of a binary outcome *Y* using observed covariates, whereas the second involves clustering a collection of observations drawn from a two-component Gaussian mixture model.

We start by presenting results for the binary prediction problems that we considered. We considered a setting in which *n* = 50 independent draws are obtained from the distribution of the observation unit *X* = (*W*, *Y*), where *W* ∈ ℝ*^p^* is a covariate vector and *Y* is a binary outcome. Our goal is to learn the conditional mean of *Y* given *W*, which can be used to predict *Y* from an observed value of *W*. Here, we measure prediction performance as the Kullback-Leibler divergence

$$R(T,P)=E_{P}[\int \text{log}\;\{\frac{E_{P}(Y|W=w)}{T(X)(w)}\}\mathrm{dQ}(w)]$$ (2)

First, we considered the statistical model to be the set containing each distribution *P* with known covariate distribution *Q* and with conditional mean function satisfying ϕ(*E_P_*(*Y*|*W* = *w*)) = α + β^⊤^*w* for some vector (α, β) ∈ ℝ × ℝ*^p^* and ϕ defined pointwise as ϕ(*t*) = logit{(*t* − 0.1)/0.8}. The distribution *Q* varies across the settings—in some of the settings, the predictors drawn from *Q* are independent, whereas in others they are correlated; in some of the settings, the predictors are all continuous, whereas in others there are both discrete and continuous predictors. The link function ϕ enforces the conditional mean function to have range in [0.1,0.9]. This avoids predictions falling close to zero or one, which could otherwise lead to instability caused by very large values of risk function gradients. We examined covariate dimension *p* ∈ {2,10}. All of the generalized linear models considered are fully identifiable, in the sense that each distribution in the model corresponds to exactly one choice of the indexing parameters (α, β). We then explored the performance of our method for two models that fail to satisfy this identifiability condition. Specifically, we considered the statistical model in which the covariate distribution is again known to be *Q* and the conditional mean outcome is a multilayer perceptron with one or two hidden layers, each containing three nodes, with a hyperbolic tangent activation function and output activation function ϕ.

The various settings we considered differed in parameter dimension, ranging from 3 to 25. In all our experiments, we considered several sets of bounds on these parameters. Details are provided in Table 1. The procedure class was taken to be an LSTM network that recursively takes observations as input and returns hidden states, which are subsequently linearly transformed to the dimension of the unknown parameters and passed through a mean pooling layer, as detailed in Materials and Methods.

**Table 1:** Settings for the prediction example. The parameterization of the models considered is described where the example in Results is introduced. Complexity identifies the relative size of the models in the multilayer perceptron settings i, ii, and iii, the 10-dimensional generalized linear model settings iv, v, and vi, and the 2-dimensional generalized linear model settings x, xi, and xii. “Gaussian” corresponds to *p* independent standard normal predictors. “Mixed” correspond to two independent predictors following standard normal and Rademacher distributions. The variable *h* is the number of hidden layers that the model uses for the *E*[*Y*|*W*] network; *b*_1_ is the bound on the magnitude of the bias in the output node of the network; *b*_2_ is a bound on all other biases and all network weights; ρ is the correlation between the predictors; *s*_1_, *s*_2_, and *s*_3_ are the number of distributions in the random search for an unfavorable distribution that are chosen uniformly from the entire parameter space, uniformly from the boundary, and a mixture of a uniform draw from the entire parameter space and from the boundary (details in the main text); and *t* is the number of starts used for the shallow interrogation.

**Settings**	**Complexity**	**Predictors**	***p***	***h***	***b*_1_**	***b*_2_**	**ρ**	***s*_1_**	***s*_2_**	***s*_3_**	***t***
i	Lowest	Gaussian	2	0	2	2	0	200	2	0	3
ii	Medium	Gaussian	2	1	2	2	0	150	50	50	5
iii	Highest	Gaussian	2	2	2	2	0	150	50	50	5
iv	Lowest	Gaussian	10	0	0	0.5	0	150	50	0	5
v	Medium	Gaussian	10	0	1	0.5	0	150	50	0	5
vi	Highest	Gaussian	10	0	2	0.5	0	150	50	0	5
vii	Lowest	Gaussian	10	0	0	0.5	0.3	150	50	0	5
viii	Medium	Gaussian	10	0	0	0.5	0.6	150	50	0	5
ix	Highest	Gaussian	10	0	0	0.5	0.9	150	50	0	5
x	Lowest	Mixed	2	0	1	0.5	0	200	2	0	3
xi	Medium	Mixed	2	0	1	1	0	200	2	0	3
xii	Highest	Mixed	2	0	1	2	0	200	2	0	3

Figure S2 shows examples of shapes the multilayer perceptron with two hidden layers can take. Our learned procedure was applied on each of 20,000 randomly generated datasets, and for each dataset, predictions were obtained on a fixed uniform grid of 400 *W*_1_ values in [ −2,2]. Pointwise quantiles of these predictions are displayed in blue. The overall shape of the conditional mean functions returned by our learned procedure agrees with the true function. However, these shapes are more discrepant for extreme values of *W*_1_.

**Fig. 3:**
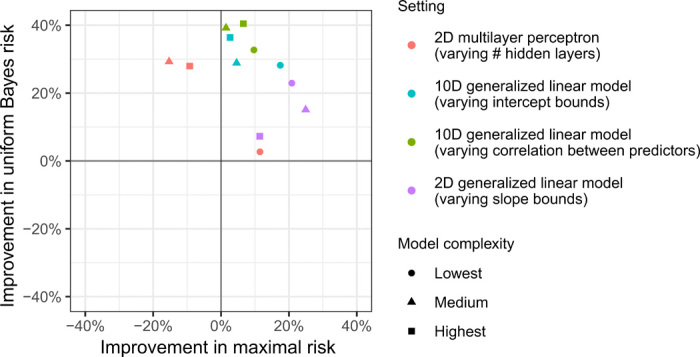
Performance of learned prediction algorithms. Percent improvement in maximal risk and Bayes risk of our learned prediction algorithms relative to MLEs in the models shown in Table 1. The Bayes risk conveys a method’s performance over the entire model, although it is least informative for the multilayer perceptron model (where this prior puts most of its mass on simpler functional forms, e.g., on the top left panel of fig. S2 rather than the bottom left panel).

Figure 3 shows the relative performance of our final learned procedures compared to the reference procedure, which was taken to be the MLE that optimizes over the same model. Performance metric values from our experiment can be found in table S2. In all settings except for the multilayer perceptrons with one and two hidden layers, our learned procedures outperformed the MLE in terms of maximal risk. Our methods also outperformed the MLE in terms of Bayes risk with respect to a uniform prior, suggesting superior performance at many distributions in the model, rather than only at the least favorable distribution. As fig. S3 shows, in many cases, our learned procedures outperformed the MLE in terms of uniform Bayes risk after very few iterations. In addition, because it is commonly used for prediction of binary outcomes, we compared our method to standard main-term logistic regression, also including a sparsity-inducing lasso penalty for experiments with *p* = 10 (*31*). Our procedures outperformed these competitors in all cases, as shown in table S2.

As highlighted earlier, a major advantage of using a nested minimax (or alternating) algorithm is that knowledge of existing statistical procedures can be incorporated in the meta-learning process. We describe as aware any procedure obtained through AMC with access to an existing procedure. We ran smaller-scale experiments to illustrate this feature. Specifically, in the two settings involving multilayer perceptrons, we learned procedures that take as input both the raw data and the OLS regression coefficients fitted using these data. We examined interrogations of the aware and unaware approaches obtained after 15, 16, …, 30 thousand iterations. For procedures based on a perceptron with one hidden layer, the largest and average maximal risks across iterations were both smaller by 6% for aware versus unaware prediction procedures. When two hidden layers were used, the aware procedure outperformed its unaware counterpart by 10% in terms of largest maximal risk but was outperformed by 1% in terms of average maximal risk. These results highlight the potential promise of aware procedures, especially in light of the fact that, in these experiments, awareness was built around an overly parsimonious statistical procedure.

We now present results for the clustering problem that we considered. In this experiment, *n* = 10 independent observations are drawn from a mixture of a Normal(μ_1_,1) distribution and a Normal(μ_2_,1) distribution, where the mixture weight is ω. The model enforces that both μ_1_ and μ_2_ fall in the interval [−3, 3]. The objective is to partition the 10 observations into two sets according to which mixture component they were generated from. Rather than explicitly partition the data, we let *T*(*X*) = (*T*(*X*)_1_, …, *T*(*X*)*_n_*) denote a vector of membership probabilities for the *n* observations, that is, a vector of probabilities that an observation was drawn from a given component. We want observations with high membership probabilities to all be drawn from the same component of the mixture and observations with low membership probabilities to all be drawn from the other component. We used the following risk function during training

$$R(T,P)={\left(\mu_{1}-\mu_{2}\right)}^{2}E_{(X,C)\sim P}[\underset{j\in \{1,2\}}{\text{min}}\;\frac{1}{n}\sum_{i=1}^{n}{\left(1\{C_{i}=j\}T{\left(X\right)}_{i}+1\{C_{i}\neq j\}[1-T{\left(X\right)}_{i}]\right)}^{2}]$$ (3)

Above, *C_i_* denotes the mixture component from which the *i*th observation was actually drawn—although *C* = (*C*_1_, …, *C_n_*) was drawn from *P*, the procedure does not actually have access to these component indicators. We note that the (μ_1_ − μ_2_)^2^ term in the risk above downweights the risk when this classification problem is difficult, namely, when the two mixture components are similar. We used an alternating algorithm to train our procedure, where the prior in this algorithm drew the mixture weight ω uniformly from the unit interval and adversarially selected (μ_1_, μ_2_) to maximize the risk in (*3*). This is equivalent to using the above risk in a hierarchical model in which ω is treated as a standard uniform random variable. After training our procedure, we also evaluated its performance with respect to misclassification error, where this risk function is defined as

$$\left(T,P)\mapsto E_{(X,C)\sim P}[\underset{j\in \{1,2\}}{\text{min}}\;\frac{1}{n}\sum_{i=1}^{n}(1\{C_{i}=j\}I\{T{\left(X\right)}_{i}>0.5\}+1\{C_{i}\neq j\}I\{T{\left(X\right)}_{i}\leq 0.5\})\right]$$ (4)

We report worst-case performance with respect to these risks in the hierarchical setting in which ω is a uniform random variable and in a nonhierarchical setting in which ω is selected adversarially. We also evaluated the uniform Bayes risk of these methods, that is, the Bayes risk for which (ω, μ_1_, μ_2_) are drawn uniformly from the parameter space. We compared the performance of our learned procedure to that of the *k*-means clustering algorithm (*k* = 2) and also to that of the expectation-maximization algorithm (EM) that aims to maximize the likelihood over a two-component Gaussian mixture model in which the variances of the two mixture components are equal to one, (μ_1_, μ_2_) ∈ ℝ^2^, and ω is fixed and unknown. Hereafter, when we refer to EM, we are referring specifically to the EM optimization scheme as implemented in (*32*) for maximizing the likelihood over this model. Because EM performed similarly to or outperformed *k*-means for nearly all reported metrics, we do not report numerical risk summaries for this procedure. When evaluating misclassification risk for EM, we define the two clusters by partitioning the two classes at 0.5.

**Fig. 4:**
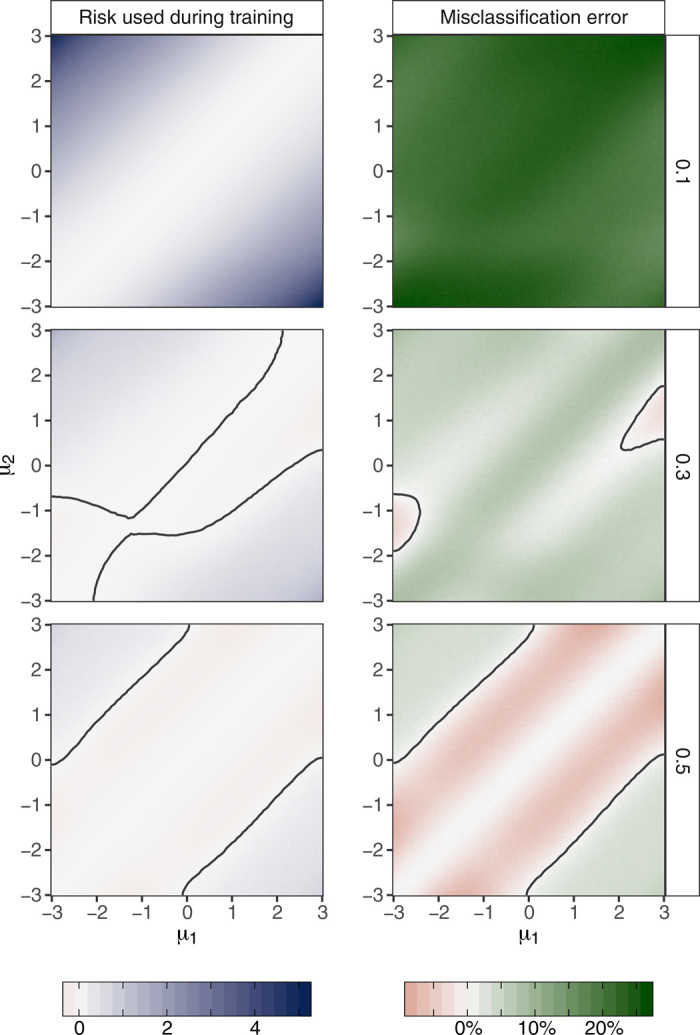
Performance of learned clustering procedure compared to performance of the EM. The difference between the risk of EM and our learned procedure is displayed—larger values indicate that our learned procedure outperformed EM. Three fixed values of the mixture weight ω are considered: 0.1,0.3, and 0.5. Contours indicate comparable performance of our learned clustering procedure and EM. Contours are drawn using smoothed estimates of the difference of the risks of the two procedures, where the smoothing is performed using *k*-nearest neighbors (*k* = 25). Our learned procedure outperformed EM both in terms of the risk in (*3*) that was used during training, and in terms of misclassification error.

In terms of the risk given in Eq. 3, our learned procedure outperformed EM by 86% (1.3 versus 9.6) in terms of worst-case risk in the nonhierarchical setting, by 81% (0.69 versus 3.6) in terms of worst-case risk in the hierarchical setting, and by 52% (0.39 versus 0.82) in terms of Bayes risk. In terms of misclassification error, our learned procedure outperformed EM by 5% (0.37 versus 0.39) in terms of worst-case risk in the nonhierarchical setting, by 29% (0.27 versus 0.38) in terms of worst-case risk in the hierarchical setting, and by 37% (0.17 versus 0.27) in terms of Bayes risk. We also compared the worst-case risk of our procedure to that of EM and *k*-means when ω was fixed to be equal to 0,0.1, …,0.5. We found that our procedure outperformed both of these procedures in terms of worst-case risk for all settings considered. When ω was fixed at zero, we found that our procedure markedly outperformed both of these alternative procedures in terms of the risk in (*3*)—this appears to have occurred because our learned procedures were able to adaptively determine that there was only one observed cluster in this setting. Figure 4 shows the strong performance of our learned method relative to EM at the fixed values ω = 0.1,0.3, and 0.5.

In both the binary prediction and the clustering settings, we compared the runtime of our learned procedure to that of existing methods. Although we trained our procedures on graphics processing units (GPUs), here, we ran all methods on a central processing unit (CPU). For a two-dimensional generalized linear model, we found that it took approximately 40 times as long to evaluate our procedure on a dataset as to run a generalized linear model in Julia. This increase in runtime likely resulted from the use of an LSTM network to parameterize our procedure, which requires iterating through the *n* observations, each time multiplying and adding large matrices together. Because we used a nested minimax algorithm to train our binary prediction procedures, these procedures also required a substantial amount of training time—specifically, they required approximately 1 week of training. Nonetheless, we note that if worst-case risk is of primary concern, then our learned procedures may be preferred, as they outperformed all comparators in this metric.

We also compared the runtime of our learned clustering procedure to the EM and *k*-means implementations that we used, both of which were based on Julia code from publicly available repositories—links to these repositories can be found in our source code. On 10,000 randomly generated datasets, our learned procedure, on average, evaluated approximately 10 times faster than *k*-means and 400 times faster than EM. This improved runtime came at the cost of an initial offline training time of 6 hours on a GPU cloud computing service.

We conclude by cautioning the reader that it can be misleading to numerically evaluate the runtime of algorithms, because it may be possible to produce faster implementations of our algorithm or its comparators. Therefore, the runtime comparisons that we have presented are only valid for the particular prediction procedure implementations that we considered.

### Numerical experiments: Confidence region construction

As in our point estimation illustration, we consider an experiment in which *n* independent draws from a Gaussian distribution with mean μ and SD σ are observed. The goal is now to develop a joint confidence region for μ and σ of the form {(μ, σ) : μ ∈ [μ*_l_*, μ*_u_*], σ ∈ [σ*_l_*, σ*_u_*]} and with coverage probability at least 95%. The statistical model we consider imposes that μϵ[−10, 10] and σϵ[−10, 10]. The network architecture that maps the data into the vector (μ*_l_*, μ*_u_*, σ*_l_*, σ*_u_*) indexing candidate regions is more complex than in experiments discussed so far. Details are provided in Supplementary Appendix C. We evaluated candidate confidence region procedures using two criteria: coverage—the probability that the region contains (μ, σ) for a new random sample—and expected (information-normalized) size—the expected value of (μ*_u_* − μ*_l_*)(σ*_u_* − σ*_l_*)/σ^2^. As a comparator for the procedure learned through AMC, we considered a random rectangular region based on the sample mean and SD estimates (see Supplementary Appendix C for details). We found this comparator procedure to have 95% coverage probability and approximate expected size 0.315 throughout the statistical model. Therefore, when reporting results, we standardize all expected sizes by 0.315 so that the reference procedure has expected size 1.

The output of our learning algorithm is a class of confidence region procedures indexed by a tuning parameter η that controls the trade-off between coverage and expected size. Lower values of η correspond to larger confidence regions with higher coverage. Figure S4 displays the coverage and area of the learned procedure for three choices of η. We evaluated the coverage of these procedures on a uniform partition of the (μ, σ) parameter space of size 500 × 500. For moderately sized η, coverage of at least 95% was achieved on 70% of the partition. We found the procedure to have a worst-case coverage of 90% and a worst-case size of 1.27. For smaller η, we found a worst-case coverage of 93% and a worst-case size of 1.53, while achieving 95% coverage for 89% of the (μ, σ) partition. For larger η, we found a worst-case coverage of 88% and a worst-case size of 1.15 while achieving 95% coverage for 50% of the (μ, σ) in our grid. While the reference procedure did outperform the learned procedure in this problem, our findings highlight that there is promise in the use of AMC for automating the construction of confidence region using neural networks, possibly borrowing ideas from (*9*, *10*). Last, we note that although our framework easily allows for principled, automated selection of η via interrogation—for example, choosing the largest η value that attains proper worst-case coverage—we did not pursue this extension.

### Challenges in numerical experiments

Several challenges arose in the course of experiments. First, in our initial implementations of the alternating algorithm, the support of the prior often collapsed on a single distribution, leading to poor performance of the learned procedure at all other distributions. This is analogous to “mode collapse” for GANs (*33*). In many cases, we found that this problem could be overcome by choosing a particular fixed prior—for example, the initial or uniform prior—and by penalizing the prior network’s objective function when the current procedure had lower risk under the current versus fixed prior. This is justified by the fact that the algorithm for updating the current prior strives to yield a least favorable prior, implying that eventually the current prior should not be more favorable than the fixed prior. In some settings, namely, in binary prediction and in developing the boundaries of the confidence region, this penalization was not sufficient to avoid mode collapse. For the binary prediction problem, we instead used the nested minimax approach. For the boundary of the confidence region, we updated our procedure network against a fixed diffuse prior. This corresponds to using our AMC approach in a nonadversarial fashion, namely, to learn a Bayes procedure under this fixed prior. Although not directly used to define region boundaries, AMC was used when developing our confidence region procedure. Specifically, we used the alternating algorithm to learn the interior point around which the region was constructed (details appear in Supplementary Appendix C). In this case, we avoided mode collapse by both using the penalty described above and providing as additional inputs to the prior network estimates of the current procedure’s performance at six a priori specified distributions.

Second, approximating the maximal risk of our binary prediction procedures was difficult in many of our models due to the dimensionality of the indexing parameters. In an effort to accurately report the (nearly) worst-case performance of our learned procedures, we interrogated our learned procedures using a total of 300 runs of two optimization procedures. Because the MLE was substantially more computationally expensive to evaluate than our learned procedures, we only ran three runs of one optimization procedure for the prediction MLE. For the multilayer perceptron procedures, we also evaluated the risk of the MLE at the most unfavorable distribution identified for our learned procedures.

Third, when we first trained our confidence region procedure, it performed poorly for σ near the lower boundary of the statistical model consisting of all μϵ[−10, 10] and σϵ[1, 10]. To help overcome this challenge, we instead trained our procedure over the expanded model in which μ satisfies the same bounds but σϵ[0.5, 10]. Training over this slightly larger model substantially improved performance for σϵ[1, 10]. In future work, we plan to evaluate whether expanding the statistical model during training will generally help to alleviate poor boundary behavior in other problems.

### Performance in data applications

To illustrate the practical performance of procedures obtained through AMC, we used our learned prediction procedures to predict survival on the Titanic using either a 2- or 10-dimensional passenger covariate vector (*34*) and to predict the CD4^+^ T cell immune response induced by the PENNVAX-B DNA HIV vaccine in the phase 1 HIV Vaccine Trials Network (HVTN) 070 trial using sex and body mass index (BMI) (*35*). The Titanic dataset has been used to benchmark binary prediction procedures (*36*), whereas the extent to which sex and BMI predict CD4^+^ response to the PENNVAX-B vaccine is of independent scientific interest (*37*). In all cases, we compared our learned prediction procedure to (i) MLEs within the same models used to train our procedures, (ii) main-term logistic regression, and (iii) the lasso estimator with cross-validated tuning (*31*). Details on the models used in each data application are provided in Materials and Methods. We evaluated performance using cross-validated cross-entropy risk and area under the receiver operating characteristic curve (AUC), where, for both the Titanic analyses and the HIV vaccine analyses, training sets of 50 observations are used.

**Fig. 5:**
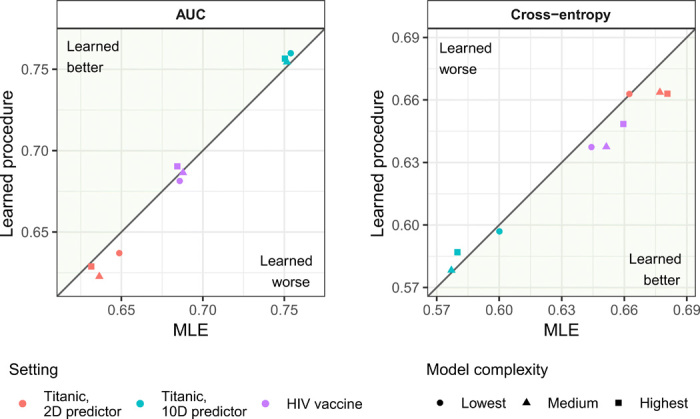
Cross-validated performance of learned prediction algorithms and MLEs. Models of three different complexities are considered when training the learned prediction algorithms for each application (see Materials and Methods for details). MLEs are evaluated over the same models that were used to train the learned prediction algorithms.

Figure 5 gives an overview of the results of our analyses. Exact performance metrics and the corresponding CIs can be found in table S3. In the HIV vaccine analysis, our three learned procedures yielded an AUC value of 68.1% (95% CI, 54.4 to 81.9%), 68.6% (95% CI, 55.0 to 82.2%), and 69.0% (95% CI, 55.5 to 82.6%), suggesting that sex and BMI are predictive of CD4^+^ immune responses to the PENNVAX-B HIV vaccine. In all cases, our method performed similarly to the MLE in terms of both cross-entropy and AUC. Because we assumed different models than those upon which the logistic regression and lasso estimators build, the performance assessments are not directly comparable across methods. Nonetheless, in most settings, the results were similar. A notable exception occurred when predicting survival on the Titanic using 10 passenger variables. In this case, lasso slightly outperformed our learned algorithms, in terms of both AUC (78.2% versus 75.0 to 75.4%) and cross-entropy (0.560 versus 0.577 to 0.600). We hypothesize that we could obtain similar performance using these 10 covariates if we were to assume a lasso constraint on the regression coefficients in our model. Briefly, the numerical results we obtained provide preliminary evidence that our learned procedures generally perform at least as well as existing methods on real datasets that were not generated adversarially.

### Theoretical guarantees

Under some conditions, it is possible to formally establish theoretical guarantees on the risk of a learned procedure and on its rate of convergence across iterations. Here, we consider the class T of statistical procedures to be unconstrained except for minor regularity conditions. We begin by assuming that the risk is convex as a function of the statistical procedure. We outline a subgradient algorithm for iteratively updating a statistical procedure to improve its maximal risk and then present a theorem guaranteeing that the maximal risk of the procedure resulting from this algorithm converges to the minimax risk as the number of iterations increases. In particular, the discrepancy between the risk of the resulting procedure and the minimax risk is of order *K*^−1/2^, where *K* is the number of iterations performed. In Supplementary Appendix E, we also show that, when the risk is not convex, the maximal risk of the resulting procedures converges to a local minimum.

We consider the setting in which the parameter space is a Hilbert space H of functions mapping from some set A to the real line ℝ, equipped with inner product 〈 ·, · 〉_ℋ_. If the parameter space is the real line or a subset thereof, then A is simply the empty set. If the objective is to predict an outcome using a *p*-dimensional predictor, then H may be taken to be the class of ξ-square integrable real-valued functions defined on ℝ*^p^* for some dominating measure ξ. Let ν denote a σ-finite measure on the support X of *X* that dominates all distributions in the statistical model P and suppose that T contains each function T:X→H for which *S* : (*x*, *a*) ↦ *T*(*x*)(*a*) satisfies ∫〈*S*(*x*, ·), *S*(*x*, ·)〉_ℋ_*dν*(*x*) < ∞. We denote by S the Hilbert space containing each corresponding function *S* obtained from some T∈T, equipped with inner product

$$\langle S,\tilde{S}\rangle =\int {\langle S(x,\cdot ),\tilde{S}(x,\cdot )\rangle }_{H}d\nu (x),S,\tilde{S}\in S$$ (5)

Because each *S* corresponds to a unique T∈T, we will sometimes write R(*S*, *P*) to mean R(*T*, *P*).

Below, we will require several conditions. The first condition involves differentiability of the risk functional *S* ↦ R(*S*, *P*) at *P*:

A1) For each distribution P∈P and procedure *S*, the risk functional at *P* is Gâteaux differentiable at *S*, in the sense that $\delta_{h}R(S,P)={\frac{d}{d\zeta }R(S+\zeta h,P)\mathrm{ht}|}_{\zeta =0}$ exists for each h∈S and, furthermore, *h* ↦ δ*_h_*R(*S*, *P*) is bounded and linear over S.

Under condition A1, the Riesz representation theorem implies that there exists a gradient g(S,P)∈S such that δ*_h_*R(*S*, *P*) may be written as 〈*g*(*S*, *P*), *h*〉 for each h∈S. In Result 1 in Materials and Methods, we provide an expression for this gradient whenever the risk is the expectation of a loss function. Defining the maximal risk functional $R^{⋆}$ as the map $S\mapsto {\text{sup}}_{P\in P}R(S,P)$, we show in Lemma 2 in Materials and Methods that *g*(*S*, *P*) is an approximate subgradient of $R^{⋆}$ at *S* if *P* is unfavorable and the following convexity condition holds:

A2) For each P∈P, the risk functional at *P* is convex on S, in the sense that the inequality $R((1-t)S+t\tilde{S},P)\leq (1-t)R(S,P)+tR(\tilde{S},P)$ is true for all *t* ∈ [0,1] and each $S,\tilde{S}\in S$.

For brevity, we will refer to any “approximate subgradient” simply as a “subgradient.” The upcoming theorem properly accounts for the fact that we only require these subgradients to be approximate in the sense of Lemma 2.

At each step *k*, we calculate a subgradient by interrogating the current procedure *S_k_* to identify an unfavorable distribution *P_k_*. Given access to the corresponding subgradient *g_k_* = *g*(*S_k_*, *P_k_*), we could aim to decrease the maximal risk of the current procedure by taking a step away from *g_k_*. In practice, computing the subgradient may be computationally expensive, and a stochastic subgradient descent algorithm may be preferred. For this reason, our theoretical results allow the use of a stochastic subgradient descent algorithm with access to an unbiased stochastic subgradient ${\hat{g}}_{k}=\hat{g}(S_{k},P_{k})$ drawn from a distribution *Q_k_* independently of the stochastic subgradients drawn at all earlier steps. We require that *Q_k_* = *Q*(*S_k_*, *P_k_*) for some fixed mapping *Q* that does not depend on *k* and that ${\hat{g}}_{k}$ is an unbiased estimator of *g_k_*, in the sense that ${\hat{g}}_{k}(x,a)$ has mean *g_k_*(*x*, *a*) under *Q_k_* for all (*x*, *a*). Our proposed stochastic subgradient algorithm is defined by the update

$$S_{k+1}:(x,a)\mapsto S_{k}(x,a)-\zeta_{k}{\hat{g}}_{k}(x,a)$$ (6)

where ζ*_k_* is the step size.

We now study the convergence of this algorithm. We establish that, if the risk functional is convex, the risk of the learned statistical procedure converges to the minimax risk as sample size grows, both in expectation and in probability. The next theorem requires the following conditions:

A3) The set $S^{⋆}=\{S\in S:R^{⋆}(S)={\text{inf}}_{\tilde{S}\in S}R^{⋆}(\tilde{S})\}$ of minimax procedures is nonempty.

A4) The subgradient has bounded magnitude $M={\text{sup}}_{S\in S,P\in P}E_{Q(S,P)}{\left\|\hat{g}(S,P)\right\|}^{2}<\infty$.

A5) The distance $\rho (S_{1},S^{⋆})={\text{inf}}_{S\in S^{⋆}}\|S_{1}-S\|$ between the initial procedure *S*_1_ and the set of minimax optimal procedures is finite.

We denote by ϵ*_k_* = *E*[$R^{⋆}$(*S_k_*) − R(*S_k_*, *P_k_*)] the extent to which the interrogation algorithm at step *k* is expected to underperform when attempting to identify the least favorable distribution, where the expectation is over the randomness in the stochastic subgradient estimates. If ϵ*_k_* is small, then there is greater expectation that the interrogation algorithm will find an unfavorable distribution at iteration *k*. The convergence result in the theorem requires the following condition on the step size ζ*_k_* and on the suboptimality measure ϵ*_k_* of the interrogation:

A6) As *K* tends to infinity, $\sum_{k=1}^{K}\zeta_{k}$ tends to infinity and max {ζ*_K_*, ϵ*_K_*} tends to zero.

The theorem we now present clarifies the manner in which step size and suboptimality measure possibly affect the magnitude of the deviation $\varepsilon_{K}={\text{min}}_{k=1,2,\ldots ,K}E[R^{⋆}(S_{k})]-{\text{inf}}_{S\in S}R^{⋆}(S)$ of the best expected risk up to step *k* from the minimax risk.

**Theorem 1** (Convergence to minimax optimal procedure for convex risks). Fix an initial procedure *S*_1_ and define *S_k_* recursively according to Eq. 6. If conditions A1 to A5 hold, then, at any step *K*

$$\varepsilon_{K}\leq \frac{\rho {\left(S_{1},S^{⋆}\right)}^{2}+\sum_{k=1}^{K}\zeta_{k}(M\zeta_{k}+2\epsilon_{k})}{2\sum_{k=1}^{K}\zeta_{k}}$$ (7)

If, additionally, condition A6 holds, then ε*_K_* converges to zero as *K* tends to infinity.

The proof of Theorem 1 is given in Materials and Methods and makes use of arguments given in (*38*).

Whenever ζ*_k_* is proportional to *k*^−1/2^ and ϵ*_k_* is of order *k*^−1/2^, the right-hand side of Eq. 7 is of order *K*^−1/2^ log *K*. If the interrogation only achieves a fixed level of precision ϵ that does not diminish over iterations, then ε_*K*_ is instead bounded above by ϵ up to a term of order *K*^−1/2^ log *K*.

Because min_*k*=1,2,…,*K*_*E*[$R^{⋆}$(*S_k_*)] ≥ *E*[min_*k*=1,2,…,*K*_$R^{⋆}$(*S_k_*)], Theorem 1 gives conditions under which we have convergence guarantees for the expected best performance along the sequence of procedures returned by the gradient descent algorithm. By a simple application of Markov’s inequality, this also gives probabilistic guarantees. If the investigator keeps track of the index of the procedure with lowest maximal risk up to each iteration number, then this corresponds to reporting the procedure with lowest maximal risk upon the algorithm’s termination.

Conditions A4 and A5 place restrictions on the dominating measure ν. Condition A5 is most plausible when the measure ν is finite. If ν is not finite, it may be possible to find an initial procedure *S*_1_ that is very similar to a minimax estimator, in the sense that

$$\begin{array}{c} \text{there exists}\;a\;\delta >0\;\text{and}\\ S^{⋆}\in S^{⋆}\text{such that}\;\underset{x\in X}{\text{sup}}|S_{1}(x)-S^{⋆}(x)|<\delta \end{array}$$ (8)

and yet have that $\rho (S_{1},S^{⋆})=+\infty$. A benefit of choosing ν to be a finite measure is that condition A5 is guaranteed to hold if *S*_1_ satisfies Eq. 8. If the objective is to estimate a real-valued summary of *P* that belongs to a bounded subset *B* of ℝ, then, for all commonly used loss functions, *S*^⋆^(*x*) will fall in *B* for all x∈X and $S^{⋆}\in S^{⋆}$. Hence, Eq. 8 is satisfied if ν is a finite measure and *S*_1_ is chosen so that its range is contained in *B*. A similar argument can be used to demonstrate the desirable consequences of choosing ν to be a finite measure if the objective is to estimate a function-valued summary of *P*—for example, a regression function—whose range is bounded. Hence, it appears advantageous to choose ν to be finite.

We also give a result in Materials and Methods, suggesting that condition A4 is most plausible when the magnitude of $\frac{\mathrm{dP}}{d\nu }(x)$ is uniformly bounded over P∈P and x∈X. For both of these conditions to be satisfied, we generally require that the statistical model not be too large. In parametric models, this is similar to the assumption that the parameter space is compact—this assumption was made in (*4*, *5*). In Supplementary Appendix E.2, we suggest an appealing choice of a dominating measure ν. We show that, for an important class of statistical models, this measure is finite, dominates all distributions in P, and satisfies ${\text{sup}}_{P\in P,x\in X}\frac{\mathrm{dP}}{d\nu }(x)<+\infty$.

The constraint that H is a Hilbert space can be somewhat restrictive. Nevertheless, adaptations of our algorithm are possible to tackle cases where this constraint fails. For example, the collection of densities with respect to some dominating measure is not a linear space because densities cannot be negative and, therefore, it does not form a Hilbert space H. The collection of log densities is also not a linear space because densities must integrate to one. However, if density estimation is of interest, then the subgradient descent algorithm presented in this section can be modified to project each gradient step back onto the set of log densities. Proofs of convergence for projected subgradient descent are similar to the proof of Theorem 1 and are therefore omitted.

## DISCUSSION

Our AMC framework leverages recent developments in deep adversarial learning to construct minimax optimal statistical procedures. In numerical experiments and data analyses, we showed that our proposed approach yields procedures that are competitive with and can sometimes outperform those often used in practice.

We conclude by noting two limitations of our proposed approach—we also refer the reader to our earlier discussion of practical challenges that arose when implementing our approach in experiments. The first limitation is that it is not yet clear how it may be implemented to learn minimax procedures in semi- or nonparametric models, that is, in models that place few restrictions on how the data were generated. Although the theoretical guarantees that we have provided apply to this case, as a first step forward, our current experiments exclusively focused on parametric models, which are easier to explore using our interrogation strategies and to simulate from. In future work, we hope to provide practical suggestions for learning minimax procedures in these larger models. One possible strategy would be to use a parametric model that is rich enough so that it closely approximates a larger model of interest. The second limitation of our proposed approach is that running the algorithms used to learn and interrogate our procedures required a large amount of computing time. In particular, over 10,000 computing hours on state-of-the-art GPUs were needed to generate our results. During this time, our iterative learning strategy and interrogation strategies evaluated the candidate and final learned procedures on many simulated datasets—in some of our experiments, these procedures were evaluated nearly 10 billion times. Despite this high computational burden, as the cost of computing continues to decrease, it may prove more cost-effective to construct new statistical procedures using our proposed strategy along with large amounts of computing power rather than using complex analytic calculations. This work describes a technique for constructing optimal statistical procedures using deep adversarial learning. Nevertheless, further work is needed to evaluate the performance of our proposal in larger-scale problems that arise in scientific practice, especially those where existing statistical procedures fail.

## MATERIALS AND METHODS

### Pseudocode for algorithms for constructing optimal statistical procedures

In this section, we present pseudocode for nested minimax, nested maximin, and alternating algorithms for constructing optimal statistical procedures. For clarity, we focus on the case that the class of statistical procedures and the prior generator network are both indexed by finite-dimensional vectors—this is the case, for example, if neural network classes are considered for the procedure and the generator. We denote these classes by {*T_t_* : *t* ∈ ℝ^*d*_1_^} and {*G_g_* : *g* ∈ ℝ^*d*_2_^}. Here, we focus on the special case that R(*T*, *P*) = E*_P_*[*L*(*T*(*X*), *P*)] for some loss function *L*. In the upcoming pseudocode, we obtain unbiased stochastic gradient estimates when needed by approximating expectations over functions of *P* drawn from Π using a single draw of *P* ~ Π and expectations over functions of *X* drawn from *P* with a single draw of *X* ~ *P*. In practice, the gradient can be computed for *m* independent draws of *P* and Π, and the resulting stochastic gradients can be averaged—in each of our numerical experiments, we approximated these expectations with several hundred such draws.

All of the described algorithms make use of gradient-based optimization methods. In our numerical experiments, all of our procedures and prior generator networks were parameterized as neural networks, and these gradients were computed using backpropagation.

We present pseudocode for nested minimax algorithms in Algorithm 1. A key step of these algorithms involves identifying an unfavorable distribution for a given statistical procedure *T*. This is accomplished by solving an optimization problem in which the objective is to maximize R(*T*, *P*) as a function of (the parameters indexing) *P*. The choice of optimization routine used in this step should depend on the characteristics of the statistical decision problem at hand. As *P* ↦ R(*T*, *P*) will generally be nonconcave, we expect that maximizing this function will be challenging in many problems—nonetheless, we note that our theoretical results suggest that identifying a near-maximizer of *P* ↦ R(*T*, *P*) will suffice to learn a procedure with nearly optimal maximal risk.

Algorithm 1. Pseudocode for nested minimax algorithms.

1: **initialize** an indexing parameter *t*(1) for the procedure.

2: **for** *k* = 1 to *K–1* **do** ⊳Iteratively update the procedure.

3: **initialize** a guess *P_k_* of unfavorable distribution for *T*_*t*(*k*)_.

4: **while** *P_k_* is not unfavorable, that is, while R(*T*_*t*(*k*)_, *P_k_*) ≪ ${\text{max}}_{P\epsilon P}\;R$(*T*_*t*(*k*)_, *P*) **do**

5: Update the distribution *P_k_* so that it is less favorable for *T*_*t*(*k*)_.

⊳For example, using gradient-based methods, genetic algorithms, or random search.

6: **end while**

7: Sample *X* ~ *P_k_*.

8: Update the parameter *t*(*k*) indexing the procedure by moving in the opposite direction of ∇_*t*(*k*)_*L*(*T*_*t*(*k*)_(*X*), *P_k_*). For example, *t*(*k*) could be updated via stochastic gradient descent

$$t(k+1)=t(k)-\frac{1}{\sqrt{k}}\nabla_{t(k)}L(T_{t(k)}(X),P_{k})$$ (9)

9: **end for**

10: **return** the procedure *T*_*t*(*K*)_.

We present pseudocode for existing maximin algorithms in Algorithm 2. We note that Nelson (*4*) presented a more general form of this algorithm, which differs from our displayed algorithm at line 8. Specifically, this more general algorithm first identifies a less favorable prior under which the Bayes risk of *T*_Π*_k_*_ is larger than the Bayes risk of *T*_Π*_k_*_ under Π*_k_* and then replaces Π*_k_* by a mixture that returns a draw from Π*_k_* with probability 1 − ϵ and returns a draw from this less favorable prior with probability ϵ. Note that a point mass at *P_k_* is an example of a prior that is less favorable than Π*_k_*. Kempthorne’s nested maximin algorithm differs from our formulation in line 9 by fitting a richer optimization problem at each iteration *k* that allows the number of points in the support of Π_*k* + 1_ to be the equal to or even less than that of Π*_k_*—details can be found in section 3 of (*5*).

We present pseudocode for alternating algorithms in Algorithm 3. These algorithms require the user to specify, in advance, (fixed) noise distributions *P_U_* and *P_Z_* from which it is easy to sample. The algorithm assumes access to a generator *G_g_*, with indexing parameter *g*, that takes as input noise *U* ~ *P_U_* and outputs a distribution in the model. In our experiments, *G_g_* is parameterized as a neural network so that the collection of priors implied by {*G_g_* : *g*} is rich. This algorithm also assumes, for a given distribution *P* in the model,

Algorithm 2. Pseudocode for existing nested maximin algorithms (*4*).

**Require:** A procedure FindBayes for finding the Bayes procedure *T*_Π_ under a given prior Π. In general, this procedure will require an optimization routine or the procedure *T*_Π_ will be approximated pointwise via Markov Chain Monte Carlo methods, although in some cases *T*_Π_ is available in closed form.

1: **initialize** a prior Π_1_.

2: **for** *k* = 1 to *K–1* **do** ⊳Iteratively update the prior.

3: *T*_Π*_k_*_ = FindBayes(Π*_k_*).

4: **initialize** a guess *P_k_* of unfavorable distribution for *T*_Π*_k_*_.

5: **while** *P_k_* is not unfavorable, that is, while $R(T_{\Pi_{k}},P_{k})\ll {\text{max}}_{P\in P}R(T_{\Pi_{k}},P)$ **do**

6: Update the distribution *P_k_* so that it is less favorable for *T*_Π*_k_*_.

⊳For example, using gradient-based methods, genetic algorithms, or random search.

7: **end while**

8: For each ϵ ∈ [0, 1], let Π_*k*,ϵ_ be a mixture that returns a draw from Π*_k_* with probability 1 − ϵ and returns *P_k_* with probability ϵ.

9: Let ϵ(*k*) be a maximizer of R(*T*_Π_*k*,ϵ__, Π_*k*,ϵ_) over ϵ ∈ [0,1], where *T*_Π_*k*,ϵ__ = FindBayes(Π_*k*,ϵ_).

10: Let Π_*k*+1_ = Π_*k*,ϵ(*k*)_.

11: **end for**

12: **return** the procedure FindBayes(Π*_K_*).

that the user has access to a generator *D_P_* that takes as input noise *Z* ~ *P_Z_* and outputs a sample *X* with distribution *P*. For a univariate distribution, where *P_U_* is taken to be a standard uniform distribution, an example of such a generator is the inverse cumulative distribution function (CDF) under *P*. For multivariate distributions, the generator can be indexed by a copula function and the CDFs of the marginal distributions under *P*. For location-scale families, such as the family of Gaussian distributions, the generator can be taken to be the function *z* ↦ μ + σ*z*, where μ and σ index the distribution *P*—we used this generator whenever we simulated from Gaussian distributions in our experiments.

We note that the update to the generator in line 6 of Algorithm 3 assumes that

$$g\mapsto L(T_{t(k)}(D_{G_{g}(U)}(Z)),G_{g}(U))$$

is differentiable at *g*(*k*), which can be assured to hold if the loss *L* is differentiable in both of its arguments, *g* ↦ *G_g_*(*u*) is differentiable for all *u*, and *g* ↦ *D*_*G_g_*(*u*)_(*z*) is differentiable for all *u*, *z*. Note that we should not expect *g* ↦ *D*_*G_g_*(*u*)_(*z*) to be everywhere differentiable if the support of *X* is discrete—consequently, this described algorithm will not apply in those settings. This problem can be avoided by modifying the algorithm to instead use the likelihood ratio method to obtain an unbiased gradient estimate [see (*39*)].

We used this likelihood ratio method when we implemented nested maximin approaches in the binary prediction example. Although the likelihood ratio method did give us an unbiased estimate of the gradient in that case, we still chose to use a nested minimax algorithm to learn our final procedures in that setting, namely, due to a problem where the support of the prior collapsed on an undesirable equilibrium in that setting.

Algorithm 3. Pseudocode for alternating algorithms.

1: **initialize** indexing parameters *t*(*1*) and *g*(*1*) for the procedure and the prior generator network, respectively. For an indexing parameter *g*, the prior generator *G_g_* takes as input a source of randomness *U* drawn from some distribution *P_U_* and outputs (the parameters indexing) a distribution *P*. For each P∈P, this algorithm requires access to a generator *D_P_* that takes as input a source of randomness *Z* ~ *P_Z_* and outputs data *X* that has distribution *P*.

2: **for** *k* = 1 to *K–1* **do** ⊳Iteratively update the procedure and prior.

3: Sample *U* ~ *P_U_* and let *P_k_* = *G*_*g*(*k*)_(*U*). ⊳Draw *P_k_* from current prior.

4: Sample *Z* ~ *P_Z_* and let *X* = *D_P_k__*(*Z*). ⊳Draw data from *P_k_*.

5: Update the parameter *t*(*k*) indexing the procedure by moving in the opposite direction of ∇_*t*(*k*)_*L*(*T*_*t*(*k*)_(*X*),*P_k_*). For example, *t*(*k*) could be updated via stochastic gradient descent

$$t(k+1)=t(k)-\frac{1}{\sqrt{k}}\nabla_{t(k)}L(T_{t(k)}(X),P_{k})$$ (10)

6: Update the parameter *g*(*k*) indexing the generator function by moving in the direction of ∇_*g*(*k*)_*L*(*T*_*t*(*k*+1)_(*X*), *P_k_*), where the gradient takes into account the fact that *P_k_* relies on *g*(*k*) through *G*_*g*(*k*)_ and *X* relies on *g*(*k*) through *P_k_*. To make this dependence explicit, we can write this gradient as ∇_*g*(*k*)_*L*(*T*_*t*(*k*+1)_(*D*_*G*_*g*(*k*)_(*U*)_(*Z*)), *G*_*g*(*k*)_(*U*)).

The parameter *g*(*k*) can, for example, be updated via stochastic gradient ascent

$$g(k+1)=g(k)+\frac{1}{\sqrt{k}}\nabla_{g(k)}L(T_{t(k+1)}(D_{G_{g(k)}(U)}(Z)),G_{g(k)}(U))$$ (11)

7: **end for**

8: **return** the procedure *T*_*t*(*K*)_.

### Overview of methods for numerical experiments

The presentation of the methods used in each numerical experiment is broken into two parts. First, we present the implementation of the meta-learner used in the example. Second, we describe how we interrogate these estimators. In general, two layers of interrogation were used: Shallow interrogations, with relatively few initializations or a coarse grid on the parameter space, were produced for our learned estimators at many iterations to produce learning curves over time and to determine which estimator to select as the final reported estimator; a deep interrogation, with many more initializations or a much finer grid on the parameter space, was performed for the final selected estimator to more accurately assess its performance. Details for our point estimation and prediction experiments are given in the next two sections. Details for our confidence region construction experiments are given in Supplementary Appendix C.

We learned and interrogated our procedures in Julia (*40*). The neural networks for the point estimation and prediction experiments were fitted using Knet (*41*), and the neural networks for the confidence region construction experiments were fitted using Flux (*42*). Adam (*23*) was used to update all procedure and prior neural networks. In the notation of (*23*), we used parameter settings β_2_ = 0.999 and ϵ = 10^−8^ throughout. We varied the learning rate α_0_ and the exponential decay rate β_0_ across settings—we note that these quantities were referred to as α and β_1_, respectively, in (*23*), but here, we refer to them as α_0_ and β_0_ to avoid a notational overload.

### Methods for point estimation experiments

We first define the meta-learner implementations. We parameterized both the generator from the prior distribution and the estimator using multilayer perceptrons. The architectures of the generator networks are displayed in the top panel of fig. S5. The networks take independent Normal(0.5, 0.25) random variables as input and output the parameters indexing a distribution in the model. The estimator network takes as input *n* observations and outputs an estimate of the parameter of interest. The architectures of the estimator networks are displayed in the bottom panel of fig. S5. In the example in which the MLE of the unknown parameter γ is inconsistent, the output of the final learned procedures was truncated at the known upper bound enforced by the model, namely, γ = 2.

In all examples except the one in which the goal was to estimate a regression coefficient, we used Adam with parameters α_0_ = 10^−3^ and β_0_ = 0.5 to respectively maximize and minimize the Bayes MSE

$$R^{B}(T,\Pi )=E_{\Pi }[{\left\{T(X)-S(P)\right\}}^{2}]$$ (12)

Here, S(*P*) corresponds to the summary of the distribution *P* drawn from the prior Π. When estimating the regression coefficient, we instead used parameters α_0_ = 10^−4^ and β_0_ = 0 when updating the prior, and α_0_ = 10^−4^ and β_0_ = 0.5 when updating the procedure. An iteration consists of first making one Adam update to the prior network and subsequently making 10 updates to the estimator network.

Unbiased estimates of this risk were obtained by taking 1000 draws of *P* ~ Π and, for each of these draws, taking one draw of *X* ~ *P*. Upon initial fitting of the prior network, we observed that sometimes this network would collapse to a single distribution *P*, which had the effect of making the estimator always return the corresponding S(*P*). This phenomenon is referred to as mode collapse in the GAN literature (*33*). To avoid mode collapse on the prior distribution Π, we regularized the risk $R^{B}$(*T*, Π) using an estimate of 75[{$R^{B}$(*T*, Π_0_) − $R^{B}$(*T*, Π)}^+^]^2^, where Π_0_ is the reference prior given by the initial prior generator network and *z*^+^ = *z*𝟙{*z* > 0}. The estimate replaces $R^{B}$(*T*, Π_0_) and $R^{B}$(*T*, Π) by Monte Carlo approximations based on 1000 draws of *P* ~ Π and *X* ~ *P*. The logic of this regularization term is as follows. When Π begins to collapse toward a single distribution *P*, the estimator will also begin to collapse toward S(*P*), thereby causing the estimator to perform very poorly in other parts of the parameter space. As Π should be the least favorable prior distribution for the estimator, it should certainly be less favorable than Π_0_. Therefore, the penalty plays a role in the optimization when the prior is clearly underperforming.

We now describe the interrogation strategies used for the experiments in which the observations are Gaussian. When performing the shallow interrogation shown in fig. S1, we ran Adam to approximate the (μ, σ) at which the procedure’s MSE was largest. These Adam runs used a batch size of 100 and parameters α_0_ = 0.01 and β_0_ = 0.9. We performed 1000 updates for each of 50 starting values. The estimate of the least favorable (μ, σ) was defined as the (μ, σ) at which the procedure had the highest estimated risk based on this shallow interrogation. We subsequently estimated the maximal risk in this shallow interrogation by evaluating the risk of the procedure at this value of (μ, σ) using a test set of 5 × 10^4^ datasets to evaluate estimator performance.

The deep interrogation of the final chosen estimator of μ or σ at *n* = 50 was conducted using a grid search of the (μ, σ) parameter space. We started by selecting the iteration that we would define as our final estimator. To do this, we ran a shallow interrogation using a rectangular grid where each point is 0.125 away from its neighbors in both μ and σ coordinates, and the risk at each point was approximated using 10^4^ Monte Carlo replicates. We ran this search after every batch of 25 iterations when estimating μ and after every 400 iterations when estimating σ. We found the iteration at which the maximal risk over the grid was minimal. We then performed a deep interrogation via a finer grid search to improve our interrogation of the estimators learned at this iteration. When estimating both μ and σ, the shallow grid searches indicated that the worst-case risk occurred when σ is at the upper edge of the parameter space (namely, 3.9 ≤ σ ≤ 4). Therefore, for the deep interrogation of the final estimators, we ran the grid search again over (μ, σ) in [ − 5,5] × [3.9,4.0] using a finer grid of width 0.01 in each coordinate, and the risk at each point in the grid was approximated using a greater number (5 × 10^4^) of Monte Carlo replicates.

For the experiments where *n* = 1 and the goal is to estimate μ, we performed a grid search on the possible values of μ to approximate the maximal risk. We used a grid of width 10^−4^ and approximated the risk of the estimator at a given value of μ using 5 × 10^4^ random datasets. We performed this grid search after 10, 20, …, 100 thousand iterations. We then evaluated the maximal risk of the estimator by (i) finding the iteration index *j* at which the maximal risk over this grid is minimal and (ii) running the Monte Carlo approximation again to obtain a final maximal risk estimate.

A grid search was used for the deep interrogation of our learned procedures at iteration 5 × 10^5^ in the example in which the MLE is inconsistent. The grid of γ values was taken to be {0,10^−3^, 2 × 10^−3^, …, 2}. For each γ, MSE was approximated using 10^5^ Monte Carlo draws.

A random search was used in the setting where the goal is to estimate a regression coefficient. We used two different distributions to draw candidate least favorable β = (β_1_, β_2_) vectors—one of these distributions simulated β uniformly from the two-dimensional closed 𝓁_2_ sphere with radius 10, and the other simulated (β_1_, β_2_, β_3_) uniformly from the surface of the three-dimensional ℓ_2_ sphere with radius 10 and treated β_3_ as a dummy variable that was used to determine the magnitude of (β_1_, β_2_) but was subsequently ignored. We drew 5 × 10^4^ candidate β vectors from each of these distributions and evaluated the risk at each β using 2 × 10^3^ Monte Carlo draws. For the existing procedures that we considered, namely, OLS and ridge regression, we only drew 10^4^ candidate β vectors from each distribution and used 5 × 10^3^ Monte Carlo draws to evaluate performance.

In all settings, we compared the performance of our learned estimators to that of the MLE that knows the bounds on the parameter space. When *n* = 1 and the goal is to estimate μ, we also compared the performance of our learned procedure to that of the minimax optimal estimator, for which the maximal risk is presented in (*43*).

### Methods for prediction experiments

The simulation settings are summarized in Table 1. We now describe the implementation of our meta-learner in the binary prediction problem—this meta-learner returns an estimator mapping from the *n* observations to an estimate of the conditional expectation function *w*→E(*Y* | *W* = *w*) of the outcome *Y* given the predictor vector *W*. We restricted ourselves to prediction algorithms that map from the data to a vector $\hat{\gamma }$ indexing the neural network describing the conditional expectation Eγ(*Y* | *W*) in the statistical model and subsequently used *w*→E(*Y* | *W* = *w*) as our prediction function.

Our estimator network was an LSTM with a forget gate (*22*). In contrast to many settings where LSTMs are used, there was no sequential relationship between our observations. Instead, we induced an ordering *i* = 1,2, …, *n* in our *n* observations. The architecture of the estimator network is shown in fig. S6. Briefly, the network first passes observations *i* = 1,2, …, *n*, *n* + 1, …,3*n*/2 through an LSTM layer. The first *n*/2 inputs were used to initialize the cell state. The hidden states from the final *n* inputs were linearly transformed to match the dimension *d* of γ, passed through a mean pooling layer, and transformed element-wise via a rescaled sigmoid function to respect the known bounds $\left[\pm b_{1}]\times \prod_{j=1}^{d-1}[\pm b_{2}\right]$ on γ, where *b*_1_ and *b*_2_ are defined in Table 1. Specifically, this rescaled sigmoid function takes the form ϕ*_r_* : *z* ↦ (2*b*_1_/{1 + exp ( − *x*)} − *b*_1_,2*b*_2_/{1 + exp ( − *x*)} − *b*_2_, …,2*b*_2_/{1 + exp ( − *x*)} − *b*_2_). Using the LSTM as described allowed us to approximate an estimator of $\phi_{r}^{-1}(\gamma )$ given by $\hat{c}+\frac{1}{n}\sum_{i=1}^{n}\hat{f}(X_{i})$, where the constant $\hat{c}$ and the function $\hat{f}$ depend on the *n* observations. This form of estimator is reminiscent of a one-step estimator (*3*) of $\phi_{r}^{-1}(\gamma )$, which is an asymptotically efficient estimation strategy in many settings.

The LSTM was updated using a nested minimax algorithm based on the risk in Eq. 2. When updating the LSTM weights, we used parameters α_0_ = 10^−3^ and β_0_ = 0.9 for Adam. At each iteration, we identified an unfavorable distribution and made two Adam updates to the LSTM network to improve the procedure’s performance at this distribution. We obtained an unbiased gradient estimate using the following three-step approach: (i) draw a dataset from this unfavorable distribution, (ii) evaluate the current estimator at these datasets, and (iii) evaluate the risk by independently drawing 100 random values of the predictor vector *W* from the known marginal distribution. For each gradient update, we averaged 500 Monte Carlo repetitions of this three-step approach in settings x, xi, and xii and averaged 1000 repetitions in all other settings.

For each iteration, we used a random search to identify an unfavorable distribution. Specifically, we evaluated the performance of the procedure at *s*_1_ values of γ drawn uniformly from the parameter space, *s*_2_ values of γ drawn uniformly from the vertices of the hyperrectangle defining the parameter space, and *s*_3_ values of γ where each of the *d* = dim(γ) coordinates is drawn from an equal mixture between a discrete distribution placing equal mass on the upper and lower bounds on that coordinate of the parameter and a uniform draw from the interval connecting the upper and lower bounds on that coordinate of the parameter. At each of these γ values, the risk was approximated via 1000 Monte Carlo repetitions of the three-step approach described in the previous paragraph. The values of *s*_1_, *s*_2_, and *s*_3_ used in each setting appear in Table 1.

Each meta-learner was allowed to run until diagnostic output suggested that the worst-case performance of the estimator had stabilized or until our computational budget necessitated that we discontinue the run. The number of iterations increased with the complexity of the setting: approximately 2 × 10^4^ in settings x, xi, and xii; approximately 4 × 10^4^ in settings i, iv, v, and vi; and approximately 7.5 × 10^4^ in settings ii and iii.

For settings ii and iii, we also tried learning aware procedures that were identical to those described earlier in this section, except that, at each time *i* of the LSTM, the LSTM was provided both with the data point *X*_(*i* mod *n*)_ and the vector of coefficients from an OLS regression of the outcome regressed against an intercept, linear and quadratic main terms, and a linear interaction. These coefficients take the form (*Z*^⊤^*Z*)^−1^*Z*^⊤^*Y* for a design matrix *Z*. The population-level value of the matrix (*Z*^⊤^*Z*)^−1^ is known in the statistical model used in this example, namely, the model where the marginal distribution of the predictor vector is known. Hence, to speed computation, we used this known population-level quantity when computing the coefficients. The rationale behind using the OLS coefficients rather than the coefficients from a logistic regression was that the OLS estimates can be computed more quickly, thereby allowing us to make more updates to our procedure. Because evaluating the performance of these procedures was a secondary objective, we ran these meta-learners for fewer iterations than the procedures that do not receive OLS coefficients as input. In particular, the learners were updated over 3 × 10^4^ iterations.

In the clustering example, the procedure network was parameterized as a multilayer perceptron with an identity activation function consisting of three hidden layers, respectively, consisting of 40, 20, and 40 rectified linear units (ReLUs). The prior network was parameterized as a multilayer perceptron with one hidden layer consisting of 25 ReLUs. The procedure network returned a 10-dimensional array of probabilities, and the prior network returned the means (μ_1_, μ_2_) of the first and second components of the Gaussian mixture. For a (μ_1_, μ_2_) pair, a mixture weight ω was drawn uniformly from (0, 1). We made 2 × 10^5^ Adam updates to the prior network, and for each of these updates, we made 25 updates to the procedure network. Both Adam optimizers used parameters α_0_ = 0.001 and β_0_ = 0.5.

We used two strategies for interrogating the learned binary prediction procedures. The first involved running a variant of the Luus-Jakola (LJ) optimization procedure, which is a gradient-free, heuristic global optimization procedure that has been shown to perform well in nonconvex optimization problems (*44*). When LJ was used, an initial γ_0_ was selected uniformly in the parameter space. Then, the value γ*_j_* was iteratively updated for *j* = 0,1, …,149 using the following procedure. Let *R_j_* denote a rectangle centered at γ*_j_* for which each edge has length 0.95*^j^* times the width of the total parameter space for that dimension of the parameter indexing the conditional expectation *E*(*Y* | *W*) implied by the statistical model. Let ${\bar{R}}_{j}$ denote the intersection of *R_j_* with the parameter space. The point γ_*j*,1_ was selected uniformly at random from ${\bar{R}}_{j}$. The point γ_*j*,2_ was selected at random from the 2*^d^* vertices of ${\bar{R}}_{j}$. The risk of the procedure at γ_*j*,1_, γ_*j*,2_, and γ*_j_* was evaluated using 2500 + 50(*j* + 1) Monte Carlo repetitions. Then, γ_*j*+1_ was defined as the maximizer of the risk among γ_*j*,1_, γ_*j*,2_ and γ*_j_*. Last, *j* was set to *j* + 1, and the iterative procedure was repeated.

The second interrogation strategy involved learning an unfavorable prior network for the prediction procedure. This interrogation strategy differed from learning the prior in an alternating algorithm in that the statistical procedure was fixed, that is, it was not updated over time. The prior network was parameterized as a multilayer perceptron with two hidden layers, each consisting of 20 rectifier linear units. The network takes as input a three-dimensional vector of independent noise variables, two of which follow a Normal(0.5, 0.25) distribution and the other follows a Rademacher distribution. The Adam updated to the prior network used parameters α_0_ = 0.002 and β_0_ = 0.9. The optimization was discontinued if either 10^4^ update steps had been performed or if the distance between two exponential moving averages of the Bayes risk under the priors became small. The exponential moving averages $B_{1}^{j}$ and $B_{2}^{j}$ were initialized to zero and, at iteration *j*, were respectively updated using the Bayes risk $R^{B}$(*T*, Π) = *E*_Π*^j^*_[R(*T*, *P*)] of the procedure *T* under the current prior Π*^j^* as $B_{1}^{j+1}=0.995B_{1}^{j}+0.005R^{B}(T,\Pi^{j})$ and $B_{2}^{j+1}=0.98B_{2}^{j}+0.02R^{B}(T,\Pi^{j})$. The procedure was discontinued if $B_{2}^{j}-B_{1}^{j}<10^{-5}$. After the final prior network was obtained, 500 γ parameters were drawn from this prior, and the risk at these parameters was calculated via 2500 Monte Carlo repetitions. The final selected unfavorable distribution was the distribution indexed by the value of γ for which this maximal risk estimate was largest. The final risk of this estimator was assessed via 5000 additional Monte Carlo repetitions at this unfavorable distribution.

We used LJ for the shallow interrogation for settings ii and iii, and the prior network was used for the shallow interrogation approach in all other settings. The number of initializations used in these shallow interrogations is shown in Table 1. A shallow interrogation was performed after every 200 iterations for the unaware approaches and after every 1000 iterations for the aware approaches. The final selected procedure corresponded to the procedures with the minimal maximal risk in these shallow interrogations. For settings ii and iii, we restricted consideration to the shallow interrogations of the last 10^4^ iterations. For the deep interrogation, in each setting, we ran LJ with 50 initializations and the prior network approach using 250 initializations. We also report the Bayes risk for each method corresponding to a prior that draws γ uniformly from the parameter space. This Bayes criterion has the benefit of not relying on finding a near-maximizer of a nonconcave risk surface.

We also interrogated existing prediction procedures. In each setting, we evaluated the performance of the MLE, which was found using the BOBYQA optimization routine in the nloptr R package (*45*). Before running the routine, 100 candidate starting values of γ were drawn uniformly at random from the parameter space, and the value of γ that maximized the likelihood was selected as the initial value. We also interrogated a main-term logistic regression and lasso using cross-validated tuning parameter selection (*31*), where we truncated the estimated conditional expectations from both of these methods to enforce the bounds [0.1,0.9] implied by the model. Because all of these existing procedures took substantially longer to evaluate than learned procedures, we were not able to use deep interrogations to evaluate their performance. Instead, for each existing method, we ran LJ with 3 initializations and 100 (rather than 150) iterations of the recursive procedure that was used to find an unfavorable γ. Given the more complex settings considered in settings ii and iii, for these two settings, we also evaluated the risk of the existing methods at the least favorable γ identified for our learned procedures. The fact that we could only run shallow interrogations for the existing methods may lead to overly optimistic estimates of their performance and therefore overly pessimistic estimates of the relative performance of our learned procedures.

A grid search was used to interrogate the learned clustering procedure. For each grid point, the performance of the procedure on 10^4^ datasets was used to approximate the risk. In the hierarchical setting, for each dataset, ω was drawn from a standard uniform, whereas, in the nonhierarchical setting, fixed values of ω = 0,0.1, …,0.5 were considered. In all cases, a 100 × 100 grid of (μ_1_, μ_2_) on [−3,3]^2^ was used.

### Data applications

We start by describing the datasets used in our data applications. The Titanic survival data are publicly available as the titanic3 dataset in the Hmisc R package (*34*). It consists of information about 1309 Titanic passengers, of whom 500 survived. Two analyses were conducted. The first analysis used age and fare to predict passenger survival. A complete-case analysis was performed, resulting in a total of 1045 passengers in the dataset. The second analysis used a 10-dimensional predictor, namely, an indicator of having (i) a first-class ticket or (ii) a second-class ticket, (iii) age, (iv) a binary sex variable, (v) number of siblings or spouses onboard, (vi) number of parents or children onboard, (vii) fare, an indicator of whether a passenger embarked (viii) from Southampton or (ix) from Cherbourg, and (x) an indicator of whether any missing variables were imputed. The only missing variables in this analysis were age and fare, and in this second analysis, these missing values were imputed using median imputation, where the imputation for fare was stratified by ticket class.

HVTN 070 was a multicenter randomized clinical trial of the PENNVAX-B DNA vaccine (PV). A total of 120 HIV-1 uninfected adults aged 18 to 50 years were administered placebo, PV alone, PV with interleukin-12 (IL-12) DNA plasmid, or PV with one of two dose levels of IL-15 DNA plasmid. We focus on the individuals who received PV in combination with IL-12 or IL-15, pooling these three groups together into a group of 70 individuals. We restricted our analysis to the subset of 60 individuals for whom post-fourth vaccination HIV-specific CD4^+^ response measurements were available. We defined individuals as having a post-fourth vaccination CD4^+^ immune response if they had a response against at least one of HIV Env, Gag, or Pol at the post-fourth vaccination immunogenicity time point and as not having a response otherwise. Details on the intracellular cytokine staining method used to obtain these immune response measurements and to define the Env, Gag, and Pol response variables can be found in (*35*). Of the 60 individuals in our analysis, 23 had a CD4^+^ response.

We now describe the methods for our data applications. The learned prediction procedures from each setting in Table 1 were used for these analyses. Settings i, ii, and iii were used for the Titanic analysis with two predictors; settings iv, v, and vi were used for the Titanic analysis with 10 predictors; and settings x, xi, and xii were used for the HIV vaccine analysis. In all analyses, covariates were linearly rescaled to more closely match the predictor distributions assumed when learning the prediction procedures. In both Titanic analyses, observations were linearly recentered and scaled so that each variable marginally had empirical mean 0 and variance 1 within each training set of size 50. In the HIV vaccine analysis, BMI was rescaled to have empirical mean 0 and variance 1 within each training set of size 50, whereas the sex variable was rescaled so that females have value +1 and males have value −1.

A total of 2 × 10^4^ cross-validation splits were used for the Titanic analyses, whereas 10^3^ splits were used for the HIV vaccine analyses. Validation samples were sampled uniformly at random, and rejection sampling was used to ensure that each validation sample had at least one positive and one negative outcome. Wald-type 95% CIs reported for the cross-validated cross-entropy and AUC. Different standard errors (SEs) were used for the Titanic and HIV vaccine analyses. For the Titanic analyses, the population is treated as finite, that is, consisting of the 1309 passengers in the dataset, and the SEs reflect uncertainty resulting from only drawing a subset of the collection of draws of 50 passengers from this population. Therefore, SEs were calculated using the variance of the performance estimates across folds. For the HIV vaccine analyses, SEs were calculated using the cvAUC R package (*46*) to account for the uncertainty resulting from drawing the 60 individuals from a large superpopulation.

### Technical results for convergence guarantees in function space

We now describe some technical results that are needed to interpret and prove the result of Theorem 1. Result 1 provides an expression for the gradient of the risk functional at a distribution *P* when the risk can be written as an expected loss function. Lemma 1 shows that, as would be expected, the remainder from a first-order expansion is nonnegative for convex functionals. Lemma 2 shows that the gradient of the risk functional at an unfavorable distribution helps describe the behavior of the maximal risk functional. Specifically, this lemma shows that the gradient of the risk functional at an unfavorable distribution is approximately a subgradient of the maximal risk functional when the risk functional is convex. We conclude this section by proving Theorem 1.

For ease of readability, we ignore measurability concerns in the main text, with the understanding that minor modifications would be needed to make these results precise. We are more careful about measurability for the choice of dominating measure proposed in Supplementary Appendix E.2.

We now give an expression for the gradient of the risk function. The validity of this expression relies on regularity conditions that allow us to interchange differentiation and integration. This expression also relies on a definition. In particular, for a measure ξ with support on A, *L*^2^(ξ) is used to denote the collection of functions f:A→ℝ for which ∫*f* (*a*)^2^ *d*ξ(*a*) < ∞.

**Result 1.** Fix P∈P and suppose ℋ = *L*^2^(ξ) for a measure ξ. Suppose that the risk functional at *P* takes the form $S\mapsto E_{P}[\int {ℒ}_{P}(S,X,a)d\xi (a)]$ for a function ${ℒ}_{P}:S\;\times \;X\;\times \;A\to \mathbb{R}$ and further suppose that there exists a function ${\dot{ℒ}}_{P}:S\;\times \;X\;\times \;A\to \mathbb{R}$ such that ${\frac{d}{d\zeta }{ℒ}_{P}(S+\zeta h(x,a))|}_{\zeta =0}=h(x,a){\dot{ℒ}}_{P}(S,x,a)$ for all h∈S and (*x*, *a*). Under some regularity conditions, condition A1 holds and the gradient of the risk functional at *P* is given by

$$g(S,P):(x,a)\mapsto {\dot{ℒ}}_{P}(S,x,a)\frac{\mathrm{dP}}{d\nu }(x)$$ (13)

Furthermore, if $S\mapsto {ℒ}_{P}(S,x,a)$ is convex for all (x,a)∈X×A, then the risk functional at *P* is convex.

The above result shows that the choice of dominating measure ν generally matters because the form of the subgradient relies on ν. In Results, we claimed that condition A4 would be most plausible when

$$0\leq \underset{P\in P,x\in X}{\text{sup}}|\frac{\mathrm{dP}}{d\nu }(x)|\leq M_{1}$$ (14)

for some *M*_1_ < +∞. In the setting of the above result, we then have that

$$\begin{array}{c} {\left\|g(S,P)\right\|}^{2}=\int \langle {\dot{ℒ}}_{P}(S,x,\cdot ),{\dot{ℒ}}_{P}(S,x,\cdot )\rangle {\left\{\frac{\mathrm{dP}}{d\nu }(x)\right\}}^{2}d\nu (x)\\ \leq M_{1}^{2}\int \langle {\dot{ℒ}}_{P}(S,x,\cdot ),{\dot{ℒ}}_{P}(S,x,\cdot )\rangle d\nu (x)=M_{1}\|(x,a)\mapsto {\dot{ℒ}}_{P}\left(S,x,a\right){\|}^{2} \end{array}$$ (15)

Hence, in the special case that $\hat{g}$ is deterministic, condition A4 is satisfied, provided that Eq. 14 holds and $\left\|(x,a)\mapsto {\dot{ℒ}}_{P}(S,x,a)\right\|$ is uniformly bounded over S∈S and P∈P.

The gradient is small at *x* if the likelihood $\frac{\mathrm{dP}}{d\nu }(x)$ is small. Consequently, the subgradient update to *S_k_* in Eq. 6 will only change the behavior of *S_k_* for datasets that could plausibly have been generated by *P*. To understand the function ${\dot{ℒ}}_{P}$ that appears in the expression for the gradient, we may consider the special case of a squared-error loss ${ℒ}_{P}(S,x,a)={\left\{S(x,a)-f_{P}(a)\right\}}^{2}$, where $f_{P}:A\to \mathbb{R}$ is the feature of *P* that we wish to estimate. In this case, ${\dot{ℒ}}_{P}(S,x,a)=2\{S(x,a)-f_{P}(a)\}$ so that the gradient is positive when the procedure overestimates the feature of interest and is negative when it underestimates this feature. Therefore, the behavior of the procedure when the data are generated by the distribution *P* can be improved by moving in the opposite direction of the gradient at this distribution. The update step in Eq. 6 leverages this by aiming to improve the behavior of the procedure at an unfavorable distribution.

*Sketch of Proof of Result 1.* Fix a direction h∈S. Under some regularity conditions,

$$\begin{array}{cc} \delta_{h}R(S,P) & =\iint {\frac{d}{d\zeta }{ℒ}_{P}(S+\zeta h,x,a)|}_{\zeta =0}d\xi (a)\mathrm{dP}(x)\\ & =\iint {\dot{ℒ}}_{P}(S,x,a)h(x,a)d\xi (a)\mathrm{dP}(x)\\ & =\iint {\dot{ℒ}}_{P}(S,x,a)\frac{\mathrm{dP}}{d\nu }(x)h(x,a)d\xi (a)d\nu (x)\\ & =\langle g(S,P),h\rangle \end{array}$$ (16)

Thus, provided g(S,P)∈S, the Riesz representation theorem shows that condition A1 holds and that the gradient of the risk functional at *P* is equal to *g*(*S*, *P*).

The fact that the risk functional at *P* is convex if $S\mapsto {ℒ}_{P}(S,x,a)$ is convex for all x∈X follows from the fact that a non-negatively weighted linear combination of convex functions is itself convex.

For procedures *S* and $\tilde{S}$, we define the remainder term in a linear expansion of the risk functional as

$$\text{Rem}(S,\tilde{S};P)=R(\tilde{S},P)-R(S,P)-\langle g(S,P),\tilde{S}-S\rangle$$ (17)

We now show that this remainder is nonnegative for convex functionals.

**Lemma 1.** If conditions A1 and A2 hold, then $\text{Rem}(S,\tilde{S};P)\geq 0$ for all P∈P and all procedures *S* and $\tilde{S}$.

*Proof.* Fix procedures *S* and $\tilde{S}$ and a distribution P∈P. For all *t* ∈ [0,1]

$$\begin{array}{cc} 0 & \leq (1-t)R(S,P)+tR(\tilde{S},P)-R((1-t)S+t\tilde{S},P)\\ & =-\{R((1-t)S+t\tilde{S},P)-R(S,P)\}+t\{R(\tilde{S},P)-R(S,P)\}\\ & =-\text{Rem}(S,(1-t)S+t\tilde{S};P)+\text{Rem}(S,\tilde{S};P) \end{array}$$ (18)

where the final equality twice used Eq. 17 and also used that the first-order component of the expansions for the two differences on the second to last line are equivalent. Rearranging and using condition A2, $\text{Rem}(S,(1-t)S+t\tilde{S})\leq \text{Rem}(S,\tilde{S};P)$ for all *t* ∈ [0,1]. Letting *t* equal 0 gives the result because Rem(*S*, *S*) = 0.

We now establish that *g*(*S*, *P*) helps describe the behavior of $R^{⋆}$ in a neighborhood of a given estimator if *P* is unfavorable for *S*. In particular, we establish that if *P* is unfavorable, then *g*(*S*, *P*) is approximately a generalized gradient (*47*) of $R^{⋆}$ at *S*. Under the convexity condition A2, this result will establish that *g*(*S*, *P*) is an approximate subgradient.

**Lemma 2.** (Approximate generalized gradient of risk). Fix a procedure *S*, h∈S, P∈P, and a real-valued ζ. If condition A1 holds, then

$$\begin{array}{c} R^{⋆}(S+\zeta h)-R^{⋆}(S)-\zeta \langle g(S,P),h\rangle \\ \geq b(\zeta )-[R^{⋆}(S)-R(S,P)] \end{array}$$ (19)

where *b*(ζ)/ζ → 0 as ζ → 0. If condition A2 also holds, then *b*(ζ) = 0 for all ζ.

*Proof.* By definition, we have that $R^{⋆}$(*S* + ζ*h*) ≥ R(*S* + ζ*h*, *P*). Hence,

$$\begin{array}{c} R^{⋆}(S+\zeta h)-R^{⋆}(S)-\zeta \langle g(S,P)h\rangle \\ \geq R(S+\zeta h,P)-R(S,P)-\zeta \langle g(S,P),h\rangle -[R^{⋆}(S)-R(S,P)]\\ =\text{Rem}(S,S+\zeta h;P)-[R^{⋆}(S)-R(S,P)] \end{array}$$ (20)

Under condition A1, the Riesz representation theorem indicates that Rem(*S*, *S* + ζ*h*; *P*) = *o*(ζ). By Lemma 1, Rem(*S*, *S* + ζ*h*; *P*) ≥ 0 if condition A2.

*Proof of Theorem 1*. Fix $S^{⋆}\in S^{⋆}$ and a natural number *k*. We write E*_k_* to denote an expectation over *Q_k_* conditionally on *S_k_* and *P_k_*. We observe that

$$\begin{array}{c} E_{k}{\left\|S_{k+1}-S^{⋆}\right\|}^{2}=E_{k}{\left\|S_{k}-\zeta_{k}{\hat{g}}_{k}-S^{⋆}\right\|}^{2}\\ ={\left\|S_{k}-S^{⋆}\right\|}^{2}+2\zeta_{k}\langle E_{k}({\hat{g}}_{k}),S^{⋆}-S_{k}\rangle +\zeta_{k}^{2}E_{k}{\left\|{\hat{g}}_{k}\right\|}^{2}\\ \leq 2\zeta_{k}[R^{⋆}(S^{⋆})-R^{⋆}(S_{k})-\text{Rem}(S_{k},S^{⋆};P_{k})+\{R^{⋆}(S_{k})-R(S_{k},P_{k})\}]\\ +{\left\|S_{k}-S^{⋆}\right\|}^{2}+\zeta_{k}^{2}E_{k}{\left\|{\hat{g}}_{k}\right\|}^{2} \end{array}$$ (21)

where the inequality uses that ${\hat{g}}_{k}$ is unbiased for *g_k_* as well as Lemma 2 with *S* = *S_k_*, ζ = 1 and *h* = *S*^⋆^ − *S_k_*. Using condition A2, Lemma 1, and condition A4

$$\begin{array}{c} E_{k}{\left\|S_{k+1}-S^{⋆}\right\|}^{2}\leq {\left\|S_{k}-S^{⋆}\right\|}^{2}-2\zeta_{k}\{R^{⋆}(S_{k})-R^{⋆}(S^{⋆})\}\\ +\zeta_{k}[M\zeta_{k}+2\{R^{⋆}(S_{k})-R(S_{k},P_{k})\}] \end{array}$$ (22)

After taking an expectation on both sides over $\prod_{j=1}^{k-1}Q_{j}$, an induction argument shows that

$$\begin{array}{c} E{\left\|S_{K+1}-S^{⋆}\right\|}^{2}\leq {\left\|S_{1}-S^{⋆}\right\|}^{2}-2\sum_{k=1}^{K}\zeta_{k}\{E[R^{⋆}(S_{k})]-R^{⋆}(S^{⋆})\}\\ +\sum_{k=1}^{K}\zeta_{k}(M\zeta_{k}+2\epsilon_{k}) \end{array}$$ (23)

where here and in the remainder *E* denotes an expectation over $\prod_{j=1}^{\infty }Q_{j}$. Bounding the left-hand side below by zero and rearranging

$$\begin{array}{c} 2\sum_{k=1}^{K}\zeta_{k}\{E[R^{⋆}(S_{k})]-R^{⋆}(S^{⋆})\}\\ \leq {\left\|S_{1}-S^{⋆}\right\|}^{2}+\sum_{k=1}^{K}\zeta_{k}(M\zeta_{k}+2\epsilon_{k}) \end{array}$$ (24)

As *S*^⋆^ was an arbitrary element of $S^{⋆}$, we can take an infimum over $S^{⋆}\in S^{⋆}$ on the right-hand side. The left-hand side is bounded below by $2\{{\text{min}}_{k}E[R^{⋆}(S_{k})]-R^{⋆}(S^{⋆})\}\sum_{k=1}^{K}\zeta_{k}$. Dividing both sides by $2\sum_{k=1}^{K}\zeta_{k}$ gives Eq. 7.

Suppose now that condition A6 holds, and fix β > 0. Because max {ζ*_K_*, ϵ*_K_*} → 0, there exists a natural number *K*_1_ such that max {*M*ζ*_k_*,2ϵ*_k_*} < β for all *k* > *K*_1_. Because $\sum_{k=1}^{K}\zeta_{k}$ diverges, there exists a natural number *K*_2_ such that, for all *K* > *K*_2_

$$\sum_{k=1}^{K}\zeta_{k}\geq \frac{1}{\beta }\{\rho {\left(S_{1},S^{⋆}\right)}^{2}+{\left\|S_{1}-S^{⋆}\right\|}^{2}+\sum_{k=1}^{K_{1}}\zeta_{k}(M\zeta_{k}+2\epsilon_{k})\}$$ (25)

Using Eq. 7, for all *K* ≥ max {*K*_1_, *K*_2_}

$$\begin{array}{c} \underset{k=1,\ldots ,K}{\text{min}}E[R^{⋆}(S_{k})]-\underset{S\in S}{\text{inf}}R^{⋆}(S)\\ \leq \frac{\rho {\left(S_{1},S^{⋆}\right)}^{2}+\sum_{k=1}^{K_{1}}\zeta_{k}(M\zeta_{k}+2\epsilon_{k})}{2\sum_{k=1}^{K}\zeta_{k}}+\frac{\sum_{k=K_{1}+1}^{K}\zeta_{k}(M\zeta_{k}+2\epsilon_{k})}{2\sum_{k=1}^{K_{1}}\zeta_{k}+2\sum_{k=K_{1}+1}^{K}\zeta_{k}} \end{array}$$ (26)

By using the choice of *K*_2_ to bound the denominator below in the first term, the first term is bounded above by β/2. For the second term, we first bound the denominator below by $2\sum_{k=K_{1}+1}^{K}\zeta_{k}$ and subsequently used that the choice of *K*_1_ bounds the numerator above by $\beta \sum_{k=K_{1}+1}^{K}\zeta_{k}$. Hence, the latter term is bounded above by β/2. Thus, the left-hand side is no larger than β for all *K* large enough. As β > 0 was arbitrary, $\text{lim}\;{\text{sup}}_{K\to \infty }\{{\text{min}}_{k=1,\ldots ,K}E[R^{⋆}(S_{k})]-{\text{inf}}_{S\in S}R^{⋆}(S)\}\leq 0$. Because ${\text{min}}_{k=1,\ldots ,K}E[R^{⋆}(S_{k})]\geq {\text{inf}}_{S\in S}R^{⋆}(S)$ for all *k*, min_*k*=1,…, *K*_*E*[$R^{⋆}$(*S_k_*)] converges to ${\text{inf}}_{S\in S}R^{⋆}(S)$ as *K* → +∞.

## Supplementary Material

http://advances.sciencemag.org/cgi/content/full/6/9/eaaw2140/DC1

Download PDF

Movie S1

Learning to learn from data: Using deep adversarial learning to construct optimal statistical procedures

## SUPPLEMENTARY MATERIALS

Supplementary material for this article is available at http://advances.sciencemag.org/cgi/content/full/6/9/eaaw2140/DC1 Appendix A. Supplementary tables and figures for numerical experiments. Appendix B. Supplementary tables for data applications. Appendix C. Methods for confidence region construction experiments. Appendix D. Neural network architectures in numerical experiments. Appendix E. Further discussion of guarantees for nested minimax algorithms. Appendix F. An example showing challenges faced by existing nested maximin algorithms. Appendix G. Captions for additional file types. Fig. S1. Convergence of the risk of the learned estimators in the Gaussian estimation example. Fig. S2. Pointwise quantiles of the fit of our learned two-layer multilayer perceptron prediction function at *W*_2_ = 0 and different values of *W*_1_ based on *n* = 50 observations from four data-generating distributions. Fig. S3. Learning curves for worst-case (red) and uniform-prior Bayes (blue) prediction performance in the logistic regression settings i to xii shown in Table 1. Fig. S4. Performance of the learned 95% level confidence region procedure across different values of η. Fig. S5. Prior generator multilayer perceptrons and procedure multilayer perceptrons used for the point estimation examples. Fig. S6. Estimator LSTM used when estimating binary regressions. Fig. S7. Estimator LSTM used when defining the interior point of our confidence regions. Table S1. Gaussian model with σ^2^ = 1, |μ| ≤ *m*, and *n* = 1. Table S2. Final estimated performance of the learned prediction algorithms, the MLE, and a linear-logistic regression. Table S3. Performance of our learned procedures and of existing procedures in data illustrations. Movie S1. Evolution of the risk of the learned estimator of μ as the weights of the neural network are updated in the Gaussian model with *n* = 50 observations and unknown (μ, σ). References (*48*–*50*)
