# Supplementary material for: Learning to learn from data: Using deep adversarial learning to construct optimal statistical procedures
Source: Sci Adv. 2020 Feb 26;6(9):eaaw2140. doi: 10.1126/sciadv.aaw2140 (PMC7051830; doi:10.1126/sciadv.aaw2140)
Supplement: http://advances.sciencemag.org/cgi/content/full/6/9/eaaw2140/DC1 [file supp_6_9_eaaw2140__index.html]

Science Advances | Science AdvancesAAASSearchScience AdvancesMenu

## Supplementary Materials

**The PDF file includes:**

- Appendix A. Supplementary tables and figures for numerical experiments.
- Appendix B. Supplementary tables for data applications.
- Appendix C. Methods for confidence region construction experiments.
- Appendix D. Neural network architectures in numerical experiments.
- Appendix E. Further discussion of guarantees for nested minimax algorithms.
- Appendix F. An example showing challenges faced by existing nested maximin algorithms.
- Appendix G. Captions for additional file types.
- Fig. S1. Convergence of the risk of the learned estimators in the Gaussian estimation example.
- Fig. S2. Pointwise quantiles of the fit of our learned two-layer multilayer perceptron prediction function at *W*2 = 0 and different values of *W*1 based on *n* = 50 observations from four data-generating distributions.
- Fig. S3. Learning curves for worst-case (red) and uniform-prior Bayes (blue) prediction performance in the logistic regression settings i to xii shown in Table 1.
- Fig. S4. Performance of the learned 95% level confidence region procedure across different values of η.
- Fig. S5. Prior generator multilayer perceptrons and procedure multilayer perceptrons used for the point estimation examples.
- Fig. S6. Estimator LSTM used when estimating binary regressions.
- Fig. S7. Estimator LSTM used when defining the interior point of our confidence regions.
- Table S1. Gaussian model with σ2 = 1, |μ| ≤ *m*, and *n* = 1.
- Table S2. Final estimated performance of the learned prediction algorithms, the MLE, and a linear-logistic regression.
- Table S3. Performance of our learned procedures and of existing procedures in data illustrations.
- References (*48*–*50*)

Download PDF

**Other Supplementary Material for this manuscript includes the following:**

- Movie S1 (.mp4 format). Evolution of the risk of the learned estimator of μ as the weights of the neural network are updated in the Gaussian model with *n* = 50 observations and unknown (μ, σ).

**Files in this Data Supplement:**

- Adobe PDF - aaw2140\_SM.pdf
