# Supplementary material for: Learning to learn from data: Using deep adversarial learning to construct optimal statistical procedures
Source: Sci Adv. 2020 Feb 26;6(9):eaaw2140. doi: 10.1126/sciadv.aaw2140 (PMC7051830; doi:10.1126/sciadv.aaw2140)
Supplement: Download PDF [file aaw2140_SM.pdf]

Science Advances
AAAS

advances.sciencemag.org/cgi/content/full/6/9/eaaw2140/DC1

Supplementary Materials for

Learning to learn from data: Using deep adversarial learning to construct optimal statistical procedures

Alex Luedtke*, Marco Carone, Noah Simon, Oleg Sofrygin

*Corresponding author. Email: aluedtke@uw.edu

Published 26 February 2020, *Sci. Adv.* **6**, eaaw2140 (2020)
DOI: 10.1126/sciadv.aaw2140

The PDF file includes:

Appendix A. Supplementary tables and figures for numerical experiments.
Appendix B. Supplementary tables for data applications.
Appendix C. Methods for confidence region construction experiments.
Appendix D. Neural network architectures in numerical experiments.
Appendix E. Further discussion of guarantees for nested minimax algorithms.
Appendix F. An example showing challenges faced by existing nested maximin algorithms.
Appendix G. Captions for additional file types.
Fig. S1. Convergence of the risk of the learned estimators in the Gaussian estimation example.
Fig. S2. Pointwise quantiles of the fit of our learned two-layer multilayer perceptron prediction function at $W_2 = 0$ and different values of W_1 based on $n = 50$ observations from four data-generating distributions.
Fig. S3. Learning curves for worst-case (red) and uniform-prior Bayes (blue) prediction performance in the logistic regression settings i to xii shown in Table 1.
Fig. S4. Performance of the learned 95% level confidence region procedure across different values of η.
Fig. S5. Prior generator multilayer perceptrons and procedure multilayer perceptrons used for the point estimation examples.
Fig. S6. Estimator LSTM used when estimating binary regressions.
Fig. S7. Estimator LSTM used when defining the interior point of our confidence regions.
Table S1. Gaussian model with $\sigma^2 = 1$, $|\mu| \leq m$, and $n = 1$.
Table S2. Final estimated performance of the learned prediction algorithms, the MLE, and a linear-logistic regression.
Table S3. Performance of our learned procedures and of existing procedures in data illustrations.
References (*48–50*)

Other Supplementary Material for this manuscript includes the following:

(available at advances.sciencemag.org/cgi/content/full/6/9/eaaw2140/DC1)

Movie S1 (.mp4 format). Evolution of the risk of the learned estimator of μ as the weights of the neural network are updated in the Gaussian model with $n = 50$ observations and unknown (μ, σ).

Appendix A. Supplementary tables and figures for numerical experiments.

Table S1. Gaussian model with $\sigma^2 = 1$, $|\mu| \leq m$, and $n = 1$. Displaying maximum mean-squared error (*MMSE*) of the finite sample minimax *Optimal* estimator presented in (42), *Best Learned* (the alternating algorithm selecting the iteration that preliminary grid search suggested was best), *Final Learned* (the alternating algorithm selecting the final iteration), and the maximum likelihood estimator (*MLE*). *Error* indicates relative MMSE compared to that of Optimal. Observe that Best Learned outperforms Final Learned, sometimes dramatically, in all but two cases – in these cases, the preliminary grid search led us to select an estimator with slightly subpar performance.

	Optimal	Best Learned		Final Learned		MLE	
m	MMSE	MMSE	Error	MMSE	Error	MMSE	Error
0.10	0.010	0.010	3.4%	0.011	6.2%	0.018	80.4%
0.20	0.038	0.039	0.6%	0.039	1.8%	0.063	64.4%
0.30	0.083	0.083	0.4%	0.088	7.2%	0.125	50.9%
0.40	0.138	0.138	0.5%	0.149	8.0%	0.192	39.4%
0.50	0.199	0.200	0.6%	0.200	0.6%	0.258	29.7%
0.60	0.262	0.264	1.0%	0.281	7.6%	0.317	21.3%
0.70	0.321	0.324	1.1%	0.346	7.9%	0.368	14.6%
0.80	0.374	0.382	2.3%	0.396	6.0%	0.384	2.7%
0.90	0.417	0.426	2.2%	0.428	2.6%	0.451	8.1%
1.00	0.450	0.461	2.4%	0.460	2.3%	0.516	14.8%
1.05	0.461	0.481	4.3%	0.481	4.2%	0.547	18.6%
1.40	0.535	0.559	4.5%	0.556	3.9%	0.736	37.5%
1.50	0.556	0.573	3.0%	0.602	8.3%	0.778	40.0%
1.60	0.577	0.592	2.5%	0.604	4.7%	0.816	41.4%

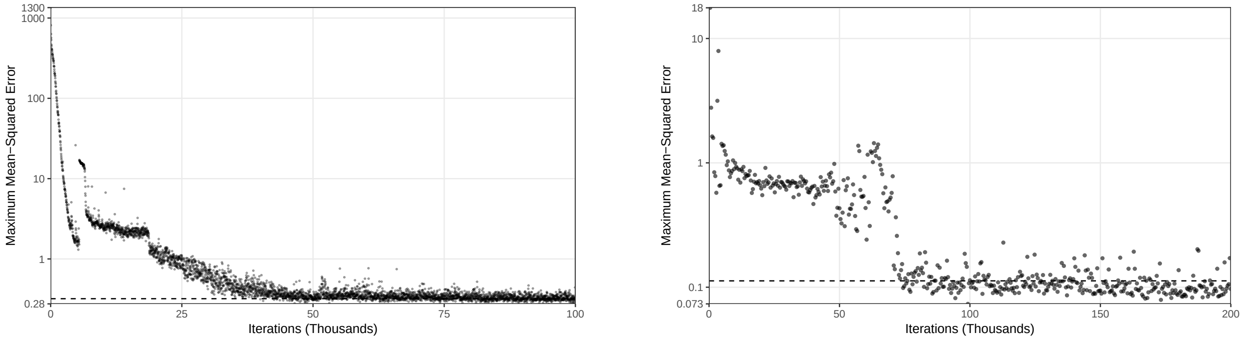

(A) Mean **(B)** Standard Deviation

Fig. S1. Convergence of the risk of the learned estimators in the Gaussian estimation example. Estimation of (**A**) μ and (**B**) σ when n=50. The maximal risk of the MLE is indicated by horizontal dashed lines.

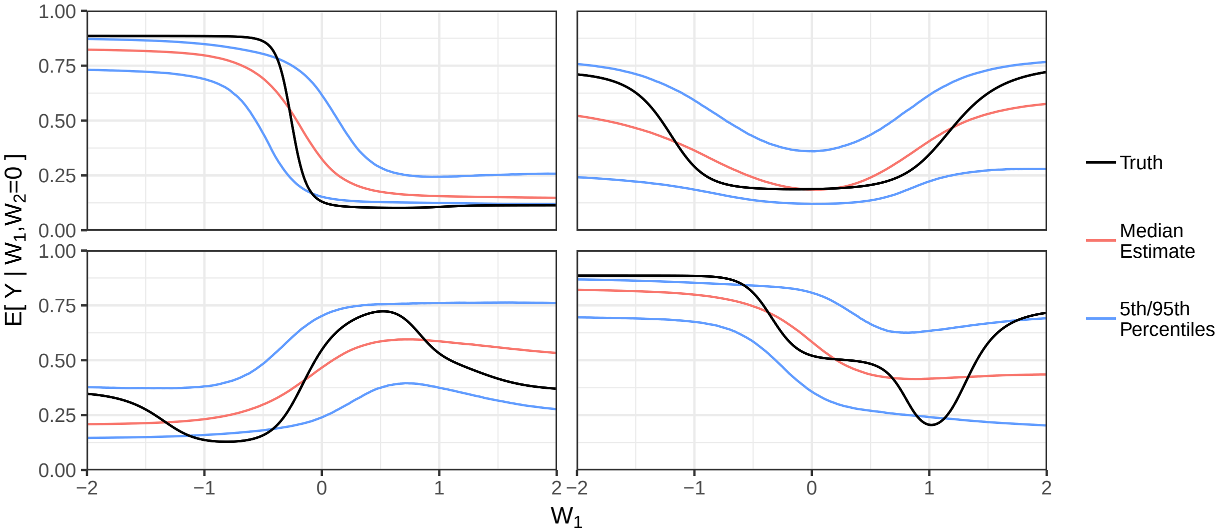

Fig. S2. Pointwise quantiles of the fit of our learned two-layer multilayer perceptron prediction function at $\mathrm{W_2} = 0$ and different values of $\mathrm{W_1}$ based on $\mathbf{n} = 50$ observations from four data-generating distributions. The true regression functions $\mathbb{E}(Y \mid W_1, W_2)$ do not depend on W_2.

Table S2. Final estimated performance of the learned prediction algorithms, the MLE, and a linear-logistic regression. The logistic regression does not use a penalty in the 2-dimensional settings (i, x, xi, xii), and uses cross-validation to select a lasso penalty for the 10-dimensional settings (iv, v, vi, vii, viii, ix). Linear-logistic regressions are not reported in settings ii and iii because they are not generalized linear models.

	Maximal Risk			Uniform Bayes Risk		
Setting	Learned	MLE	Logistic	Learned	MLE	Logistic
i	0.032	0.037	0.036	0.025	0.026	0.031
ii	0.115	0.100		0.039	0.055	
iii	0.199	0.182		0.041	0.057	
iv	0.060	0.073	0.097	0.043	0.060	0.065
v	0.080	0.084	0.095	0.048	0.068	0.062
vi	0.083	0.085	0.098	0.040	0.064	0.055
vii	0.058	0.065	0.088	0.037	0.056	0.060
viii	0.048	0.049	0.072	0.027	0.045	0.051
ix	0.026	0.028	0.044	0.014	0.023	0.037
x	0.020	0.026	0.035	0.016	0.020	0.035
xi	0.025	0.033	0.035	0.020	0.024	0.033
xii	0.031	0.035	0.035	0.024	0.025	0.031

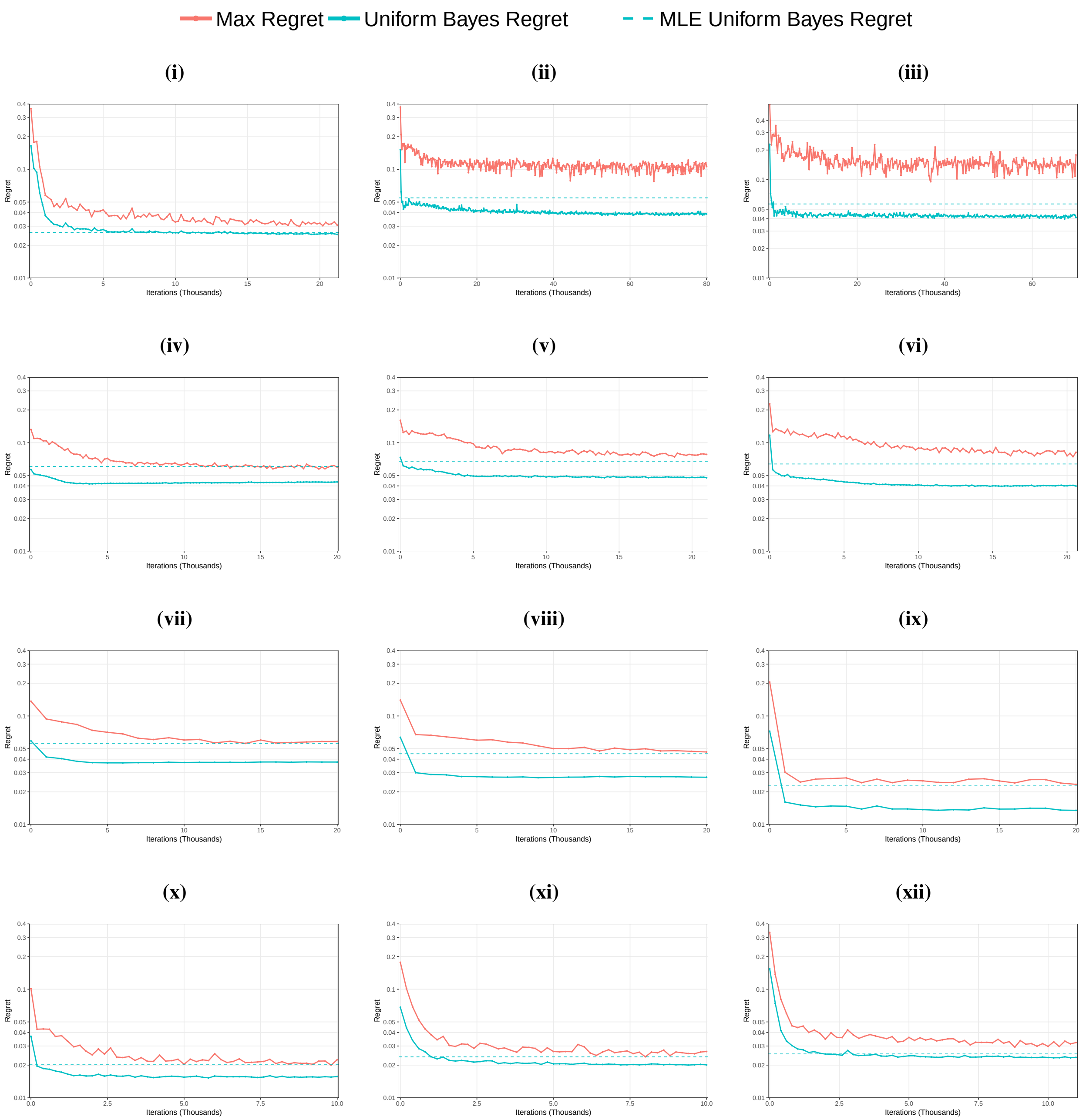

Fig. S3. Learning curves for worst-case (red) and uniform-prior Bayes (blue) prediction performance in the logistic regression Settings (i) through (xii) displayed in Table 1. An iteration corresponds to one random search to identify an unfavorable distribution and two gradient update steps to the procedure network to improve performance at this distribution. For computational reasons, it was not possible to interrogate the estimator with many starts at each iteration. Therefore, the solid red lines should be interpreted as lower bounds for the true maximal risk of the estimator at a given iterations – tighter bounds for the maximal risk of the final selected estimator are available in Table S2, where more starting values were used in the interrogation algorithm.

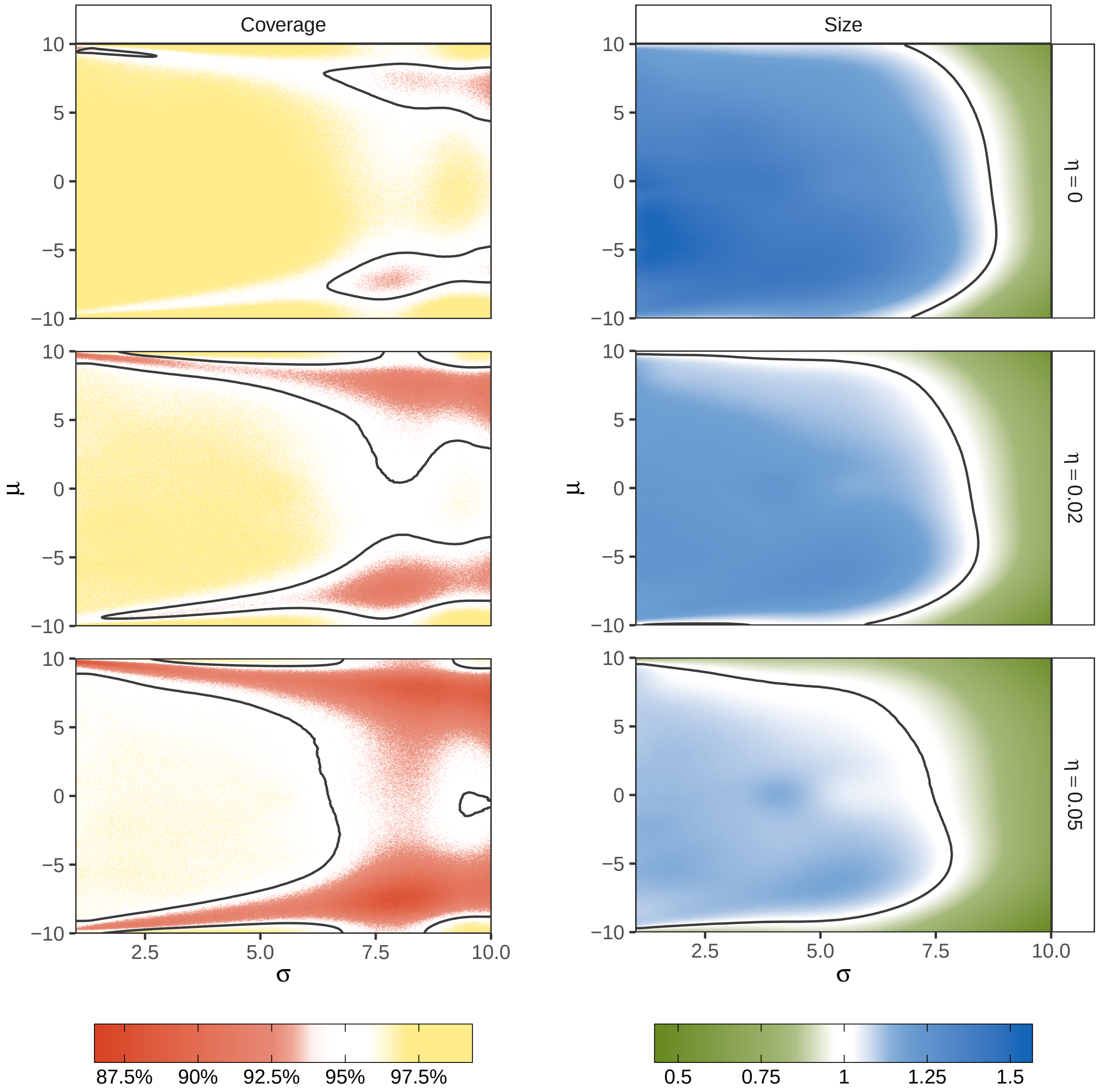

Fig. S4. Performance of the learned 95% level confidence region procedure across different values of η. Contours indicate comparable performance to the reference procedure. Contours are drawn using smoothed estimates of risk, where the smoothing is performed using k-nearest neighbors estimates of coverage ($k = 625$) and size ($k = 100$). Coverage and size both decrease as η grows.

Appendix B. Supplementary tables for data applications.

Table S3. Performance of our learned procedures and of existing procedures in data illustrations. Performance for predicting (**A**) survival on the Titanic based on 2 variables, (**B**) survival on the Titanic based on 10 variables, and (**C**) CD4+ response to the PENNVAX®-B DNA HIV vaccine for our learned procedures and for existing procedures. Larger values indicate better performance for AUC, worse performance for Cross-Entropy. Existing methods are maximum likelihood in all rows except the Lasso Penalty row, which uses a penalized likelihood. Penalized and unpenalized logistic regression predictions are truncated to fall in the interval [0:001; 0:999]. There is minimal Monte Carlo error in the estimates of performance for the Titanic examples in (A) and (B) (95% CIs of width at most 0:004), and so these intervals are not displayed

(A) Predicting Titanic survival based on 2 variables

	Cross-Entropy		AUC	
	Learned	Existing	Learned	Existing
No Hidden Layers (i)	0.663	0.662	0.637	0.649
1 Hidden Layer (ii)	0.664	0.677	0.623	0.636
2 Hidden Layers (iii)	0.663	0.681	0.629	0.631
Logistic, No Penalty		0.686		0.648
Logistic, Lasso Penalty		0.679		0.607

(B) Predicting Titanic survival based on 10 variables

	Cross-Entropy		AUC	
	Learned	Existing	Learned	Existing
Linear, No Intercept (iv)	0.597	0.600	0.760	0.754
Linear, Intercept +/- 1 (v)	0.578	0.577	0.754	0.752
Linear, Intercept +/- 2 (vi)	0.587	0.580	0.757	0.750
Logistic, No Penalty		0.920		0.749
Logistic, Lasso Penalty		0.560		0.782

(C) Predicting CD4+ immune response based on 2 variables

	Cross-Entropy	
	Learned	Existing
Linear, Slopes +/- 0.5 (x)	0.637 (0.570,0.705)	0.644 (0.567,0.721)
Linear, Slopes +/- 1 (xi)	0.637 (0.553,0.722)	0.651 (0.550,0.752)
Linear, Slopes +/- 2 (xii)	0.648 (0.546,0.751)	0.660 (0.553,0.766)
Logistic, No Penalty		0.661 (0.551,0.771)
Logistic, Lasso Penalty		0.669 (0.583,0.756)

	AUC	
	Learned	Existing
Linear, Slopes +/- 0.5 (x)	0.681 (0.544,0.819)	0.686 (0.551,0.821)
Linear, Slopes +/- 1 (xi)	0.686 (0.550,0.822)	0.688 (0.553,0.822)
Linear, Slopes +/- 2 (xii)	0.690 (0.555,0.826)	0.684 (0.550,0.818)
Logistic, No Penalty		0.693 (0.560,0.827)
Logistic, Lasso Penalty		0.633 (0.446,0.821)

Appendix C. Methods for confidence region construction experiments.

The procedure for constructing a $1 - \alpha = 0.95$ level confidence region was learned in two stages. First, a two-dimensional point estimator V of (μ, σ) was learned via an alternating algorithm that optimizes an information-normalized mean-squared error risk (see Eq. S1). We denote the two coordinates of $V(x)$ by $V_1(x)$ and $V_2(x)$. The resulting estimate served as an interior point around which the rectangular confidence region was constructed. The procedure T then takes as input the data and outputs a four-dimensional boundary. The first two coordinates T_1 and T_2 are designed to estimate $t_1(P)$ and $t_2(P)$, which are respectively defined as the $\alpha/4$ and $1 - \alpha/4$ quantiles of the distribution of $\mu - V_1(X)$ when X is drawn from the normal distribution P indexed by μ and σ. Because the interval $[t_1(P), t_2(P)]$ contains μ with probability exactly $1 - \alpha/2$, and we wanted the estimate $[T_1(X), T_2(X)]$ of this interval to contain μ with probability at least $1 - \alpha/2$, we wanted T_1 to overestimate $t_1(P)$ less often than it underestimates $t_1(P)$, and T_2 to underestimate $t_2(P)$ less often than it overestimates $t_2(P)$. Hence, asymmetric risks were used so that the lower bound for μ would be more heavily penalized when overestimated, and the upper bound for μ more heavily penalized when underestimated – we refer the reader to Eq. S4 for an expression for these risks. Analogous risks were employed to obtain estimates T_3 and T_4 of $t_3(P)$ and $t_4(P)$, namely the $\alpha/4$ and $1 - \alpha/4$ quantiles of $\sigma - V_2(X)$, respectively, when $X \sim P$. We used the sum of these four risks to describe the performance of a procedure that outputs the four boundaries. We then iteratively updated a procedure estimating these four quantities to minimize the corresponding Bayes risk against a diffuse prior. As discussed in the section of the main text describing the challenges that arose in our experiments, we learned our interior point procedure and our confidence region procedure over the expanded model specified by $\mu \in [-10, 10]$ and $\sigma \in [0.5, 10]$.

Below, we present the settings used to learn the interior point about which the confidence region is constructed, and then, those used to learn the procedure returning the boundary of the confidence region. Subsequently, we present the method we used to interrogate the learned procedures.

C.1 Meta-learner implementation for interior point

We start by describing the derivation of the point estimator $V : x \mapsto (V_1(x), V_2(x))$ of (μ, σ) that served as the interior point about which our confidence region was constructed. The risk of the procedure V at the distribution P indexed by μ and σ was the information-normalized mean squared error, defined as

$$\mathcal{R}(V, P) = \frac{\mathbb{E}_P[\{V_1(X) - \mu\}^2 + \{V_2(X) - \sigma\}^2]}{\sigma^2} \,. \tag{S1}$$

We used an LSTM network with a forget gate to parameterize our interior point procedure V (21). The estimator network used to define the interior point is displayed in Fig. S7. This network is slightly more complex than the network used in the prediction example. In brief, the network twice sequentially passes the n observations through two LSTM layers. The first n inputs are used to initialize the cell state. The hidden states from the latter n inputs are subsequently passed through a dense layer. The final outputs are passed through a mean pooling layer, yielding an estimate of (μ, σ).

The prior in the alternating algorithm for the interior point procedure was parameterized as a multilayer perceptron generator network with one hidden layer consisting of 25 hidden nodes using activation function $x \mapsto \max\{0.01x, x\}$ and a scaled sigmoid activation function for the output layer. The generator network had two sets of inputs. The first was a two-dimensional noise vector containing independent Normal(0.5,0.25) random variables. The second consisted of six 2-dimensional inputs, each of which corresponded to $[\{V_1(X) - \mu\}^2 + \{V_2(X) - \sigma\}^2]/\sigma^2$ evaluated at a single X drawn from a Normal(μ,σ^2) distribution, where (μ, σ) varied over the four extremes $(\pm 10, 0.5)$ and $(\pm 10, 10)$ of the parameter space, as well as $(\pm 10, 5.25)$. These additional inputs were designed to give the prior network a sense of the performance of the current estimator across the parameter space, which we found helped avoid mode collapse. As we did in the point estimation experiments, we also employed a penalty to avoid mode collapse. In particular, for f defined as $x \mapsto \max\{0.01x, x\}$, we regularized the risk $\mathcal{R}^{\mathrm{B}}(V, \Pi) = \mathbb{E}_\Pi\left[\mathcal{R}(V, P)\right]$ for the prior Π using an estimate of

$$50 \left\{ f(\mathcal{R}^{\mathrm{B}}(V, \Pi^{(1)}) - \mathcal{R}^{\mathrm{B}}(T, \Pi)) + f(\mathcal{R}^{\mathrm{B}}(V, \Pi^{(2)}) - \mathcal{R}^{\mathrm{B}}(T, \Pi)) \right\} \tag{S2}$$

where $\Pi^{(1)}$ draws (μ, σ) uniformly at random from the parameter space, and $\Pi^{(2)}$ draws μ uniformly from the parameter space and independently draws the precision σ^{-2} uniformly from the parameter space. In

practice, we replaced the two instances of $\mathcal{R}^{\mathrm{B}}$ above with Monte Carlo estimates based on 500 independent draws from the prior.

The alternating algorithm made three Adam updates to the procedure V for every one update to the prior. The Adam routine used parameters $\alpha_0 = 1 \times 10^{-3}$ and $\beta_0 = 0.5$ for the prior network, and $\alpha_0 = 8 \times 10^{-4}$ and $\beta_0 = 0.9$ for the procedure network. Each instance of the risk in Eq. S1 was estimated using 500 Monte Carlo draws from a distribution P from the prior, and for each P, one draw of a data set X from P. The interior point procedure was initialized for 300 Adam updates against a fixed prior that draws (μ, σ) uniformly from the parameter space, and the alternating algorithm was subsequently run over 10^5 iterations. The final interior point procedure was selected via two steps. First, the maximum information-normalized risk was approximated at every 50th iteration between iterations 5×10^4 and 10^5 by computing the maximum of 500-replicate Monte Carlo estimates of the risk at 50 (μ, σ) pairs drawn uniformly from the parameter space, the four pairs on the corners of the parameter space, and eight additional points on the boundary of the parameter space, namely two pairs drawn uniformly at random from each of the four edges of the parameter space. Next, the iterations with a maximal risk estimate less than 0.05 were interrogated again using the same strategy, but evaluated instead using 1000 random values of (μ, σ) and 1000 Monte Carlo replicates. The final maximal risk of the learned interior point procedure was similar to the maximal risk of the MLE for (μ, σ).

C.2 Meta-learner implementation for bounds of the confidence region

We start by describing the risk that we used to learn the confidence region procedure $T : x \mapsto (T_j(x) : j = 1, 2, 3, 4)$. The risk relied on a tuning parameter $\eta \in [0, 1)$, and is defined as the sum over $j \in \{1, 2, 3, 4\}$ of risks $\mathcal{R}_{j,\eta}(T_j, P)$, each of which is designed to measure the quality of T_j for estimating $t_j(P)$. We expanded the input of each T_j so that it takes as input both X and η. We defined the risks as

$$\mathcal{R}_{j,\eta}(T_j, P) = \frac{\mathbb{E}_P\left\{[\{T_j(X,\eta) - t_j(P)\}^+]^2 + \eta[\{T_j(X,\eta) - t_j(P)\}^-]^2\right\}}{\sigma^2 \mathcal{R}(V, P)}, \quad j \in \{1, 3\} \tag{S3}$$

$$\mathcal{R}_{j,\eta}(T_j, P) = \frac{\mathbb{E}_P\left\{[\{T_j(X,\eta) - t_j(P)\}^-]^2 + \eta[\{T_j(X,\eta) - t_j(P)\}^+]^2\right\}}{\sigma^2 \mathcal{R}(V, P)}, \quad j \in \{2, 4\} \tag{S4}$$

where $\mathcal{R}(V, P)$ is defined in Eq. S1. The first risk defined above more heavily penalizes overestimation of $t_j(P)$, whereas the second risk more heavily penalizes underestimation of $t_j(P)$. To remove the dependence

of the risk $\mathcal{R}_\eta$ on the tuning parameter η, we define the averaged risk $(T, P) \mapsto \mathbb{E}[\mathcal{R}_\eta(T, P)]$, where the expectation is over $\eta \sim \text{Beta}(1.5, 4)$. We note that the final learned procedure can be interrogated to guide the selection of the parameter η. In particular, η can be chosen as the maximal value at which the procedure achieves an acceptable coverage at all distributions in the model. Choosing η as large as possible helps to minimize the size of the confidence region. In practice, we approximated $\mathbb{E}[\mathcal{R}_{j,\eta}(T_j, P)]$ by replacing $t_j(P)$ with sample quantiles based on 500 data sets randomly drawn from P and by approximating the expectations over η and X using a Monte Carlo approximation based on 500 pairs of $\eta \sim \text{Beta}(1.5, 4)$ and $X \sim P$.

To learn our procedure, we implemented the analogue of our alternating algorithm in the setting where the prior is fixed at the prior Π_u that draws (μ, σ) uniformly from the parameter space. The procedure minimizing the objective function corresponds to the Bayes procedure with respect to the risk $\mathcal{R}$ against this uniform prior. We estimated the Bayes risk by taking Monte Carlo draws of $P \sim \Pi_u$. We gradually increased the total number of distributions drawn from the prior from 20 to 70 over approximately 1.5×10^5 iterations.

We parameterized $\mathcal{T}$ using a similar neural network class to that used to define the interior point. A key difference between the architectures is that each $T \in \mathcal{T}$ takes as input both a data set x and a value of the tuning parameter η. Other differences between the two architectures are outlined in Supplementary Appendix D. The final confidence region procedure was equal to $x \mapsto T(x, \eta)$ at a value of η selected to ensure adequate frequentist coverage. We report the performance procedure at $\eta = 0, 0.02, 0.05$. The Adam algorithm for the LSTM procedure network used parameters $\alpha_0 = 5 \times 10^{-4}$ and $\beta_0 = 0.9$.

C.3 Interrogating the learned confidence region procedures and comparators

To select a final iteration at which to report the performance of our procedure, we performed a shallow interrogation at every 100 iterations from iteration 10^5 until the final iteration. Each of these shallow interrogations performed a random search using 5×10^3 (μ, σ) pairs drawn uniformly from the parameter space. For each pair, the coverage of the procedure with tuning parameter $\eta = 0.05$ was evaluated using 5×10^3 random draws of data sets of size $n = 50$ from a Normal(μ, σ^2) distribution. The iteration with the maximal estimated worst-case coverage across the 5×10^3 pairs was selected as our final iteration to be evaluated in the deep interrogation. Specifically, iteration 1.421×10^5 was selected.

When reporting the final performance of the learned procedures corresponding to $\eta = 0$, 0.02 and 0.05, coverage and expected size were estimated using 5×10^3 Monte Carlo draws at each (μ, σ) pairs in a linearly spaced 500×500 grid on the parameter space. This grid was used to identify the parameter at which the learned procedures' coverage was worst and the parameter at which the expected size was worst. The final reported worst-case coverage and expected size were obtained by evaluating performance at these two worst-case parameters using an additional 5×10^3 Monte Carlo draws.

We compared our learned procedures to a reference procedure that develops a rectangular confidence region based on calculations in (48). In the notation of Eq. 6 in (48), we let $\alpha_1 = \alpha_2$. For a given α_1, we defined the corresponding rectangular confidence region as the smallest rectangle containing the non-rectangular region presented in that paper. One such non-rectangular region is displayed in the shaded portion of Fig. 1 in (48). We then selected α_1 numerically so that the rectangular region had approximately 95% level coverage. Specifically, for a given data realization $x = (x_1, \dots, x_n)$ with $\bar{x}$ denoting the sample mean and $s^2 = n^{-1} \sum_i (x_i - \bar{x})^2$, this comparator region was taken to be of the form

$$\left\{ (\mu, \sigma) : \bar{x} - \frac{as}{\sqrt{b}} < \mu < \bar{x} + \frac{as}{\sqrt{b}}, s\sqrt{\frac{n}{c}} < \sigma < s\sqrt{\frac{n}{b}} \right\} \qquad \text{(S5)}$$

where, for $\alpha_1 = \alpha_2 = 1 - (1 - 0.079)^{1/2}$, a is the upper $\alpha_1/2$ percentile of a standard normal distribution, and b and c are the lower and upper $\alpha_2/2$ percentiles of a chi-squared distribution with $n - 1 = 49$ degrees of freedom, respectively. This region has constant expected (information-normalized) size of approximately 0.315 throughout the statistical model.

Appendix D. Neural network architectures in numerical experiments.

The networks used in our experiments are displayed in Figs. S5 through S7. Fig. S5 displays standard multilayer perceptrons. Figs. S6 and S7 display LSTMs that induce an ordering on the observations and then combine the hidden states via a mean pooling layer. For clarity, we describe the more complex of these two LSTM architectures here.

The network in Fig. S7 network can be evaluated in three steps. In the first step, a two-layer LSTM with a forget gate is used (21). For each layer $j = 1, 2$ we initialize the cell state $C_0^{(j)}$ and history state $H_0^{(j)}$ to 50×50 matrices containing independent Normal(0,10^{-4}) random variables. At each time $i = 1, 2, \dots, 2n$,

the first layer of the LSTM takes as input the data point $X_{(i \mod n)}$, the previous cell state $C_{i-1}^{(1)}$, and the previous history state $H_{i-1}^{(1)}$, and outputs a cell state $C_i^{(1)}$ and a history state $H_i^{(1)}$. The second layer takes as input the first layer's history state $H_i^{(1)}$, the previous cell state $C_{i-1}^{(2)}$, and the previous history state $H_{i-1}^{(2)}$, and outputs a cell state $C_i^{(2)}$ and a history state $H_i^{(2)}$. The second step uses a multilayer perceptron M with one hidden layer containing 50 leaky rectified linear units and an output layer of dimension 2 equipped with the identity activation function. For $i = n+1, n+2, \ldots, 2n$, each history state $H_i^{(2)}$ is passed through this multilayer perceptron to yield $M(H_i^{(2)})$. The third step performs a mean pooling, yielding the estimate $\frac{1}{n}\sum_{i=1}^{n} M(H_i^{(2)})$ of (μ, σ). Note that the first n history states were not used after Step 1: we call these states "burn-in" states, which we use to initialize the cell and history states but which we do not use in Steps 2 or 3.

The network defining the boundaries of the confidence region is an LSTM network that is identical to the network used for the interior point procedure V displayed in Fig. S7 except that each cell and hidden state is a 100×100 matrix rather than a 50×50 matrix, the hidden layer in the multilayer perceptron that processes the history states from the second LSTM layer has 100 hidden nodes, the output is 4-dimensional rather than 2-dimensional, and for each input $i = 1, 2, \ldots, 2n$ the data point $X_{(i \mod n)}$ *and* the value of η are supplied as inputs.

Appendix E. Further discussion of guarantees for nested minimax algorithms.

We now extend the discussion of guarantees for nested minimax algorithms given the main text. First, we give convergence guarantees in the setting that the risk functional is nonconvex. Second, we describe an appealing choice of dominating measure that can be used in the iterative algorithm presented in the Theoretical Guarantees section of the main text. We close with proofs of results presented in this section.

E.1 Convergence of approximate generalized gradient descent for nonconvex risk functionals

Recall that Lemma 2 from the main text established that $g_k \equiv g(S_k, P_k)$ is an approximate subgradient of $\mathcal{R}^\star$ at S_k when the risk $\mathcal{R}$ is convex and P_k is unfavorable. In this section, we study the performance of the

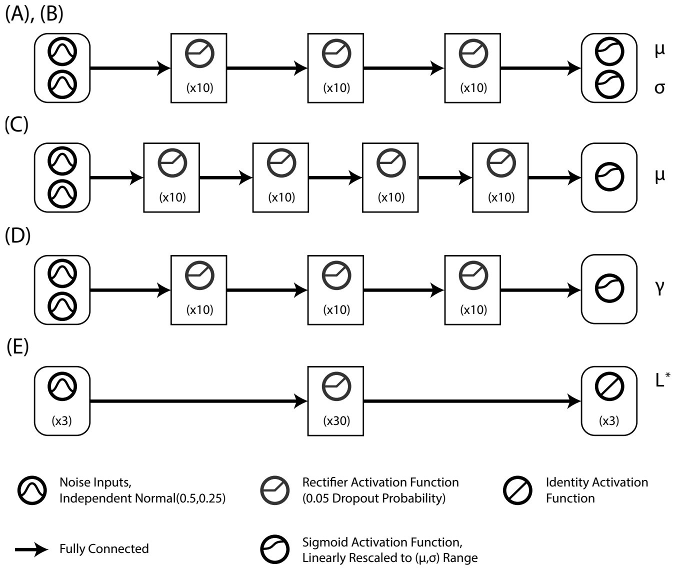

Above: Prior generator networks

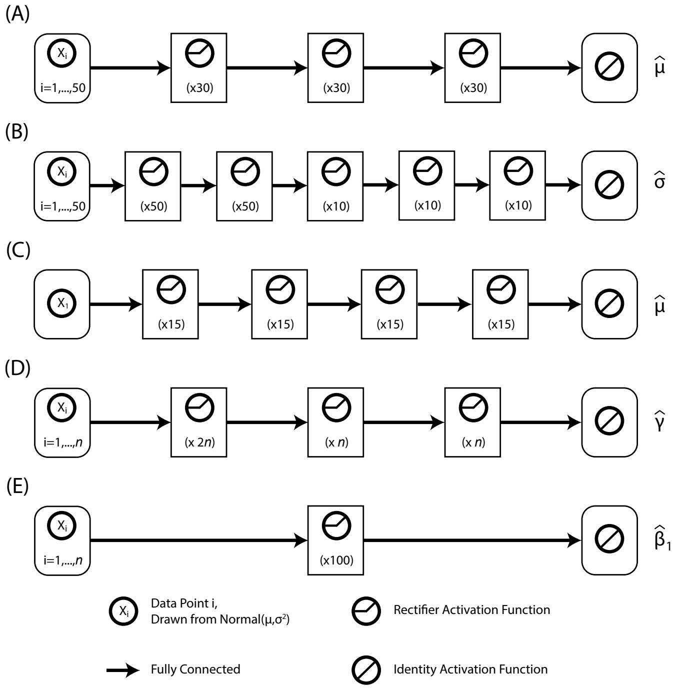

Above: Procedure networks

Fig. S5. Prior generator multilayer perceptrons and procedure multilayer perceptrons used for the point estimation examples. These networks were used when estimating **(A)** the mean μ of a Gaussian based on $n = 50$ observations; **(B)** the standard deviation σ of a Gaussian based on $n = 50$ observations; **(C)** the mean μ of a Gaussian with $n = 1$ observations; **(D)** the univariate parameter γ indexing a uniform mixture between a Normal$(0, 1)$ and a Normal$(\gamma, \exp\{-2\gamma^{-2}\})$ distribution based on n observations; **(E)** the first regression coefficient β_1 indexing a two-dimensional linear model. * In setting (E), the two-dimensional regression coefficient $\beta = (\beta_1, \beta_2)$ is derived from the output $L = (L_1, L_2, L_3)$ of the generator network by setting $\beta_j = 10\frac{L_j}{(L_1^2+L_2^2+L_3^2)^{1/2}}$, $j = 1, 2$. This ensures that β falls within the closed ℓ_2 sphere that is centered at the origin and has radius 10, which in turn ensures that the distribution for the outcome Y falls in the model.

iterative update scheme

$$S_{k+1} : (x,a) \mapsto S_k(x,a) - \zeta_k g_k(x,a) \tag{S6}$$

when the risk is not convex, that is, without condition A2 from the main text. In the nonconvex case, we refer to g_k as an (approximate) generalized gradient rather than an (approximate) subgradient (46). Note that, unlike in the main text, here we are focusing on the case that a deterministic generalized gradient is available at each step. We now prove that, under some conditions, our generalized gradient descent algorithm converges to the nearest local minimum. Following (49), we suspect that, at the cost of longer proofs, similar results can be established for a stochastic version of this algorithm by establishing almost sure convergence results via martingale techniques.

Three key results are presented in this subsection. All of these results rely on there existing a subset $\bar{\mathcal{S}}$ of $\mathcal{S}$ that satisfies several upcoming conditions. The first result, Lemma S2, implies that, given a starting point in the interior of $\bar{\mathcal{S}}$ and a sufficiently small ζ_1, our algorithm will yield procedures S_k that remain in $\bar{\mathcal{S}}$ for all steps k. The second result, Lemma S3, shows that our learned procedure will converge to the set $\bar{\mathcal{S}}^\star \equiv \{S \in \bar{\mathcal{S}} : \mathcal{R}^\star(S) = \inf_{\bar{S}\in\bar{\mathcal{S}}} \mathcal{R}^\star(\bar{S})\}$ of procedures that obtain the optimal performance within $\bar{\mathcal{S}}$. The third result, Theorem S4, shows that, under an additional condition of local convexity near $\bar{\mathcal{S}}^\star$, the risk of our algorithm converges to the risk of $\bar{\mathcal{S}}^\star$ at the same polynomial rate guaranteed in Theorem 1 for the convex case. As a consequence, we will have shown that the maximal risk of our procedure after K steps shrinks to $\inf_{\bar{S}\in\bar{\mathcal{S}}} \mathcal{R}^\star(\bar{S})$ at rate $O(\log K/K^{1/2})$ if $\zeta_k = Ck^{-1/2}$ for some $C > 0$.

Before presenting our key results, we give a preliminary lemma. This lemma relies on the below condition on $\bar{\mathcal{S}}$.

SA1) The set $\bar{\mathcal{S}}$ is compact.

Lemma S1. *If conditions A1 and SA1 hold, then $\bar{\mathcal{S}}^\star$ is compact and nonempty.*

The proof of Lemma S1 is given in Section E.3. We also require that $\bar{\mathcal{S}}^\star$ satisfy the following condition.

SA2) $\bar{\mathcal{S}}^\star$ falls in the interior of $\bar{\mathcal{S}}$.

Define the distance between a procedure S and the set $\mathcal{S}^\star$ as $\rho(S,\mathcal{S}^\star) \equiv \inf_{S^\star\in\mathcal{S}^\star} \|S - S^\star\|$. For all $\delta > 0$, we define $\mathcal{B}(\bar{\mathcal{S}}^\star,\delta) \equiv \{S \in \mathcal{S} : \rho(S,\bar{\mathcal{S}}^\star) < \delta\}$ as the collection of procedures that fall within distance δ of $\bar{\mathcal{S}}^\star$. We require the following condition:

SA3) For each sufficiently small $\epsilon \geq 0$ and $\delta > 0$, the following holds for all $\bar{S}^\star \in \bar{\mathcal{S}}^\star$

$$c(\delta,\epsilon) \equiv \inf_{S\in\bar{\mathcal{S}}\cap\mathcal{B}(\bar{\mathcal{S}}^\star,\delta)^C} \inf_{P\in\mathcal{P}:\mathcal{R}^\star(S)\leq\mathcal{R}(S,P)+\epsilon} \left[\mathcal{R}(S,P) - \mathcal{R}(\bar{S}^\star,P) + \mathrm{Rem}(S,\bar{S}^\star;P)\right] > 0$$

We now argue that the above is implied by the local convexity condition that

$$\tilde{c}(\delta,\epsilon) \equiv \inf_{S\in\bar{\mathcal{S}}\cap\mathcal{B}(\bar{\mathcal{S}}^\star,\delta)^C} \inf_{P\in\mathcal{P}:\mathcal{R}^\star(S)\leq\mathcal{R}(S,P)+\epsilon} \mathrm{Rem}(S,\bar{S}^\star;P) > 0 \text{ for all } \epsilon,\delta \text{ sufficiently small} \tag{S7}$$

Fix $\epsilon \geq 0$ and $\delta > 0$ small enough so that the above holds. For $S \in \bar{\mathcal{S}}\cap\mathcal{B}(\bar{\mathcal{S}}^\star,\delta)^C$ and $\bar{S}^\star \in \bar{\mathcal{S}}^\star$, it is true that $\mathcal{R}(\bar{S}^\star,P) \leq \mathcal{R}^\star(\bar{S}^\star) \leq \mathcal{R}^\star(S)$. Hence, if P satisfies $\mathcal{R}^\star(S) \leq \mathcal{R}(S,P)+\epsilon$, then $\mathcal{R}(S,P) - \mathcal{R}(\bar{S}^\star,P) \geq -\epsilon$. Consequently, $c(\delta,\epsilon) \geq \tilde{c}(\delta,\epsilon) - \epsilon$. If Eq. S7 holds, it follows that $c(\delta,\epsilon) > 0$ for all $\epsilon \in [0,\tilde{c}(\delta,\epsilon)/2]$, that is, condition SA3 holds. Hence, condition SA3 is implied by (S7). We note that the converse implication does not hold: condition SA3 can hold even if (S7) fails to hold.

To ensure that we can invoke condition SA3, we will assume that the error of the interrogation shrinks as the number of steps increases.

SA4) ϵ_k is nonincreasing and converges to zero as $k \to \infty$.

Finally, we assume that our procedure is close to the optimizer eventually, and furthermore that the step size is not too large when this occurs.

SA5) For all $\delta > 0$ and $\epsilon \geq 0$ small enough and $\tau > 0$ small enough so that $\mathcal{B}(\bar{\mathcal{S}}^\star,\delta+\tau) \subset \bar{\mathcal{S}}$, there exists a k_0 (that may rely on δ,ϵ,τ) with $\sup_{k\geq k_0} \zeta_k < \min\{2c(\delta,\epsilon_{k_0})M^{-1}, \tau M^{-1/2}\}$, $S_{k_0} \in \mathcal{B}(\bar{\mathcal{S}}^\star,\delta+\tau)$, and $\epsilon_{k_0} \leq \epsilon$.

The following lemma gives conditions under which the procedure sequence $\{S_k\}$ remains in a neighborhood of $\bar{\mathcal{S}}^\star$ for all k after the first time point k_0 at which S_{k_0} is in this neighborhood and both ζ_{k_0} and ϵ_{k_0} are small enough. If $k_0 = 1$, then this gives conditions under which our procedure sequence will always remain in $\bar{\mathcal{S}}$, and we will later invoke this lemma to argue that our procedure sequence will eventually enter and remain

in a neighborhood of $\bar{\mathcal{S}}^\star$ in which the maximal risk functional is convex, thereby allowing us to obtain a fast rate of convergence for the maximal risk of the best procedure in our sequence.

Lemma S2 (Estimator remains in neighborhood of local minimizer)**.** *Fix an initial procedure $S_1 \in \bar{\mathcal{S}}$, and recursively define the sequence $\{S_k \in \mathcal{S}\}$ by Eq. S6. Suppose that conditions A1, A4, SA1, SA2, SA3, SA4, and SA5 hold, where $\delta > 0$, $\epsilon \geq 0$, and $\tau > 0$ are chosen so that the conditions of SA4 are satisfied. Then, $S_k \in \mathcal{B}(\bar{\mathcal{S}}^\star, \delta + \tau)$ for all $k \geq k_0$.*

The proof of Lemma S2 is given in Section E.3. The proof of this result and the proof of the upcoming lemma follow arguments given in (49), but modify these arguments to apply in our setting where the interrogation may not achieve the maximal risk and so that the optimization routine does not *a priori* restrict all steps to fall in $\bar{\mathcal{S}}$.

We now show that our procedure converges to a local minimizer of the maximal risk.

Lemma S3 (Estimator converges to a local minimum)**.** *Under the conditions of Lemma S2 and also condition A6, $\rho(S_k, \bar{\mathcal{S}}^\star) \to 0$ as $k \to \infty$.*

The proof of Lemma S3 is given in Section E.3. As a consequence of the above and the continuity of $\mathcal{R}^\star$ on $\mathcal{S}$ under condition A1, $\mathcal{R}^\star(S_k) \to \inf_{\bar{S} \in \bar{\mathcal{S}}} \mathcal{R}^\star(\bar{S})$ as $k \to \infty$. We close this section by noting that this maximal risk convergence occurs at a polynomial rate under the following additional local convexity condition, which represents a weakening of condition A2.

SA6) There exists a neighborhood $\bar{\mathcal{S}}_1 \subset \bar{\mathcal{S}}$ of $\bar{\mathcal{S}}^\star$ within which it holds that $S \mapsto \mathcal{R}(S, P)$ is convex for each $P \in \mathcal{P}$, that is

$$\mathcal{R}([1-\epsilon]S + \epsilon\tilde{S}, P) \leq (1-\epsilon)\mathcal{R}(S, P) + \epsilon\mathcal{R}(\tilde{S}, P) \text{ for all } \epsilon \text{ in } [0,1] \text{ and } S, \tilde{S} \in \bar{\mathcal{S}}_1$$

Theorem S4 (Maximal risk quickly converges to the locally optimal maximal risk)**.** *Under the conditions of Lemma S3 and the additional local convexity condition SA6, there exists some natural number K_1 for which the following inequality holds for all $K \geq K_1$:*

$$\min_{k=1,\ldots,K} \mathcal{R}^\star(S_k) - \inf_{S \in \mathcal{S}} \mathcal{R}^\star(S) \leq \frac{\rho(S_{K_1}, \bar{\mathcal{S}}^\star)^2 + \sum_{k=K_1}^K \zeta_k(M\zeta_k + 2\epsilon_k)}{2\sum_{k=K_1}^K \zeta_k}.$$

If $\zeta_k = \Theta(k^{-1/2})$ and $\epsilon_k = O(k^{-1/2})$, then the right-hand side is $O(\log K / K^{1/2})$.

The proof of this result is omitted, but it consists of first invoking Lemma S3 to identify a valid choice of K_1 from the proof statement, and subsequently applying nearly identical arguments to those used to prove Theorem 1.

E.2 An appealing choice of dominating measure

In this subsection, we propose a choice of the dominating measure ν. For a rich class of statistical models, we show that this measure satisfies the desirable properties described in the main text. In particular, it is finite, dominates all distributions in $\mathcal{P}$, and satisfies $\sup_{P,x} \frac{dP}{d\nu}(x) \leq 1 < \infty$.

Here we work on a complete measure space $(\mathcal{X}, \Sigma, \lambda)$, where $\mathcal{X}$ is a set, Σ is a σ-algebra on $\mathcal{X}$, and λ is a σ-finite measure on $(\mathcal{X}, \Sigma)$. We will suppose that X is drawn from a distribution in the model $\mathcal{P} \equiv \{P_\gamma : \gamma \in \Gamma\}$, where Γ is a subset of a separable metric space $(\mathcal{G}, \mathfrak{d})$ and each $P_\gamma \in \mathcal{P}$ is mutually absolutely continuous with respect to λ. A consequence of this mutual absolute continuity property is that all distributions in $\mathcal{P}$ have the same support, namely $\mathcal{X}$. We require this model to satisfy two conditions. The first, condition SA7, is a requirement on the smoothness of $\gamma \mapsto \log \frac{dP_\gamma}{d\lambda}(x)$ across all $x \in \mathcal{X}$.

SA7) There is a measurable function $r : \mathcal{X} \to [0, \infty)$ for which:

(i) There exists an $\varepsilon > 0$ such that $\int_{\mathcal{X}} \exp[\varepsilon r(x)] \, dP_\gamma(x) < \infty$ for all $\gamma \in \Gamma$.

(ii) There exists a $q > 0$ such that, for all $\gamma_1, \gamma_2 \in \Gamma$ and λ-almost all $x \in \mathcal{X}$,

$$\left| \log \frac{dP_{\gamma_1}}{d\lambda}(x) - \log \frac{dP_{\gamma_2}}{d\lambda}(x) \right| \leq \mathfrak{d}(\gamma_1, \gamma_2)^q r(x).$$

The second condition, which is stated below, restricts the size of the set Γ that indexes distributions in $\mathcal{P}$.

SA8) The set Γ is totally bounded in $(\mathcal{G}, \mathfrak{d})$, that is, for every $\epsilon > 0$, there exists a finite collection of open balls of radius ϵ that covers Γ.

A simple example of a family that satisfies conditions SA7 and SA8 is the exponential family

$$\left\{ P_\gamma : \frac{dP_\gamma}{d\lambda}(x) = \exp\left[\gamma^\top L(x) - A(\gamma)\right], \gamma \in \Gamma \right\}$$

where γ is the natural parameter, Γ is a compact subset of the open natural parameter space $\Gamma_1 \subseteq \mathbb{R}^{d_1}$, L is the sufficient statistic mapping from $\mathcal{X} = \mathbb{R}^{d_2}$ to $\mathbb{R}^{d_1}$, and $A : \Gamma \to \mathbb{R}$ is the log-partition function.

Condition SA8 holds by the compactness of Γ. If, as is true for commonly used exponential families, the log-partition function is Lipschitz continuous on all compact subsets of the natural parameter space, then it can be shown that condition SA7 is satisfied by $q = 1$, some $\varepsilon > 0$, and r equal to the function $x \mapsto L(x) + c$, where c is the Lipschitz constant of A on Γ. We note that conditions SA7 and SA8 are also satisfied by many models that are not exponential families, including by some semi- and non-parametric models, that is, models in which the indexing set Γ is not finite-dimensional.

In a moment, we will define our proposed dominating measure $\nu^\dagger$. The definition of this dominating measure will rely on the measurability of a function $h^\dagger$ that we will present in the next display. For a given r, ε, and q satisfying the conditions of SA7, let $\mathcal{X}_0$ denote the set of $x \in \mathcal{X}$ for which the display in part ii of condition SA7 does not hold. We define

$$h^\dagger : x \mapsto \begin{cases} \sup_{\gamma \in \Gamma} \frac{dP_\gamma}{d\lambda}(x), & \text{if } x \notin \mathcal{X}_0, \\ 0, & \text{otherwise} \end{cases} \tag{S8}$$

We now argue that $h^\dagger$ is measurable. The argument is based on the following observations. First, the separability of $\mathcal{G}$ implies that there exists a countable dense subset $\mathcal{G}_1$ of $\mathcal{G}$. Second, suprema over countable sets are measurable, and therefore $x \mapsto \sup_{\gamma \in \Gamma \cap \mathcal{G}_1} \frac{dP_\gamma}{d\lambda}(x)$ is measurable. Third, products of measurable functions are measurable, and therefore $x \mapsto I\{x \notin \mathcal{X}_0\} \sup_{\gamma \in \Gamma \cap \mathcal{G}_1} \frac{dP_\gamma}{d\lambda}(x)$ is measurable. Fourth, condition SA7 and the fact that $\mathcal{G}_1$ is dense in $\mathcal{G}$ imply that $\sup_{\gamma \in \Gamma} \frac{dP_\gamma}{d\lambda}(x) = \sup_{\gamma \in \Gamma \cap \mathcal{G}_1} \frac{dP_\gamma}{d\lambda}(x)$ for all $x \notin \mathcal{X}_0$. Combining the third and fourth observations shows that $h^\dagger$ is measurable.

We now define our proposed dominating measure. For a set $B \in \Sigma$, we define

$$\nu^\dagger(B) \equiv \int_B h^\dagger(x) d\lambda(x)$$

The proof of the below result is given in Section E.3.

Theorem S5. *If conditions SA7 and SA8, then $\nu^\dagger$ is a finite measure on the measurable space $(\mathcal{X}, \Sigma)$.*

In light of the above theorem and the fact that $h^\dagger(x)$ is (λ-almost everywhere) equal to the maximum likelihood based on a data set x, we refer to $\nu^\dagger$ as the *maximum likelihood measure*. Noting that $\frac{dP_\gamma}{d\nu^\dagger}(x) \leq 1$ for all x and $\gamma \in \Gamma$, we see that, if ν is set equal to $\nu^\dagger$, then Eq. 19 from the main text is satisfied with $M_1 = 1$.

E.3 Proofs

Proof of Lemma S1. To see that $\bar{\mathcal{S}}^\star$ is nonempty, note that condition A1 implies the $\mathcal{S}$ continuity of $S \mapsto \mathcal{R}(S, P)$ for each $P \in \mathcal{P}$, and composing this functional with a supremum over $P \in \mathcal{P}$ (another continuous operation), implies that $\mathcal{R}^\star$ is continuous in $\mathcal{S}$. As a continuous functional admits and achieves a minimum over a compact set, $\bar{\mathcal{S}}^\star$ is nonempty.

We argue by contradiction that $\bar{\mathcal{S}}^\star$ is closed. Suppose $\bar{\mathcal{S}}^\star$ is not closed. Then, $\bar{\mathcal{S}}^\star$ does not contain at least one of its limit points. Take a sequence of elements in $\bar{\mathcal{S}}^\star$ converging to this limit point. By the definition of $\bar{\mathcal{S}}^\star$, the maximal risk is constant along this sequence. By the continuity of $\mathcal{R}^\star$, this implies that the limit of this sequence is contained in $\bar{\mathcal{S}}^\star$, contradiction. The proof concludes by noting that a closed subset of a compact set is compact. □

Proof of Lemma S2. We give proof by induction of the fact that $S_k \in \mathcal{B}(\bar{\mathcal{S}}^\star, \delta + \tau)$ for all $k \geq k_0$. For the base case, we have that $S_{k_0} \in \mathcal{B}(\bar{\mathcal{S}}^\star, \delta + \tau)$ by condition SA5. Fix $k \geq k_0$ and suppose the inductive hypothesis that $S_k \in \mathcal{B}(\bar{\mathcal{S}}^\star, \delta + \tau)$. The remainder of the proof shows that $S_{k+1} \in \mathcal{B}(\bar{\mathcal{S}}^\star, \delta + \tau)$. We consider two cases. In the first case, $S_k \in \mathcal{B}(\bar{\mathcal{S}}^\star, \delta)$. In this case,

$$\rho(S_{k+1}, \bar{\mathcal{S}}^\star) \leq \rho(S_k, \bar{\mathcal{S}}^\star) + \zeta_k \|g_k\| < \delta + M^{1/2}\zeta_k$$

Because condition SA5 implies that $\zeta_k \leq \tau M^{-1/2}$, the right-hand side is less than $\delta + \tau$ and $S_{k+1} \in \mathcal{B}(\bar{\mathcal{S}}^\star, \delta + \tau)$.

In the second case, $S_k \notin \mathcal{B}(\bar{\mathcal{S}}^\star, \delta)$. By Lemma S1, $\bar{\mathcal{S}}^\star$ is compact and nonempty. Because $\bar{S}^\star \mapsto \|S_k - \bar{S}^\star\|$ is continuous in $\mathcal{S}$, this map admits and achieves a minimum over $\bar{\mathcal{S}}^\star$, that is, there exists an $\bar{S}^\star$ satisfying $\|S_k - \bar{S}^\star\| = \rho(S_k, \bar{\mathcal{S}}^\star)$. We have that

$$\|S_{k+1} - \bar{S}^\star\|^2 = \rho(S_k, \bar{\mathcal{S}}^\star)^2 - 2\zeta_k \langle g_k, S_k - \bar{S}^\star \rangle + \zeta_k^2 \|g_k\|^2 \tag{S9}$$

$$\leq \rho(S_k, \bar{\mathcal{S}}^\star)^2 + \zeta_k[\zeta_k M - 2c(\delta, \epsilon)] \tag{S10}$$

where the inequality used condition A4, $\epsilon_{k_0} \leq \epsilon$ by condition SA5, $\epsilon_k \leq \epsilon$ for all $k \geq k_0$ by condition SA4, and the definition of $c(\delta, \epsilon)$ from condition SA3 combined with the definition in Eq. 22. By the inductive hypothesis, the right-hand side is no more than than $\delta + \tau + \zeta_k[\zeta_k M - 2c(\delta, \epsilon)]$. Furthermore, the left-

hand side is lower bounded by $\rho(S_{k+1}, \bar{\mathcal{S}}^\star)^2$. Hence, $\rho(S_{k+1}, \bar{\mathcal{S}}^\star)^2 \leq \delta + \tau + \zeta_k[\zeta_k M - 2c(\delta, \epsilon)]$. By condition SA5, $\zeta_k < 2c(\delta, \epsilon)/M$. Hence, $\zeta_k[\zeta_k M - 2c(\delta, \epsilon)]$ is negative and $S_{k+1} \in \mathcal{B}(\bar{\mathcal{S}}^\star, \delta + \tau)$. □

Proof of Lemma S3. Fix a small $\tilde{\delta} > 0$. We first argue by contradiction that

$$\text{there exists a sequence } \{k_j\} \text{ satisfying } \rho(S_{k_j}, \bar{\mathcal{S}}^\star) < \tilde{\delta} \text{ for all natural numbers } j \tag{S11}$$

Suppose Eq. S11 is false. Then, for some natural number K, $\rho(S_k, \bar{\mathcal{S}}^\star) \geq \tilde{\delta}$ for all $k \geq K$. We can assume without loss of generality that K is at least as large as k_0 from condition SA5, so that, by Lemma S2, $S_K \in \bar{\mathcal{S}}$ for all $k \geq K$. Because condition SA4 enforces that $\epsilon_k \to 0$, we can also assume without loss of generality that K is large enough and $\tilde{\delta}$ is small enough so that the implication of condition SA3 holds, namely that $c(\tilde{\delta}, \epsilon_K) > 0$. As condition A6 ensures that $\zeta_k \to 0$ as $k \to \infty$, we can also assume without loss of generality that K is large enough so that $\zeta_k \leq c(\tilde{\delta}, \epsilon_K)/M$ for all $k \geq K$.

Fix $k \geq K$. As in the proof of Lemma S2, there exists a $\bar{S}^\star \in \bar{\mathcal{S}}^\star$ satisfying $\|S_k - \bar{S}^\star\| = \rho(S_k, \bar{\mathcal{S}}^\star)$. Using the fact that $c(\tilde{\delta}, \epsilon)$ is nondecreasing in ϵ and that $\{\epsilon_k\}$ is a decreasing sequence, we see that similar arguments used to obtain Eq. S10 yield that, for each $k \geq K$

$$\|S_{k+1} - \bar{S}^\star\|^2 \leq \rho(S_k, \bar{\mathcal{S}}^\star)^2 + \zeta_k[\zeta_k M - 2c(\tilde{\delta}, \epsilon_K)]$$

The left-hand side is lower bounded by $\rho(S_{k+1}, \bar{\mathcal{S}}^\star)^2$. As $\zeta_k \leq c(\tilde{\delta}, \epsilon_K)/M$

$$\rho(S_{k+1}, \bar{\mathcal{S}}^\star)^2 \leq \rho(S_k, \bar{\mathcal{S}}^\star)^2 - c(\tilde{\delta}, \epsilon_K)\zeta_k$$

An induction argument in k shows that, for each $K_1 > K$

$$\rho(S_{K_1+1}, \bar{\mathcal{S}}^\star)^2 \leq \rho(S_K, \bar{\mathcal{S}}^\star)^2 - c(\tilde{\delta}, \epsilon_K) \sum_{k=K}^{K_1} \zeta_k$$

By condition A6, the right-hand side diverges to $-\infty$ as $K_1 \to \infty$. But, this is a contradiction as this implies that the left-hand side is upper bounded by a negative number, which contradicts the fact that $\rho(S_{K_1+1}, \bar{\mathcal{S}}^\star) \geq 0$. Hence, Eq. S11 holds.

We now use Eq. S11 to show that $\lim_{k \to 0} \rho(S_k, \bar{\mathcal{S}}^\star) = 0$. Fix $\epsilon > 0$ small enough so that condition SA3 applies with $\delta = \tilde{\delta}$ (if no such ϵ exists, then choose a smaller $\tilde{\delta}$). One can show that there exists a k_j in the subsequence $\{k_j\}$ that is sufficiently large so that $\epsilon_{k_j} < \epsilon$ and $k_0 = k_j$ satisfies the conditions

of SA5 for some $\tau < \tilde{\delta}$, $\delta = \tilde{\delta}$, and ϵ as fixed at the beginning of this paragraph. Hence, by Lemma S2, $S_k \in \mathcal{B}(\bar{\mathcal{S}}^\star, \tilde{\delta}+\tau) \subseteq \mathcal{B}(\bar{\mathcal{S}}^\star, 2\tilde{\delta})$ for all $k \geq k_j$. As the small chosen $\tilde{\delta} > 0$ was arbitrary, $\lim_{k\to 0} \rho(S_k, \bar{\mathcal{S}}^\star) = 0$. □

Proof of Theorem S5. It is straightforward to show that $\nu^\dagger$ is a measure, and so here we only prove that $\nu^\dagger$ is finite.

In this proof, we will use $\mathcal{B}(g, \epsilon)$ to denote an open ball in $(\mathcal{G}, \mathfrak{d})$ of radius $\epsilon > 0$ centered at $g \in \mathcal{G}$. Throughout we let $r : \mathcal{X} \to [0, \infty)$, $\varepsilon > 0$, and $q > 0$ the quantities guaranteed to exist by condition SA7 that were used to define the set $\mathcal{X}_0$ above Eq. S8. To ease notation, we let $\bar{\varepsilon} \equiv \varepsilon^{1/q}$. By condition SA8, there exists a collection of $N < \infty$ open balls in $\mathcal{G}$ of radius $\bar{\varepsilon}/2$ that covers Γ. Denote these balls by $\mathcal{B}(g_1, \bar{\varepsilon}/2), \ldots, \mathcal{B}(g_N, \bar{\varepsilon}/2)$. For each $j \in \{1, \ldots, N\}$, let γ_j denote an element of $\mathcal{B}(g_j, \bar{\varepsilon}/2) \cap \Gamma$.

We now argue that $\mathcal{B}(\gamma_1, \bar{\varepsilon}), \ldots, \mathcal{B}(\gamma_N, \bar{\varepsilon})$ covers Γ. Fix $\gamma \in \Gamma$. Because $\mathcal{B}(g_1, \bar{\varepsilon}/2), \ldots, \mathcal{B}(g_N, \bar{\varepsilon}/2)$ covers Γ, there exists a $j \in \{1, \ldots, N\}$ so that $\gamma \in \mathcal{B}(g_j, \bar{\varepsilon}/2)$. By the triangle inequality, $\mathfrak{d}(\gamma, \gamma_j) \leq \mathfrak{d}(\gamma, g_j) + \mathfrak{d}(g_j, \gamma_j) < \bar{\varepsilon}/2 + \bar{\varepsilon}/2 = \bar{\varepsilon}$. Hence, $\gamma \in \mathcal{B}(\gamma_j, \bar{\varepsilon})$. As $\gamma \in \Gamma$ was arbitrary, $\mathcal{B}(\gamma_1, \bar{\varepsilon}), \ldots, \mathcal{B}(\gamma_N, \bar{\varepsilon})$ covers Γ.

For each $\gamma \in \Gamma$, let $s(\gamma)$ denote an element γ_j of $\{\gamma_1, \ldots, \gamma_N\}$ for which $\gamma \in \mathcal{B}(\gamma_j, \bar{\varepsilon})$. By the triangle inequality and condition SA7, for all $x \notin \mathcal{X}_0$ it holds that

$$\begin{aligned}
h^\dagger(x) &= \sup_{\gamma\in\Gamma} \frac{dP_\gamma}{d\lambda}(x) = \exp\left\{\sup_{\gamma\in\Gamma} \log \frac{dP_\gamma}{d\lambda}(x)\right\} \\
&\leq \exp\left\{\sup_{\gamma\in\Gamma} \log \frac{dP_{s(\gamma)}}{d\lambda}(x) + \sup_{\gamma\in\Gamma} \left|\log \frac{dP_\gamma}{d\lambda}(x) - \log \frac{dP_{s(\gamma)}}{d\lambda}(x)\right|\right\} \\
&\leq \exp\left\{\sup_{\gamma\in\Gamma} \log \frac{dP_{s(\gamma)}}{d\lambda}(x) + \bar{\varepsilon}^q r(x)\right\}
\end{aligned}$$

Recall that, by the definition of $\bar{\varepsilon}$, $\bar{\varepsilon}^q = \varepsilon$. Because $s(\gamma) \in \{\gamma_1, \ldots, \gamma_N\}$ for all $\gamma \in \Gamma$ and $\exp(\cdot)$ is increasing, the display continues as

$$h^\dagger(x) \leq \exp\left\{\max_{j=1,\ldots,N} \log \frac{dP_{\gamma_j}}{d\lambda}(x) + \varepsilon r(x)\right\} = \max_{j=1,\ldots,N} \exp\left\{\log \frac{dP_{\gamma_j}}{d\lambda}(x) + \varepsilon r(x)\right\}$$

Because $\max_{j=1,\ldots,N} z_j \leq \sum_{j=1}^N z_j$ for all nonnegative $z_1, \ldots, z_N$, the display continues as

$$h^\dagger(x) \leq \sum_{j=1}^N \exp\left\{\log \frac{dP_{\gamma_j}}{d\lambda}(x) + \varepsilon r(x)\right\} = \sum_{j=1}^N \exp\{\varepsilon r(x)\} \frac{dP_{\gamma_j}}{d\lambda}(x)$$

Recalling that the above display holds for all $x \in \mathcal{X}\backslash\mathcal{X}_0$ and also that $\lambda(\mathcal{X}_0) = 0$, we see that

$$\int_{\mathcal{X}} h^\dagger(x)d\lambda(x) \le \int_{\mathcal{X}} \sum_{j=1}^{N} \exp\{\varepsilon r(x)\} \frac{dP_{\gamma_j}}{d\lambda}(x)d\lambda(x) = \sum_{j=1}^{N} \int_{\mathcal{X}} \exp\{\varepsilon r(x)\}\, dP_{\gamma_j}(x)$$

The right-hand side is finite because $N < \infty$ and $\int_{\mathcal{X}} \exp\{\varepsilon r(x)\}\, dP_\gamma(x) < \infty$ for all $\gamma \in \Gamma$ by the choice of ε. Hence, $\int_{\mathcal{X}} h^\dagger(x)d\lambda(x) < \infty$. That is, $\nu^\dagger$ is finite. □

Appendix F. An example showing challenges faced by existing nested maximin algorithms.

We now provide a simple example in which a nested maximin procedure that iteratively updates a discrete prior would not yield a nearly minimax procedure in a reasonable amount of computational time. In this example, the data consist of n draws from a multivariate normal with an unknown mean $\mu \in [-m, m]^p$, $m > 0$, and known positive definite covariance matrix Σ. For a given μ, performance is judged using the sum of squared errors risk $\mathcal{R} : (T, \mu) \mapsto \mathbb{E}_\mu[\|T(X) - \mu\|_2^2]$, where $\|\cdot\|_2$ is the ℓ_2 norm and X is a collection of n independent draws from the multivariate normal with mean μ and covariance matrix Σ. We take the class of allowable procedures $\mathcal{T}$ to be unrestricted. For a procedure $T \in \mathcal{T}$, we denote the maximal risk by $\mathcal{R}^\star(T) \equiv \sup_{\mu\in[-m,m]^p} \mathcal{R}(T, \mu)$. For a subset $\mathcal{D}$ of $[-m, m]^p$, we use $|\mathcal{D}|$ to denote its cardinality and $\mathrm{conv}(\mathcal{D})$ to denote its convex hull. For a discrete prior Π, we let T_Π denote the Bayes procedure with respect to the risk $\mathcal{R}$ and $\mathrm{supp}(\Pi)$ denote the support, that is

$$\mathrm{supp}(\Pi) \equiv \{\mu \in [-m, m]^p : \Pi(\mu) > 0\}$$

In the special case that Σ is the identity matrix, Example 4.6 of (50) shows that a discrete least favorable prior exists in this problem. In the more general case, Theorem 2.3 in (5) shows that a least favorable prior exists. This latter result is proven by constructing a sequence of finitely supported discrete priors that converge weakly to a least favorable prior.

Based on these results, it may seem reasonable to try to numerically learn a discrete unfavorable prior in this problem and to subsequently hope that the Bayes procedure under this prior will be nearly minimax. In the special case that Σ is the identity matrix, Example 4.6 of (50) already suggests that this numerical strategy may perform poorly – in particular, the least favorable prior $\Pi^\star$ presented in that example corresponds to the product measure derived from the p least favorable priors for estimating each component of

μ. By (42), each of these priors is discrete with at least two support points. Hence, the $\Pi^\star$ exhibited in Example 4.6 of (50) has at least 2^p support points. Consequently, if the nested maximin algorithm of (4) is used to iteratively update a discrete prior, as is proposed following Eq. 10 in that reference, then it will require at least order 2^p iterations to learn $\Pi^\star$. Notably, this existing result for the special case that Σ is the identity matrix does not guarantee that the Bayes procedures under discrete priors with fewer than 2^p support points will have poor maximal risk. Therefore, this existing result does not concretely show that nested maximin algorithms that iteratively update a discrete prior will generally require order 2^p iterations to learn a nearly minimax procedure.

In what follows, we will establish that, regardless of the value of Σ, it will be challenging to learn discrete unfavorable priors in practice. In particular, the following theorem gives conditions under which the maximal risk of the Bayes procedure under a discrete prior is considerably larger than the minimax risk unless the prior has many support points. As a consequence, the method of (4) will require an unreasonable number of iterations to learn a discrete prior whose Bayes procedure has nearly maximal risk.

Theorem S6. *Fix $c \geq 1$ and let Π be a prior for which $|\operatorname{supp}(\Pi)| < 2^p$. If m, n, p, and Σ are such that $\operatorname{trace}(\Sigma) \times cp < m^2 n$, then $\mathcal{R}^\star(T_\Pi) > c \inf_{T\in\mathcal{T}} \mathcal{R}^\star(T)$.*

The proof of Theorem S6 is at the end of this section. If Σ is the identity matrix and m is fixed, then the condition of the theorem is satisfied if n is large and $p = o(n^{1/2})$. It is not clear if this requirement is actually needed to guarantee poor worst-case performance of Bayes procedures under priors without exponentially many support points in p, or if this condition is only required due to the proof technique that we use.

We note that the above theorem does not provide any guarantees about what form a *continuous* prior Π would need to take for the corresponding Bayes procedure to have nearly optimal maximal risk. Consequently, the above theorem does not provide any guarantees when the initial prior in the algorithm of (4) is continuous. This lack of guarantee may be due to the limits of the technique of proof. We hope that future work can reveal whether it is computationally feasible for the algorithm of (4) to learn a nearly least favorable prior in this problem when p is large and the initial prior is chosen to be diffuse, e.g. uniform on $[-m, m]^p$. We expect that many iterations would generally be required.

In our discussions above, we chose to focus on the case that the observations are multivariate normal

because there are several existing works studying this case, thereby allowing us to put our results into historical context. Nonetheless, we note that the proofs of the upcoming Lemmas S7 and S8 do not make use of the fact that the observations were drawn from a multivariate normal distribution. We also do not use the fact that the observations are multivariate normal in the proof of Theorem S6 – indeed, Theorem S6 remains valid if the statistical model consists of distributions of n independent observations drawn from a distribution belonging to a collection $\mathcal{Q}_1 \subseteq \mathcal{Q}$, where $\mathcal{Q}$ is the collection of distributions on $\mathbb{R}^p$ with mean μ and known covariance matrix Σ. The only restriction that we require on $\mathcal{Q}_1$ is that, for all vertices μ of the hypercube $[-m, m]^p$, there must exist a distribution in $\mathcal{Q}_1$ with mean μ. Hence, our results can be used to show that a discrete prior must have exponentially many support points for the corresponding Bayes estimator to be nearly minimax optimal in a wide variety of multivariate mean estimation settings.

We now establish two lemmas that we use to prove Theorem S6. In what follows, we let $\mathcal{V}$ denote the set of vertices of the hypercube $[-m, m]^p$. We let $\|\cdot\|_1$ denote the ℓ_1 norm.

Lemma S7. *Let Π be a discrete prior on the mean $\mu \in [-m, m]^p$. If $|\operatorname{supp}(\Pi)| < 2^p$, then there exists a $\mu_0 \in \mathcal{V}$ such that $\min_{\mu \in \operatorname{supp}(\Pi)} \|\mu - \mu_0\|_1 \geq m$.*

Proof. For each $\mu_1 \in \mathcal{V}$, let $\operatorname{proj}_\Pi(\mu_1)$ denote a minimizer of $\|\mu - \mu_1\|_1$ over $\mu \in \operatorname{supp}(\Pi)$, where an arbitrary value is selected if there is not a unique minimizer. Define $\mathcal{V}_1 \equiv \{\operatorname{proj}_\Pi(\mu_1) : \mu_1 \in \mathcal{V}\}$, and note that $\mathcal{V}_1 \subseteq \operatorname{supp}(\Pi)$. Hence, $|\mathcal{V}_1| \leq |\operatorname{supp}(\Pi)| < 2^p$. Because $|\mathcal{V}| = 2^p$, it follows that there exist two distinct vertices μ_1 and μ_2 in $\mathcal{V}$ such that $\operatorname{proj}_\Pi(\mu_1) = \operatorname{proj}_\Pi(\mu_2)$. Using that $\|\mu_1 - \mu_2\|_1 \geq 2m$, the triangle inequality, the fact that $\mathcal{V}_1 \subseteq \operatorname{supp}(\Pi)$, and the fact that $\mu_1, \mu_2 \in \mathcal{V}$, we see that

$$
\begin{aligned}
2m &\leq \|\mu_1 - \mu_2\|_1 \leq \|\operatorname{proj}_\Pi(\mu_1) - \mu_1\|_1 + \|\operatorname{proj}_\Pi(\mu_2) - \mu_2\|_1 \\
&= \min_{\mu \in \operatorname{supp}(\Pi)} \|\mu - \mu_1\|_1 + \min_{\mu \in \operatorname{supp}(\Pi)} \|\mu - \mu_2\|_1 \\
&\leq 2 \max_{\mu_0 \in \mathcal{V}} \min_{\mu \in \operatorname{supp}(\Pi)} \|\mu - \mu_0\|_1
\end{aligned}
$$

Dividing both sides by two shows that $\max_{\mu_0 \in \mathcal{V}} \min_{\mu \in \operatorname{supp}(\Pi)} \|\mu - \mu_0\|_1 \geq m$. Hence, there exists a $\mu_0 \in \mathcal{V}$ such that $\min_{\mu \in \operatorname{supp}(\Pi)} \|\mu - \mu_0\|_1 \geq m$. □

Lemma S8. *Fix p and n. For any prior Π for which $|\operatorname{supp}(\Pi)| < 2^p$, it holds that $\mathcal{R}^\star(T_\Pi) \geq m^2/p$.*

Proof. Fix a distribution Π for which $|\operatorname{supp}(\Pi)| < 2^p$. Let μ_0 be a vertex in $\mathcal{V}$ for which $\min_{\mu\in\operatorname{supp}(\Pi)} \|\mu - \mu_0\|_1 \geq m$; such a μ_0 is guaranteed to exist by Lemma S7. Note that

$$\begin{aligned}\operatorname{supp}(\Pi) &\subseteq \{\mu \in [-m,m]^p : \|\mu-\mu_0\|_1 \geq m\} \\ &\subseteq \left\{\mu \in [-m,m]^p : \|\mu-\mu_0\|_2^2 \geq m^2/p\right\}\end{aligned}$$

where the latter inclusion follows from the fact that $p^{1/2}\|z\|_2 \geq \|z\|_1$ for all $z \in \mathbb{R}^p$. Hence,

$$\operatorname{conv}\circ\operatorname{supp}(\Pi) \subseteq \operatorname{conv}\left\{\mu \in [-m,m]^p : \|\mu-\mu_0\|_2^2 \geq m^2/p\right\}$$

It is well known that the Bayes procedure T_Π in this problem corresponds to the posterior mean of μ given $X = x$. As the support of the posterior distribution is contained in $\operatorname{supp}(\Pi)$, the range of the posterior mean is a subset of $\operatorname{conv}\circ\operatorname{supp}(\Pi)$. Combining this fact with the above display, this implies that, for all data sets x, $\|T_\Pi(x)-\mu_0\|_2^2 \geq m^2/p$. Hence, $\mathbb{E}_{\mu_0}[\|T_\Pi(X)-\mu_0\|_2^2] \geq m^2/p$. The proof concludes by noting that $\mathcal{R}^\star(T_\Pi) \geq \mathcal{R}(T_\Pi,\mu_0) \equiv \mathbb{E}_{\mu_0}[\|T_\Pi(X)-\mu_0\|_2^2]$. □

We now prove Theorem S6. In the proof, we denote the procedure that returns the p-dimensional sample mean of the n observations $X = (X_1,\ldots,X_n)$ by T_s. We use the convention that $T_s(X)$ and μ are column vectors.

Proof of Theorem S6. Let Π be a prior such that $|\operatorname{supp}(\Pi)| < 2^p$. Fix $c \geq 1$, and fix m, n, p, and Σ be such that $\operatorname{trace}(\Sigma)\times c/n < m^2/p$. By Lemma S8, $\mathcal{R}^\star(T_\Pi) \geq m^2/p$. For all $\mu \in [-m,m]^p$, it is true that

$$\begin{aligned}\mathcal{R}(T_s,\mu) &= \mathbb{E}\left[(T_s(X)-\mu)^\top (T_s(X)-\mu)\right] = \mathbb{E}\left[\operatorname{trace}\left\{(T_s(X)-\mu)^\top (T_s(X)-\mu)\right\}\right] \\ &= \mathbb{E}\left[\operatorname{trace}\left\{(T_s(X)-\mu)(T_s(X)-\mu)^\top\right\}\right] = \operatorname{trace}\left(\mathbb{E}\left[(T_s(X)-\mu)(T_s(X)-\mu)^\top\right]\right) \\ &= \frac{1}{n}\operatorname{trace}\left(\mathbb{E}\left[(X_1-\mu)(X_1-\mu)^\top\right]\right) = \frac{\operatorname{trace}(\Sigma)}{n}\end{aligned}$$

By the choice of m, n, p, and Σ, it follows that $\mathcal{R}^\star(T_\Pi) > c\mathcal{R}^\star(T_s) \geq c\inf_{T\in\mathcal{T}}\mathcal{R}^\star(T)$. □

Appendix G. Captions for additional file types.

Movie S1. This movie displays the evolution of the risk of the learned estimator of μ as the weights of the neural network are updated in the Gaussian model with $n = 50$ observations and unknown (μ,σ). Risk is

displayed across the parameter space. The horizontal plane indicates the maximal risk of the estimator at a given iteration.

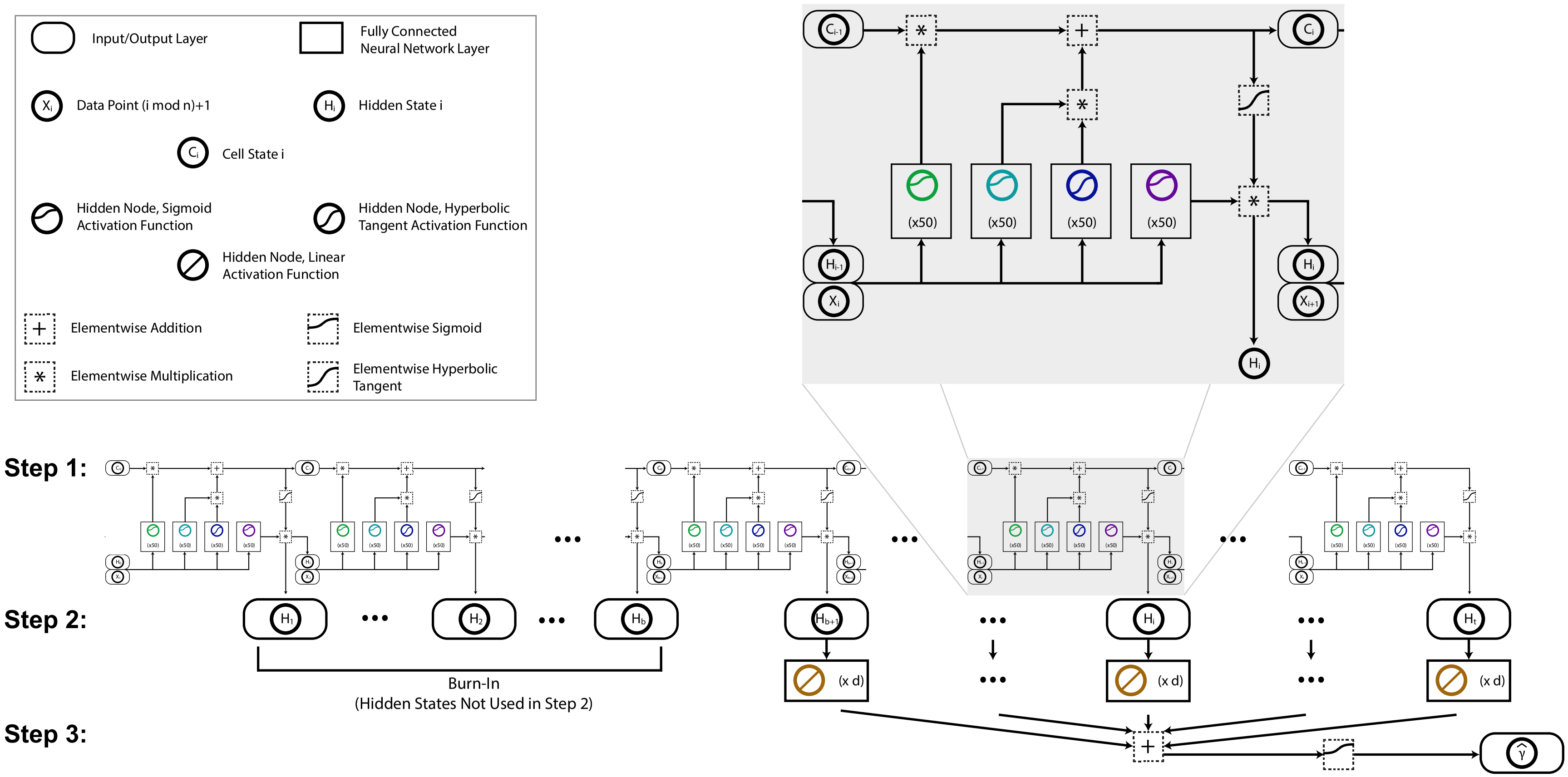

Fig. S6. Estimator LSTM used when estimating binary regressions. Hidden nodes of the same color have identical weights. The algorithm proceeds in three steps. First, the n observations are sequentially fed to the LSTM one or more times, and a total of $t = 3n/2$ hidden states are computed. Some of the early hidden states are discarded as burn-in ($b = n/2$). Second, the undiscarded hidden states are linearly transformed to the dimension d of the parameter. Finally, these linear transformations are summed and transformed elementwise via a rescaled sigmoid function to respect the known bounds on the regression coefficients. Repeating module diagram modeled after `http://colah.github.io/posts/2015-08-Understanding-LSTMs/`.

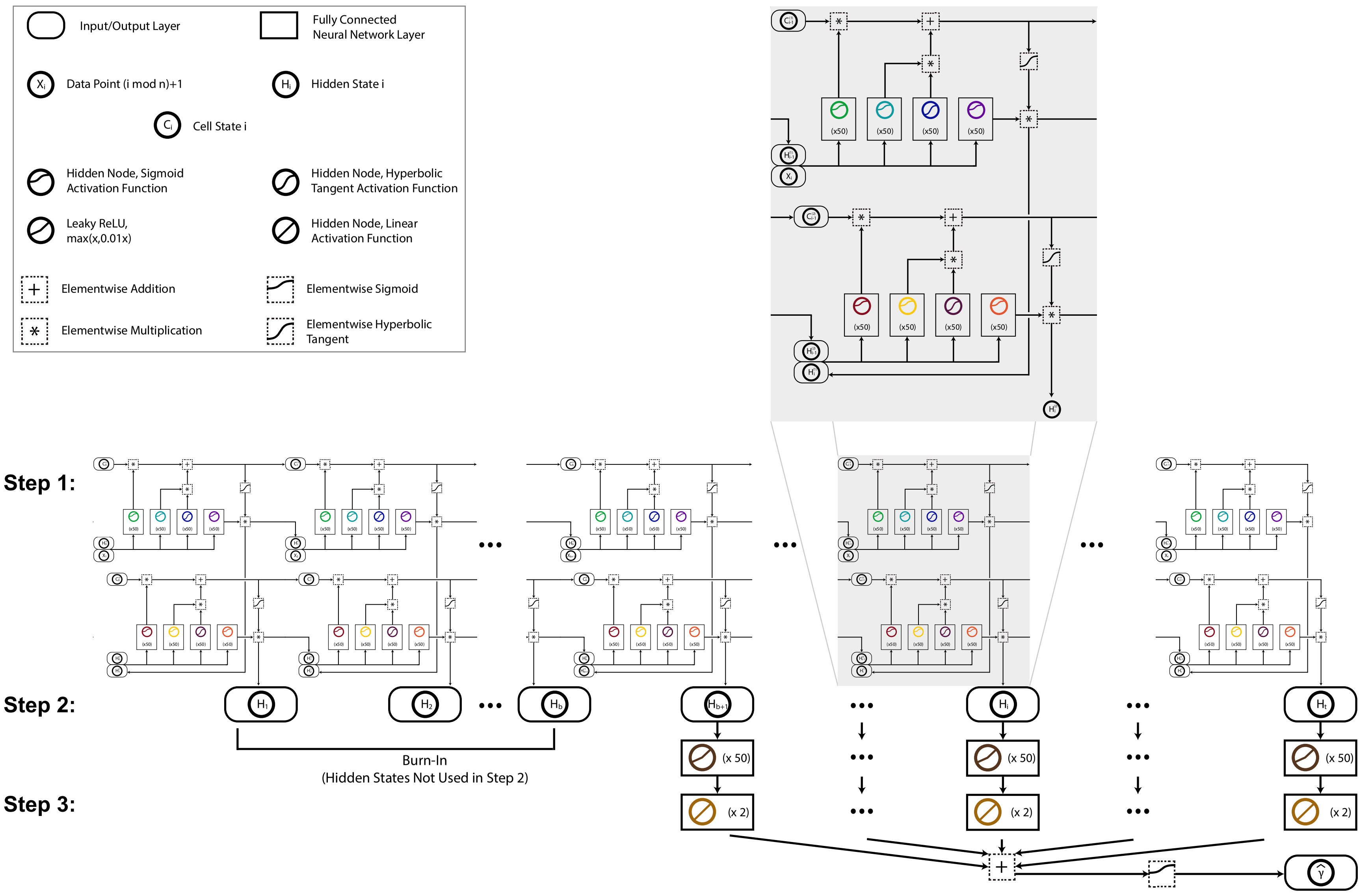

Fig. S7. Estimator LSTM used when defining the interior point of our confidence regions. Hidden nodes of the same color have identical weights. The cell state and history state were both initialized to 50×50 zero matrices. The algorithm proceeds in three steps. First, the n observations are sequentially fed through two LSTM layers over two cycles through the n observations, and a total of $t = 2n$ hidden states are returned. The hidden states corresponding to the first $b = n$ history states are used as burn-in, and in particular are only used only to initialize the cell state. Second, the undiscarded hidden states are passed through a multilayer preceptron that outputs a two-dimensional vector for each observation. Finally, these linear transformations are summed and output an estimate of $\gamma = (\mu, \sigma)$. Repeating module diagram modeled after `http://colah.github.io/posts/2015-08-Understanding-LSTMs/`.
